
JOURNAL INFORMATION
==============================
NLM Title Abbreviation: Innov Surg Sci
Journal ID: Innov Surg Sci
NLM Journal ID: 101708165
Journal ID: iss

Innovative Surgical Sciences

EISSN: 2364-7485
Publisher: De Gruyter

ARTICLE INFORMATION
==============================
PMCID: PMC10216890
DOI: 10.1515/iss-2023-9009
Article ID: iss-2023-9009
Article version: 1
Subjects: Article

Abstract DGCH

Publication date: 2023 Mar
Electronic publication date: 2023 Mar 13
Volume: 8
Issue: Suppl1
First page: 216
Last page: 322
Copyright: © the Author(s), published by De Gruyter
Copyright year: 2023
Copyright holder: the Author(s), published by De Gruyter
License: This work is licensed under the Creative Commons Attribution 4.0 International License.
License URL: https://creativecommons.org/licenses/by/4.0/

Page count: 107

==============================

**Gastrectomy for cancer beyond life expectancy. A comprehensive analysis of oncological gastric surgery in Germany between 2008 and 2018**
ID: 667

**Kategorie: DGCH - Chirurgie im hohen Lebensalter**

Daniel Reim[1], Maximilian Berlet[1], Helmut Friess[1]

[1]*Klinikum rechts der Isar der Technischen Universität München, Department of General and Visceral Surgery, München, Deutschland*

**Background:**
Major gastric surgery for distal esophageal and gastric cancer has a strong impact on quality of life, morbidity, and mortality. Especially in elderly patients reaching their life expectancy, responsible use and extent of gastrectomy is imperative to achieve a balance between harm and benefit. In the present study, the reimbursement database (G-DRG database) of the Statistical Office of the Federal Republic of Germany was queried to evaluate morbidity and mortality of patients aged above or below 75 years following gastrectomy.

**Materials and methods:**
All patients in Germany undergoing subtotal gastrectomy (ST), total gastrectomy (T), or gastrectomy combined with esophagectomy (TE) for gastric or distal esophageal cancer (ICD-10 C15.2, C15.5, C16.0-C16.9) between 2008 and 2018 were included. Intraoperative and postoperative complications as well as comorbidities, in-hospital mortality and extent of surgery were assessed by evaluating ICD-10 and operation and procedure key (OPS) codes.

**Results:**
A total of 67389 patients underwent oncologic gastric resection in Germany between 2008 and 2018. 21794 patients received ST, 41825 T, and 3466 TE respectively. In 304 cases, combinations of these in fact mutually exclusive procedures were encoded. The proportion of patients aged 75 years or older was 51.4% (n=11207) for ST, 32.6% (n=13617) for T, and 28.1% (n=973) for TE. In-hospital mortality of elderly patients was significantly increased in all three groups. ($p < 0.0001$) General complications such as respiratory failure ($p = 0.0054$), acute renal failure ($p < 0.0001$) acute myocardial failure ($p < 0.0001$), and the need for resuscitation (ST/T: $p < 0.0001$ / TE: $p = 0.0218$) were significantly increased after any kind of gastrectomy. Roux-en Y was the most commonly applied reconstruction technique in both young and elderly. Regarding lymphadenectomy (LAD), systematic D2 dissection was performed less frequently in older patients than in the younger collective in case of ST and T as well as D3 dissection. Peritonectomy and HIPEC were uncommon in elderly alongside ST and T compared to younger patients. ($p < 0.0001$).

**Conclusion:**
The clinical outcome of major oncological gastric surgery is highly dependent on patient’s age. Elderly show a tremendously increased likelihood of in-hospital mortality and morbidity.

Open Access. © 2023 The Author(s), published by De Gruyter. This work is licensed under the Creative Commons Attribution 4.0 International License.

**Serum cytokine profile after extended partial hepatectomy**
ID: 692

**Kategorie: DGCH - Chirurgie im hohen Lebensalter**

Eva Kindler[1,2], Sandra Nickel[1], Uta Dahmen[1], Hans-Michael Tautenhahn[1], Utz Settmacher[1]

[1]*Klinik für Allgemein-, Viszeral und Gefäßchirurgie am Universitätsklinikum Jena, Jena, Deutschland*, [2]*Else Kröner-Promotionskolleg Jena School for ageing medicine (JSAM), Universitätsklinikum Jena, Jena, Deutschland*

**Background:**
The incidence of primary and secondary neoplasms of the liver increases markedly with age. In many cases, extensive partial liver resection is the only curative treatment option. Although chronological age alone is not a contraindication, elderly patients have a substantially increased risk of postoperative complications. The outcome is influenced by the operation itself and the underlying liver disease, but also by the age and comorbidities of the patient. All these factors are reflected by the immune response before and during partial liver resection. On the other hand, a coordinated interplay of cytokines and growth factors is required for postoperative liver regeneration. A deviation of these processes due to an age-related alteration in the cytokine profile (inflamm-aging) represents a possible explanation for the higher complication rate after partial liver resection and is therefore characterized in more detail in the following project.

**Materials and methods:**
Patients at Jena University Hospital undergoing extended partial liver resection were included in this two-armed prospective unicenter study. (I) young patients between 18 and 45 years (n=7) and (II) older patients ≥65 years (n=15). Serum samples were collected preoperatively, and on postoperative day 1 and 2. The cytokine profile including 105 cytokines and growth factors was determined using the semiquantitative Human XL Cytokine Array (R&D Systems®). Five additional essential parameters in the context of liver regeneration (IL6, THBS1, TGF-beta1, IGF1 and THPO) were quantified using ELISA kits. Subsequently, a bioinformatical analysis was performed to map the relationships and interactions between the identified cytokines. More than 2000 clinical parameters such as comorbidities and the preoperative LiMAx® assay were considered in the analysis of clinical data and collected using an SQL database.

**Results:**
Preliminary results show a marked upregulation of ST2 (soluble IL-33 receptor) and downregulation of EGF on the first and second postoperative day independent of patient age. Despite a high interindividual variability in the pre- and postoperative cytokine profile, age-dependent differences emerge especially for HGF, IL-6, IGF-1 and THPO. HGF is upregulated postoperatively, and elderly patients show higher values on day 2 after the operation. THPO levels were significantly higher in old patients at all timepoints *(e.g. old: 316,8pg/ml (CI 295,2 – 340,8) vs. young: 268,8pg/ml (CI 252,7 – 296,5), p<0,001 in preoperative samples)*. For IL6 we could show higher preoperative concentrations *(old: 8,7pg/ml (CI 4,5 – 14,2) vs. young: 2,0pg/ml (CI 1,5 – 3,8), p<0,001)* and a delayed increase in the group of elderly patients. IGF-1 levels showed a significantly stronger postoperative decrease in old patients *(old: 37,0ng/ml (CI 29,0 – 55,0) vs. young: 76,0ng/ml (CI 46,0 – 116,0), p<0,01 on POD1)*.

**Conclusion:**
Most changes when comparing pre- and postoperative cytokine profiles can be attributed to the surgery itself and interpreted an expression of the body's stress response to surgery and resection-related functional impairment of the liver. The dysregulation of parameters relevant for liver regeneration (HGF, IL6, IGF1) seen predominantly in elderly patients may provide a possible explanation for impaired hepatic recovery after partial liver resection with increasing age. However, the high interindividual variability suggests that cytokines can only be interpreted in the context of clinical data.

**Bladed and bladeless conical trocars do not differ in terms of caused fascial defect size in a Porcine Model**
ID: 38

**Kategorie: DGCH - Chirurgische Innovation, Grundlagenforschung**

Christoph Paasch[1], Richard Hunger[1], Anne Mantke[1], Rene Mantke[1], Sophie Heisler[1]

[1]*Universitätsklinikum Brandenburg an der Havel, Brandenburg an der Havel, Deutschland*

**Background:**
Trocar insertion during laparoscopy may lead to complications such as bleeding, bowel puncture and fascial defect with subsequent trocar site hernias. It is under discussion whether there is a difference in the extent of the trauma and thus in the size of the fascia defect between blunt and sharp trocars. But the level of evidence is low. Hence, we performed a Porcine Model.
Conclusion Bladed and bladeless conical 12-mm trocars do not differ in terms of caused fascial defect size in the Porcine Model at hand.

**Materials and methods:**
A total of five euthanized female pigs were operated on. The average weight of the animals was 37.85 (Standard deviation SD 1.68) kg. All pigs were aged 90 ± 5 days. In alternating order five different conical 12-mm trocars (3 × bladeless,2 × bladed) on each side 4 cm lateral of the mammary ridge were placed. One surgeon performed the insertions after conducting a pneumoperitoneum with 12 mmHg using a Verres' needle. The trocars were removed after 60 min. Subsequently, photo imaging took place. Using the GSA Image Analyser (v3.9.6) the respective abdominal wall defect size was measured.

**Results:**
The mean fascial defect size was 58.3 (SD 20.2) mm2. Bladed and bladeless trocars did not significant differ in terms of caused fascial defect size [bladed, 56.6 (SD 20) mm2 vs. bladeless, 59.5 (SD 20.6) mm2, p = 0.7]. Without significance
the insertion of bladeless trocars led to the largest (Kii Fios™ First entry, APPLIEDMEDICAL©, 69.3 mm2) and smallest defect size (VersaOne™ (COVIDIEN©, 54.1 mm2).

**Conclusion:**
The occurrence of a trocar site hernia might be largely independent from trocar design.

**Development and Preclinical Evaluation of a Novel 3D-printed Fixation Device for the Disrupted Pubic Symphysis in Pelvic Trauma**
ID: 106

**Kategorie: DGCH - Chirurgische Innovation, Grundlagenforschung**

Martin Jordan[1], David Bröer[1], Christian Fischer[2], Philipp Heilig[1], Fabian Gilbert[3], Stefanie Hoelscher-Doht[1], Charis Kalogirou[1], Kevin Popp[4], Jan-Peter Grunuz[1], Henner Huflage[1], Rafael Jakubietz[1], Süleyman Ergün[5], Rainer Meffert[1]

[1]*Würzburg, Universitätsklinikum Würzburg, Würzburg, Deutschland*, [2]*Unterpleichfeld, Headmade Materials, Unterpleichfeld, Deutschland*, [3]*München, Klinik für Orthopädie und Unfallchirurgie, LMU, München, Deutschland*, [4]*Würzburg, SKZ, Würzburg, Deutschland*, [5]*Institut für Anatomie, Universität Würzburg, Würzburg,*

**Background:**
Traumatic separation of the pubic symphysis can destabilize the pelvis and require surgical fixation to reduce symphyseal gapping. The traditional Pfannenstiel surgical approach involves implantation of a steel symphyseal plate (SP) through a horizontal incision proximal to the pubic tubercle. Despite its widespread use, SP fixation requires invasive surgery, and implant failure caused by screw loosening or breakage is common. To address the need for a less invasive and more reliable surgical intervention, we manufactured two new titanium cable-clamp implants using innovative three-dimensional printing technology based on cold metal fusion.

**Materials and Methods & Results:**
The first implant design included a steel cable anterior to the pubic symphysis to simplify its placement outside the pelvis, and the second design included a cable encircling the pubic symphysis to stabilize the anterior pelvic ring. Using highly reproducible synthetic bone models and a limited number of cadaver specimens, we performed a comprehensive biomechanical study of implant stability and showed that the new cable-clamp implants provided stabilities equivalent to that of a traditional SP but without the same risks of implant failure. Since neither device could be implanted using a Pfannenstiel incision, we also performed detailed ex vivo evaluations of the safety and feasibility of a new, less-invasive trans-obturator surgical approach.

**Conclusion:**
We propose that the new cable-clamp fixation devices may be of clinical value in treating pubic symphysis separation.

**Pharmacologic HIF-1 Inhibition mitigates postoperative adhesion formation**
ID: 174

**Kategorie: DGCH - Chirurgische Innovation, Grundlagenforschung**

Moritz J. Strowitzki[1], Marvin L. Biller[1], Marc Bleul[1], Christopher Tuffs[1], Martin Schneider[1]

[1]*Heidelberg University, Department of General, Visceral and Transplantation Surgery, Heidelberg, Deutschland*

**Background:**
Peritoneal adhesions (PA) occur after abdominal surgery and can lead to severe complications. Until today no feasible prevention strategies exist. Preclinical data suggest that the stabilization of hypoxia-inducible factors (HIF) plays a key role in adhesion formation. We thus determined the clinical significance of HIF signaling during adhesion formation and investigated whether pharmacologic HIF inhibition might be a promising treatment option to prevent PA.

**Materials and methods:**
During the ReLap study (DRKS00013001), adhesive tissue from patients undergoing relaparotomy was harvested and graded using the adhesion grade score. HIF-1 signaling activity within tissue biopsies was determined and correlated with adhesion severity. Human mesothelial cells (LP-9) and murine fibroblasts were treated with different pharmacologic HIF inhibitors or HIF-1 siRNA under normoxia and hypoxia. HIF-1α and -2α levels were determined. mRNA levels of pro-adhesiogenic HIF-target genes were assessed. PA were induced in mice treated with different HIF-inhibitors or vehicle daily and evaluated after 7 days.

**Results:**
HIF-1 signaling activity correlated with adhesion severity in patient biopsies. Pharmacologic HIF inhibition decreased HIF-1α protein levels in murine fibroblasts under hypoxia. *In vivo*, HIF inhibition decreased PA quantity and tenacity. Moreover, HIF inhibition mitigated immune cell infiltration within adhesion tissue. Pharmacologic and genetic HIF inhibition significantly reduced adhesion-fostering HIF-target genes in LP-9 cells and fibroblasts. LP-9 cells were less activated under hypoxia and simultaneous HIF inhibition. In contrast, cell viability was not affected by pharmacological HIF-1 modulation.

**Conclusion:**
Pharmacologic HIF inhibition mitigates adhesion formation by inhibiting HIF-1-dependent (myo)fibroblast activation, conferring an antiadhesive microenvironment after abdominal surgery. Therefore, repurposing of clinically approved drugs inhibiting HIF-1 may represent a novel therapeutic strategy to prevent PA.

**Pharmacological inhibition of HIF-Prolylhydroxylases improves intestinal healing of critical colorectal anastomoses**
ID: 175

**Kategorie: DGCH - Chirurgische Innovation, Grundlagenforschung**

Moritz J. Strowitzki[1], Marvin Biller[1], Marc Bleul[1], Christopher Tuffs[1], Martin Schneider[1]

[1]*Heidelberg University, Department of General, Visceral and Transplantation Surgery, Heidelberg, Deutschland*

**Background:**
Anastomotic leakage (AL) is a potentially life-threatening complication after colorectal surgery. Local ischemia and inflammation play a central role during the development of AL. As a result, HIF-α subunits are stabilized and the HIF-pathway is up-regulated, protecting tissue from oxygen deprivation. Notably, the HIF-pathway can be induced by pharmacological inhibition of HIF-Prolylhydroxylases (PHD) 1-3 (PHI) via Dimethyloxalylglycine (DMOG). We, thus, investigated the role of the PHDs and PHI in AL.

**Materials and methods:**
We induced AL in wildtype (WT) and PHD1-3-knockout (KO) mice by ligation of key blood vessels nourishing anastomosed colonic segments or inducing abdominal sepsis by lipopolysaccharides injection. Mice were treated daily with DMOG. Anastomotic burst pressure (BP) and abscess formation were determined. Specimens were taken for immunohistochemical analysis of important HIF-target genes involved in angiogenesis, acute inflammation, and epithelial-mesenchymal-transition (EMT). Macrophage polarization (M1 *versus* M2) was analyzed *in vivo* and *in vitro*.

**Results:**
DMOG treatment and partial loss of PHD2 gene function ($PHD2^{+/-}$) markedly attenuated AL and increased anastomotic BP indicating improved intestinal healing. Immunohistochemistry of anastomotic tissues revealed decreased invasion of inflammatory cells and increased EMT-related collagen content. Angiogenesis was not influenced by DMOG or $PHD2^{+/-}$. Proteome profiling after DMOG treatment revealed induced M2 macrophage polarization. M2 markers were induced by PHI and $PHD2^{+/-}$ in murine macrophages and primary isolated macrophages.

**Conclusion:**
PHI protects from AL of critical anastomoses and improves intestinal healing. This effect is elicited by an inhibition of PHD2-related M2 polarization of macrophages conferring a favorable micro-environment for anastomotic healing.

**Macrophages Possess Diverse Roles During Different Phases of Intestinal Anastomotic Healing**
ID: 187

**Kategorie: DGCH - Chirurgische Innovation, Grundlagenforschung**

Maximiliane Winter[1], Benjamin Struecker[1], Barbara Heitplatz[2], Georg Varga[3], Andreas Pascher[1], Emile Rijcken[1], Felix Becker[1]

[1]*Universitätsklinikum Münster, Klinik für Allgemein-, Viszeral- und Transplantationschirurgie , Münster, Deutschland*, [2]*Universitätsklinikum Münster, Gerhard-Domagk-Institut für Pathologie, Münster, Deutschland*, [3]*Universitätsklinikum Münster, Klinik für Pädiatrische Rheumatologie und Immunologie, Münster, Deutschland*

**Background:**
Intestinal anastomotic healing (AH) following colonic resection is the most crucial step in colorectal surgery. Disruptive AH consequently leads to anastomotic leakage, a feared postoperative complication, associated with high morbidity and mortality. Intestinal AH is a complex and tightly organized process, classically divided into three overlapping phases: (I) an inflammatory, (II) a proliferative, and (III) a reparative phase. In this continuum, coagulation and rapid immune cell recruitment are followed by increased angiogenesis, mucosal re-epithelialization, collagen synthesis, and lastly, a prolonged phase of collagen remodeling and reorganization of the extracellular matrix. Each phase is temporally defined and characterized by specific hallmark events, elicited by dynamic interactions between resident immune, endothelial, stroma and epithelial cells as well as infiltrated leukocytes. Among them, monocytes and macrophages represent a key subset. Understanding their phase-specific function and plasticity during intestinal AH is urgently needed to better understand disrupted AH.

**Materials and methods:**
We used transgenic CD11b-DTR (B6.FVB-Tg (ITGAM-DTR/EGFP)34Lan/J) mice, in which monocytes and macrophages can be specifically depleted by diphtheria toxin (DT) injections. To study intestinal AH, a model of distal colonic end to end anastomosis was performed on CD11b-DTR as well as on wild type (WT) mice. After surgery, mice were stratified in groups (defined by the length of the post-operative period) and phase-specific DT-induced monocyte and macrophage depletion was conducted during either the inflammatory (day 0-3), proliferative (day 4-10), or reparative (day 11-20) phase of intestinal AH. Systemic circulating monocyte and local intestinal macrophage burden was determined by FACS and immunofluorescence. Intestinal AH was quantified by a specific histological score and by anastomotic bursting pressure. For each phase a key hallmark pathway (inflammation, angiogenesis and extracellular matrix remodeling) was evaluated.

**Results:**
Firstly, we could demonstrate that on-demand DT-induced systemic monocyte and local macrophage depletion was successfully coupled with the surgical model of distal colonic end to end anastomosis, irrespective of the evaluated phase. Next, we investigated AH in the inflammatory (post-operative day 3) phase. At this early stage, AH had not yet advanced, but the inflammatory response in CD11b-DTR mice was significantly reduced. When comparing AH the proliferative (post-operative day 10) phase, CD11b-DTR mice revealed a significantly improved histological healing score although these mice displayed disrupted angiogenesis. Finally, analysis of AH in the reparative phase (post-operative day 20) revealed no difference in histological healing or anastomotic bursting pressure. However, CD11b-DTR mice showed a reduced genetic expression of mediators (e.g. TGF-beta) of extracellular matrix remodeling.

**Conclusion:**
The here presented results support the hypothesis that monocytes and macrophages elicit phase-specific effects during intestinal AH. Our data further supports the mounting evidence that these cells play a dual role by exhibiting both pro-inflammatory as well as pro-resolving properties. Thus, when these cells are targeted during therapeutic interventions in intestinal AH, their phase-specific effects must be considered critically.

**Sulforaphane alleviates kidney injury by regulating autophagy and apoptosis in the murine hemorrhagic shock/resuscitation model**
ID: 283

**Kategorie: DGCH - Chirurgische Innovation, Grundlagenforschung**

You LI[1], Kang Qin[1], Weiqiang Liang[1,2], Qun Zhao[1], Athanassios Fragoulis[3], Christoph Wruck[3], Thomas Pufe[3], Philipp Kobbe[1], Elizabeth Balmayor[1], Frank Hildebrand[1], Philipp Lichte[1], Johannes Greven[1]

[1]*Department of Orthopaedics, Trauma, and Reconstructive Surgery, RWTH Aachen University Hospital, Aachen, Germany., Aachen, Deutschland*, [2]*Department of Bone and Joint Surgery, The First Affiliated Hospital of Shandong First Medical University, Jinan, China., Jinan, Deutschland*, [3]*Department of Anatomy and Cell Biology, RWTH Aachen University Hospital, Aachen, Germany., Aachen, Deutschland*

**Background:**
Autophagy is essential for maintaining renal cell survival and cellular homeostasis. Hypoxia, adenosine triphosphate (ATP) depletion, and organelle damage during kidney injury account as autophagy activators. Renal cells can degrade harmful substances and clear damaged organelles by the process of autophagy. Sulforaphane (SFN), as an agonist of nuclear factor-erythroid factor 2-related factor 2 (Nrf2) signaling pathway, has also been found to regulate the level of autophagy in cells in addition to its antioxidant capacity. This study investigated the effect of SFN on the autophagy and apoptosis levels in renal tissue of a hemorrhagic shock/resuscitation (HS/R) model in mice.

**Materials and methods:**
Male C57/BL6 mice were randomly divided into four groups including sham group, SFN group, HS/R group and HS/R+SFN group (registered at the State Office for Nature, Environment and Consumer Protection North Rhine-Westphalia (LANUV) 84-02.04.2017.A252). The HS was maintained by pressure-controlled arterial blood withdrawal (35-45mmHg) for 90 min. Fluid resuscitation was performed by reinfusion of withdrawn blood and 0.9% saline. SFN or 0.9% saline (vehicle) were subsequently administrated intraperitoneally. Mice were sacrificed 24h after resuscitation. Serum and renal tissue were collected after the operation. Western blots, ELISAs, and histological examinations were performed.

**Results:**
SFN reduced HS/R-induced blood urea, nitrogen, and serum creatinine. The results of H&E staining showed swollen renal tubules. In addition, renal casts appeared in HS/R mice, while SFN alleviated the pathological changes in mouse renal tissue. The results of western blot and immunohistochemistry staining showed that SFN could significantly reduce the expression of cleaved-caspase 3 and increase the ratio of anti-apoptotic protein B-cell lymphoma 2 (Bcl2) and Bcl-2-associated X protein (Bax) (Bcl2/Bax). We found significant autophagosomes in the kidney tissue of HR/R mice by electron microscopy, and SFN inhibited the formation of autophagosomes. In addition, SFN promoted the formation of autophagy-related proteins beclin-1 and inhibited P62, as well as increased the ratio of LC3-II and LC3-I.

**Conclusion:**
SFN effectively promoted the level of autophagy in renal cells, thereby alleviating HS/R-induced kidney injury and renal cell apoptosis in mice.

**Intraoperative photodynamic diagnostics using hypericin for gastric cancer – a pilot study**
ID: 286

**Kategorie: DGCH - Chirurgische Innovation, Grundlagenforschung**

Can Yurttas[1], Markus Löffler[1,2,3,4,5], Karolin Thiel[1], Ruth Ladurner[1], Ingmar Königsrainer[1,6], Alfred Königsrainer[1,3,4,4], Philipp Horvath[1], Stefan Beckert[1,7]

[1]*Universitätsklinikum Tübingen, Allgemeine, Viszeral- und Transplantationschirurgie, Tübingen, Deutschland*, [2]*Universitätsklinikum Tübingen, Abteilung für klinische Pharmakologie, Tübingen,*, [3]*Universität Tübingen, Deutsches Konsortium für translationale Krebsforschung (DKTK) und Deutsches Krebsforschungszentrum (DKFZ), Tübingen, Deutschland*, [4]*Universität Tübingen, Exzellenzcluster iFIT (EXC2180) „Individualisierung von Tumortherapien durch molekulare Bildgebung und funktionelle Identifizierung therapeutischer Zielstrukturen (iFIT)", , Tübingen, Deutschland*, [5]*Universität Tübingen, Interfakultäres Institut für Zellbiologie, Abteilung Immunologie, Tübingen, Deutschland*, [6]*Landeskrankenhaus Feldkirch, Allgemeine, Viszeral- und Thoraxchirurgie, Feldkirch, Österreich*, [7]*Schwarzwald-Baar-Klinikum Villingen-Schwenningen, Allgemeine und Viszeralchirurgie, Villingen-Schwenningen, Deutschland*

**Background:**
Laparoscopy is an essential element of staging locally advanced gastric cancer and indispensable for therapy planning. However, differentiation of tumor and scar tissue often requires biopsies. Hypericin, which is contained in St. John´s Wort, shows fluorescence as well as cytotoxic properties when excited by ultraviolet light. Since tumor cells accumulate hypericin, utilization of photodynamic diagnostics to guide biopsy in gastric cancer is promising.

**Materials and methods:**
Patients scheduled for staging laparoscopy with locally advanced gastric cancer were enrolled between 2017 and 2021. Single dose hypericin (Laif 900®; 900 mg) was orally administered two to four hours prior to surgery. Standard laparoscopy was extended by ultraviolet light-laparoscopy (390-440 nm) using the Storz D-Light-System. Presence and extent of suspect peritoneal lesions was assessed and samples taken. Photodynamic therapy over 15 minutes was added in case of suspicious abdominal manifestations. The trial is registered with EudraCT-Number 2015-005277-21 and clinical trials.gov-identifier NCT-02840331.

**Results:**
Until 2021, 50 patients were included in the trial, among whom 33 (66 %) were men. Mean age was 64.5 years. Standard and ultra-violet light-laparoscopy was generally feasible (100 %). There were no complications related to study medication or intervention. CT scans showed signs for peritoneal metastasis in eight patients (16 %), among whom the diagnosis was confirmed by laparoscopy-guided biopsy in six patients (75 %). Peritoneal metastases were suspected in 26 patients (52 %) during standard laparoscopy among whom diagnosis was attested by biopsy in 16 patients (64 %). Overall, in 25 patients (50 %) fluorescent areas were observed with ultraviolet light, which matched with histopathological diagnosis of peritoneal metastasis in 62 % of cases (16/25 patients).

**Conclusion:**
Hypericin-based fluorescence-enhanced laparoscopy in patients with gastric cancer is feasible and safe. However, this trial could not establish an added value of fluorescence-enhanced laparoscopy compared to the standard procedure. Whether there are means to improve the diagnostic value and to establish therapeutic relevance of the procedure remain to be determined.

**Estrogen alleviates acute kidney injury by regulating the expression of YB-1 via the PI3K/AKT pathway in a murine traumatic hemorrhagic shock model**
ID: 357

**Kategorie: DGCH - Chirurgische Innovation, Grundlagenforschung**

Kang Qin[1], Felix Bläsius[1], You LI[1], Weining Yan[1], Qun Zhao[1], Xing Zhang[1], Elizabeth Balmayor[1], Frank Hildebrand[1], Johannes Greven[1]

[1]*Department of Orthopaedics, Trauma, and Reconstructive Surgery, RWTH Aachen University Hospital, Aachen, Deutschland*

**Background:**
Traumatic hemorrhagic shock (THS) is a common cause of acute kidney injury (AKI), associated with high mortality and poor outcome. Estrogen (E2) plays a vital role in the protection of AKI. The Y box binding protein-1 (YB-1) and C-X-C Motif Chemokine 1 (CXCL1) were reported to be involved in kidney injury. This study aimed to determine whether estrogen can alleviate AKI by regulating the expression of YB-1 and CXCL1 in a murine traumatic hemorrhagic shock model.

**Materials and methods:**
Male C57/BL6 mice were randomly divided into four groups: sham, sham+E2, THS, and THS+E2. THS mice were exposed to arterial pressure-controlled (35-45 mmHg) hemorrhagic shock (HS) for 90 min and femoral fracture. Fluid resuscitation was performed after 90 min of HS, and estrogen or 0.9% saline (vehicle) was injected subcutaneously. Mice were sacrificed at 24 h after resuscitation. Kidney tissue was collected to perform H&E staining, immunohistochemical staining, western blot, Myeloperoxidase (MPO) test, and ELISA.

**Results:**
The immunohistochemical staining and western blot results demonstrated that E2 could increase renal YB-1 expression and consequently decrease CXCL1 and KIM-1 expression after THS. THS increased cytokine expression and lead to kidney injury, while E2 administration alleviated kidney injury and reduce cytokine expression. Furthermore, E2 administration decreased neutrophil infiltration, as shown by the decreased MPO levels. In addition, the western blot results showed that the PI3K/AKT pathway was activated by E2 after THS.

**Conclusion:**
E2 effectively increased the level of YB-1 and consequently reduced CXCL1 expression after THS, thereby alleviating AKI in mice.

**Tissue-instrument-interaction based palpation as a complementary tool for cartilage assement**
ID: 378

**Kategorie: DGCH - Chirurgische Innovation, Grundlagenforschung**

Thomas Sühn[1,2], Maximilian Costa[1], Nazila Esmaeili[2], Moritz Spiller[2], Axel Boese[2,3], Alfredo Illanes[2], Jessica Bertrand[4], Michael Friebe[3,5,6], Christoph Lohmann[1]

[1]*Orthopädische Universitätsklinik, Otto-von-Guericke-Universität, Medizinische Fakultät/Universitätsklinikum Magdeburg A. ö. R., Magdeburg, Deutschland*, [2]*SURAG Medical GmbH, Magdeburg, Deutschland*, [3]*INKA HealthTec Innovation Laboratory, Otto-von-Guericke-Universität, Medizinische Fakultät/Universitätsklinikum Magdeburg A. ö. R., Magdeburg, Deutschland*, [4]*Experimentelle Orthopädie, Otto-von-Guericke-Universität, Medizinische Fakultät/Universitätsklinikum Magdeburg A. ö. R., Magdeburg, Deutschland*, [5]*Department of Measurement and Electronics, AGH University of Science and Technology, Kraków, Poland*, [6]*CIB - Center of Innovation and Business Development, FOM University of Applied Sciences, Essen, Deutschland*

**Background:**
Osteoarthritis (OA) of the knee joint is a common disease among the elderly population typically leading to a partial or total knee replacement in later stages of progression. Beside other factors, the condition of the remaining cartilage is crucial for the decision whether a partial or total replacement is necessary. For clinical decision-making and preoperative planning, X-ray imaging and magnetic resonance imaging (MRI) are the undisputed gold standard to detect focal defects and diffuse cartilage loss [1]. While methods such as compositional MRI techniques and related biomarkers show promising results, they did not enter clinical practice yet [2]. In many cases the final decision for a partial or full replacement is therefore taken intraoperatively, based on the experience of the surgeon and the visual and haptic inspection of the exposed cartilage. Due to this, imaging technologies for an intraoperative assessment of the cartilage condition have entered the focus of research. Arthroscopy-based techniques such as optical coherence tomography (OCT) [3] and near-infrared spectroscopy (NIRS) [4] show promising results in pre-clinical studies. However, no commercial systems have entered the market yet and it is apparent that either modality will require a dedicated, costly hardware and will change the intraoperative workflow.
To circumvent expensive and complex imaging technology, Surgical Audio Guidance (SURAG) has been introduced recently as a novel sensing approach. It utilizes vibroacoustic waves originating from the interactions between a surgical instrument and the manipulated tissue. SURAG is attached to a standard instrument to capture the vibroacoustic waves that naturally propagate along the instrument structure and to extract information related to the interacting tissue or manipulation task [5,6]. The feasibility of this approach for a differentiation of surface textures using robotic laparoscopic instruments [7,8] and the potential to retrieve cartilage related information [9] has been shown already in preliminary experiments. Aim of this feasibility study is to prove the possible application of SURAG for the discrimination of mild degrees vs. highly degenerated osteoarthritic cartilage based on what we call a computerized palpation.

**Materials and methods:**
For the ethically approved study, ex-vivo tissue samples of osteoarthritic cartilage were collected from 43 patients undergoing a knee arthroplasty. The samples consisted of removed parts of the femoral condyles collected in the operating room and placed in phosphate-buffered saline (PBS) for further preparation. From those samples, multiple cartilage specimen with differing degree of osteoarthritic degeneration and a size of 10 x 6 mm were obtained using a punch. The specimen built the basis for the proposed computerized palpation and a subsequent histological analysis. The OARSI score [10] of each specimen was determined and used as the ground truth for the later labelling. In total 138 specimens were obtained ranging from OARSI score 0.5 (preserved cartilage) to 6.0 (subchondral bone, total loss of cartilage). Based on the OARSI score, the specimens were labelled into 3 classes considering the clinical motivation to support a partial or total knee replacement decision. The question here is: does the condition of the cartilage justify preservation of one femoral condyle, considering that a subsequent degeneration could cause an early revision surgery?
In this study, an OARSI score of ≤ 2.0 was defined as mild, still acceptable degree of degeneration and the related 67 specimens were labelled as class 1. Accordingly, the 54 specimens with an OARSI score between 2.5 – 5.0 were defined as highly degenerated cartilage and labelled as class 2. Class 3 consisted of 17 specimens with OARSI score of 6.0 which corresponds to a total loss of cartilage and thus plain subchondral bone.
For the computerized palpation using SURAG, a wireless, handheld vibration measurement system was mounted to an off-the-shelf orthopaedic instrument. The measurement system incorporates a mechano-acoustic sensor configuration to amplify and acquire vibro-acoustic signals directly from the surface at the proximal end of the instrument and stores them as audio files. A common palpation probe used in arthroscopic surgery (28124 BZ, KARL STORZ SE & Co. KG, Tuttlingen Germany) was used as basic instrument. The instrument was not modified and no integration of any active component was necessary. The measurements were performed within 1 hour after collecting the tissue samples by volunteering subjects experienced in experimentation and following a strict protocol. After removal from the PBS, each specimen was mounted in a holding frame and palpated 20 times by a minimum of 2 subjects each. A palpation was performed with a contact angle of ~45° between instrument tip and specimen surface in a defined direction over the full specimen length. In total 4320 palpations were performed for class 1 specimens, 3460 for class 2 and 1100 for class 3.
To check the capability of the acquired signals as basis for a discrimination of the classes, a total of 26 characteristic features were computed for each signal. The processing was based on the Continuous Wavelet Transformation (CWT) using a Morse mother wavelet. From the CWT-based time-frequency-representation, features related to the energy, the distribution of energy in the frequency bands and related to the time-variant dominant frequency were extracted. The obtained characteristics were used to train and compare 3 supervised classification algorithms: Decision Tree (DT), Linear Support Vector Machine (SVM) and k-Nearest Neighbours (kNN). Randomly 75% of the palpations of each subject for one specimen were assigned to the training dataset. The remaining 25% were assigned to the testing dataset. The training set was used for hyper-parameter optimisation using a 5-fold cross-validation with grid search method. To assess the performance of the created predictive models the testing set was applied and sensitivity and specificity values were calculated as measurement metrics.

**Results:**
The performances of all 3 classifiers concerning class 3 were very good, demonstrated by a sensitivity ≥ 92.4% and specificity ≥ 98.71%. The results confirm the assumption that a discrimination of subchondral bone based on the instrument-tissue-interaction should be possible due to its distinct surface and tissue characteristic compared to cartilage. It confirms previous results [7] and the general potential of SURAG for biologic tissue differentiation. The clinically relevant and more complex part is the discrimination of levels of cartilage degeneration. The difficulty reflects in the classifier's performance for the relevant class 1 of OARSI score ≤ 2.0. While the sensitivity shows promising results from 68,2% for the worst classifier DT to 76,8% for kNN and 79,0% for SVM as best performing classifier, specificity remains at low 67,7% for the classifier DT and drops even to ~63% for kNN and SVM. Although the classification results are not good enough yet for the targeted clinical application, to provide complementing information to support a treatment decision, the results are encouraging. It should be considered that the number of palpation samples was still little for the supervised learning-based classification and that a bigger dataset, an elimination of variances in the acquisition and the extraction of additional characteristic features certainly can improve the results. Even if today, it is difficult to identify a mild degeneration of cartilage, improving the accuracy in the future could avoid unnecessary treatments.

**Conclusion:**
The presented feasibility study demonstrates the potential of SURAG for the differentiation of osteoarthritic cartilage and its application in orthopaedic surgery. Moreover, the sensing approach could serve as a complementary tool or alternative to costly imaging technologies such as OCT or

NIRS. Future applications are conceivable regarding integration into robot-assisted arthroplasty or arthroscopy systems.

**References:**

[1] Alizai, Hamza et al. “Cartilage Imaging in Osteoarthritis.” *Seminars in musculoskeletal radiology* vol. 23,5 (2019): 569-578.
[2] MacKay, J W et al. “Systematic review and meta-analysis of the reliability and discriminative validity of cartilage compositional MRI in knee osteoarthritis.” *Osteoarthritis and cartilage* vol. 26,9 (2018): 1140-1152.
[3] O'Malley, Michael J, and Constance R Chu. “Arthroscopic optical coherence tomography in diagnosis of early arthritis.” *Minimally invasive surgery* vol. 2011 (2011): 671308.
[4] Sarin, Jaakko K., et al. "Near-Infrared Spectroscopy Enables Arthroscopic Histologic Grading of Human Knee Articular Cartilage." *Arthroscopy, Sports Medicine, and Rehabilitation* (2022).
[5] Illanes, Alfredo, et al. "Novel clinical device tracking and tissue event characterization using proximally placed audio signal acquisition and processing." *Scientific reports* 8.1 (2018): 1-11.
[6] Illanes, Alfredo, et al. "Surgical Audio Guidance SurAG: extracting non-invasively meaningful guidance information during minimally invasive procedures." *2019 IEEE 19th international conference on bioinformatics and bioengineering (BIBE)*. IEEE, 2019.
[7] Chen, Chien-Hsi, et al. "Texture differentiation using audio signal analysis with robotic interventional instruments." *Computers in biology and medicine* 112 (2019): 103370.
[8] Chen, Chien-Hsi, et al. "Proximally placed signal acquisition sensoric for robotic tissue tool interactions." *Current Directions in Biomedical Engineering* 4.1 (2018): 67-70.
[9] Sühn, Thomas, et al. "Acoustic sensing of tissue-tool interactions–potential applications in arthroscopic surgery." *Current Directions in Biomedical Engineering* 6.3 (2020): 595-598.
[10] Pritzker, Kenneth PH, et al. "Osteoarthritis cartilage histopathology: grading and staging." *Osteoarthritis and cartilage* 14.1 (2006): 13-29

**"C-C-Chemocine Receptor 4" is Crucial For Recruitment Of Tumor Associated Macrophages In Pancreatic Cancer And A Potent Target To Reduce Tumorsize and Prolong Survival in vivo**
ID: 522

**Kategorie: DGCH - Chirurgische Innovation, Grundlagenforschung**

Aydar Khabipov[1], Dung Nguyen Trung[1], Stephan Kersting[1], Sander Bekeschus[2], Lars - Ivo Partecke[3]

[1]*Universitätsmedizin Greifswald , Klinik für Allgemein- Gefäß- und Thoraxchirurgie , Greifswald, Deutschland*, [2]*Leibniz-Institut für Plasmaforschung und Technologie e.V., 17489, Deutschland*, [3]*Schleswig, Helios Klinik , Schleswig, Deutschland*

**Background:**
Pancreatic cancer is known for its immune-modulating properties. Inter alia, monocytes are recruited and polarized towards tumor-associated macrophages (TAM) via several chemokines. This promotes angiogenesis, metastasis, and tumor growth, causing high mortality and failure of conventional therapies in pancreatic cancer. New treatment options targeting TAM are urgently required. The C-C chemokine receptor type 4 (CCR4) plays a crucial role in the recruitment of immune cells into the tumor microenvironment. However, the impact of CCR4 receptor engagement on TAM and pancreatic cancer progression is less explored.

**Materials and methods:**
Murine PDA6606 pancreatic cancer cells and murine peritoneal macrophages were used for in vitro migration assays. In vivo, a syngeneic and orthotopic pancreatic cancer model was established. Tumor growth and survival were monitored under prophylactic and therapeutic application of a CCR4 antagonist (AF399/420/18025) in wild-type ($CCR4^{wt}$) and CCR4 knockout ($CCR4^{-/-}$) mice. TAM infiltration was monitored using microscopy of tumor tissue sections.

**Results:**
PDA6606 cells induced less migration in $CCR4^{-/-}$ than in $CCR4^{wt}$ in vitro. Pancreatic TAM infiltration was higher and survival was reduced in $CCR4^{wt}$ compared to $CCR4^{-/-}$ mice. Antagonizing CCR4 in wild-type mice revealed similar results as in $CCR4^{-/-}$ mice, whereby prophylactic CCR4 antagonist application was more efficient than therapeutic antagonization

**Conclusion:**
CCR4 seems to be critically involved in TAM generation and tumor progression in pancreatic cancer. CCR4 immunotherapy may help reduce pancreatic cancer malignancy and improve prognosis.

**iSurgeon: Augmented reality telestration for improved surgical training**
ID: 623

**Kategorie: DGCH - Chirurgische Innovation, Grundlagenforschung**

Eleni Felinska[1], Thomas Fuchs[1], Alexander Kogkas[2], George Mylonas[2], Beat Müller-Stich[1], Felix Nickel[1]

[1]*1Department of General, Visceral and Transplant Surgery, Heidelberg University Hospital , Heidelberg, Deutschland*, [2]*Hamlyn Centre for Robotic Surgery, Imperial College London, London, United Kingdom*

**Background:**
In laparoscopic surgery trainees need to learn how to interpret the operative field displayed on the laparoscopic screen. Experts currently guide trainees only verbally during laparoscopic surgical procedures. We developed the iSurgeon which allows the instructor to make hand gestures that are detected by a camera and displayed on the laparoscopic screen in augmented reality (AR) to provide visual expert guidance (telestration). Thus, the expert can provide clearer instructions by using gestures in addition to verbal instructions. This study analysed the effect of iSurgeon guided instructions on the gaze behaviour of instructor and trainee during laparoscopic surgery.

**Materials and methods:**
In a randomized-controlled cross-over trial, 40 laparoscopically naive medical students performed 7 basic laparoscopic tasks and one porcine laparoscopic cholecystectomy (task 8) with iSurgeon or with verbal instructions only. We used Pupil Core eye-tracking glasses to capture the instructor’s and the students’ gazes. Gaze behaviour was evaluated for tasks 1-7 by measuring the gaze latency, gaze convergence and collaborative gaze convergence. To measure the performance we counted the errors in tasks 1-7 and evaluated the trainees with structured and standardized performance scores in task 8 – the global and task specific objective structuctured assessment of technical skill (OSATS).

**Results:**
There was a significant difference found in tasks 1-7 for gaze latency ($p < 0.01$), gaze convergence ($p < 0.01$) and collaborative gaze convergence ($p < 0.01$). The error number was significantly lower in tasks 1-7 (0.18 ± 0.56 vs. 1.94 ± 1.80, $p < 0.01$) and the score ratings for task 8 were significantly higher with iSurgeon (global OSATS: 29 ± 2.5 vs. 25 ± 5.5, $p < 0.01$; task specific OSATS: 60 ± 3 vs. 50 ± 6, $p < 0.01$).

**Conclusion:**
AR based telestration with the iSurgeon successfully improved laparoscopic surgical performance. Trainee’s gaze behaviour was improved by reducing the time from instruction to fixation on targets and leading to a higher convergence of the instructor’s and the trainee’s gazes during the task. Also, the convergence of trainee’s gaze and target areas increased with the iSurgeon. This suggests that AR based telestration works by means of guiding the trainees’ gazes.

### The Role of the Brush Border Enzyme Intestinal Alkaline Phosphatase in Patients Undergoing Duodenectomy

ID: 648

**Kategorie: DGCH - Chirurgische Innovation, Grundlagenforschung**

Matthias Ilmer[1], Ruin Daifung[1], Viktor von Ehrlich-Treuenstätt[1], Sara Kakoschke[1], Josefine Schardey[1], Ulrich Wirth[1], Markus Albertsmeier[1], Bernhard Renz[1], Jan D'haese[1], Joachim Andrassy[1], Alexandr V. Bazhin[1], Jens Werner[1], Florian Kühn[1]

[1]*LMU Klinikum München, München, Deutschland*

**Background:**
The brush border enzyme intestinal alkaline phosphatase **(**IAP) prevents endotoxemia through detoxifying lipopolysaccharides (LPS), regulates gut barrier function, and maintains microbial homeostasis. While IAP deficiency has been linked to pathologies such as diabetes, metabolic syndrome, ischemic heart disease, frailty, and a decreased life span, supplementing the naturally occurring enzyme has been proven beneficial in several studies. As enterocytes predominantly secrete IAP in the duodenum, we hypothesized that pancreaticoduodenectomy (PD) leads to a significant decrease in this critical regulator of intestinal homeostasis.
Aim: To test if PD reduces IAP activity and a consecutive increase in LPS.

**Materials and methods:**
Enzymehistochemistry was performed to investigate intestinal IAP distribution. IAP levels were analyzed using the para-Nitrophenylphosphat method. Pre- and postoperative blood and stool samples were collected from patients undergoing PD. Stool samples were normalized by BCA protein assay. The Limulus amebocyte assay was used to measure serum LPS levels.

**Results:**
In total, 45 patients undergoing PD and 23 controls undergoing other major abdominal surgery (MAS) without duodenectomy were included in the study. Enzyme histochemistry confirmed that IAP was mainly present in the duodenum. PD significantly declined IAP levels compared to preoperative baseline levels ($p < 0.0001$). The decline in IAP levels correlated with the length of proximal small intestinal resection ($p=0.0034$). Compared to control patients, PD caused a much more pronounced reduction in IAP levels ($p<0.0001$) - also after adjusting for surgical trauma in terms of operative time and blood loss. Simultaneously, PD caused a more pronounced increase in serum LPS levels than other MAS ($p=0.0001$). Increased postoperative LPS levels were associated with an extended hospital stay ($p=0.0062$).

**Conclusion:**
Based upon the functional roles of IAP, supplementation with exogenous IAP might improve the short- and long-term outcome of patients undergoing pancreaticoduodenectomy.

**The effects of oncolytic vaccinia virus in human PDAC cell lines and on natural killer cell reactivity.**
ID: 878

**Kategorie: DGCH - Chirurgische Innovation, Grundlagenforschung**

Ruonan Wang[1], Zongsheng Guo[2], Mengwen Hu[1], Isis Lozzi Da Costa[1], Karl Herbert Hillebrandt[1], Lea Timmemann[1], Christian Benzing[1], Thomas Malinka[1], Igor Maximillian Sauer[1], Johann Pratschke[1], Matthäus Felsenstein[1]

[1]*Chirurgische Klinik, Charité – Universitätsmedizin Berlin, Berlin, Deutschland*, [2]*Roswell Park Cancer Institute, Dept of Immunology, Buffalo, United States*

**Background:** Pancreatic ductal adenocarcinoma (PDAC) is a dismal disease with markedly poor treatment response when immunomodulatory treatments were applied. Main challenges lie in the immune suppressed and non-inflamed tumor microenvironment with few effector cells targeting the carcinoma cells. Inoculation of oncolytic virus (OV) is a promising route for cancer treatment as specific tumor cell death is induced (oncolysis) leading to enhanced immunoreactivity via antigen cross-priming. We investigated the innate immune cell response in co-cultured human pancreatic carcinoma cell lines (Panc-1, BxPC3) following the infection with wildtype (vvDD) and armed vaccinia virus strain (vvDD-IL2/-IL15).

**Materials and methods:** Vaccinia virus armed with IL2 and IL15 (vvDD-IL2/IL15) and its parental virus vvDD were used to evaluate their antitumor effects. Effective tumor cell infection and oncolysis in PDAC cell lines (Panc1, BxPc3) were tested via MTS assay, FACS and immunofluorescence analyses. Following low-dose infection, effects on molecular signaling and viral protein expression in carcinoma cells were characterized by in-depth proteome and phosphoproteome analyses. In the PBMC co-culture setting we tested activation of natural-killer cells and effective tumor cell killing via FACS.

**Results:** We demonstrated that vvDD were replicating intracellular and lead to oncolysis in PDAC cell lines. Infection via armed vaccinia virus lead to effective transgene expression of IL2 and IL15. Proteomic signatures convey an induction of apoptosis pathways, reduced MHC-1 signaling and altered Inf-γ pathway regulation in PDAC carcinoma cells. We observed NK-cell exhaustion and minor activation in the co-culture setting after 24h and 48h. This however, did not lead to increased tumor cell killing. After infection with vvDD, vvDD-IL2 and vvDD-IL15 we observed increased activation in the Panc1-PBMC co-culture but not in BxPC-3-PBMC. Results correlated with increased tumor cell killing induced by NK-cells.

**Conclusion:** These findings underline the unresponsiveness of NK-cells towards PDAC cells. Oncolytic virus changes the intracellular molecular program in carcinoma cells and can improve NK-cell reactivity. Further investigation is needed to find better NK-cell targets and improve NK-cell reactivity in PDAC patients.

**Synergic crosstalk between intratumoral nerves and myeloid derived suppressor cells in pancreatic cancer via nerve-derived expression of CXCL8**
ID: 948

**Kategorie: DGCH - Chirurgische Innovation, Grundlagenforschung**

Kaan Cifcibasi[1], Carmen Mota Reyes[1], Teresa Zwick[1], Alexander Muckenhuber[2], Wilko Weichert[2], Helmut Friess[1], Hana Algül[3], Rouzanna Istvanffy[1], Ihsan Ekin Demir[1]

[1]*Klinikum rechts der Isar, Klinik und Poliklinik für Chirurgie, München, Deutschland*, [2]*Klinikum rechts der Isar, Institute of Pathology, München, Deutschland*, [3]*Klinikum rechts der Isar, Klinik und Poliklinik für Innere Medizin II, München, Deutschland*

**Background:**
Myeloid derived suppressor cells (MDSC) are known mediators of T-cell immunosuppression and correlate with poor clinical outcome in many solid tumors, including pancreatic cancer (PCa)[1]. In this study we aimed to uncover novel neuro-immune crosstalks in the pathogenesis of neural invasion in PCa.

**Materials and methods:**
MDSC were characterized within the tumor microenvironment (TME) and the perineural niche of PCa patients (n=40) using double immunofluorescence. In vitro migration assays and secretome arrays were used for the functional analysis of the invasive potential of pancreatic tumor cells towards DRG-neurons in the presence of MDSC. Spatial transcriptomic technology (NanoString) allowed us to determine the differential gene expression on nerve-invading tumor cells as well as on tumor-invaded nerves.

**Results:**
PCa patients with severe NI presented increased density of intratumoral MDSC. Furthermore, increased MDSC-infiltration was shown in the perineural niche of tumor-invaded nerves compared to non-invaded ones. Mechanistically, the migratory behaviour of PCa cells towards neurons was significantly enhanced by the interaction with MDSCs and it led to increased MDSC-proliferation and significantly higher secretion of CXCL8 and CCL5. The expression of CXCL8, a known chemoattractant of MDSCs, was notably higher on nerves invaded by tumor cells, whereas its expression on tumor cells remained constant. Furthermore, enrichment analysis of bulk RNAseq from pancreatic tumors with severe NI demonstrated increased chemokine activity and CXCR-chemokine receptor binding, which are typically expressed on myeloid immune cells.

**Conclusion:**
CXCL8 derived from intrapancreatic nerves contributes to chemoactive recruitment and proliferation of MDSC in the TME which highlights the critical role of nerves in modulating the tumor immune profile PCa. The discovery of a neuro-immune synergic crosstalk between intrapancreatic nerves and MDSCs through CXCL8 is an actionable strategy to reduce neural invasion in pancreatic cancer.

1. Zhang J, Xu X, Shi M, Chen Y, Yu D, Zhao C, Gu Y, Yang B, Guo S, Ding G, Jin G, Wu CL, Zhu M. CD13hi Neutrophil-like myeloid-derived suppressor cells exert immune suppression through Arginase 1 expression in pancreatic ductal adenocarcinoma. Oncoimmunology. 2017 Jan 9;6(2):e1258504. doi: 10.1080/2162402X.2016.1258504. PMID: 28344866; PMCID: PMC5353902.

**Nerves as Mediators of Immune Suppression in Pancreatic Ductal Adenocarcinoma**
ID: 959

**Kategorie: DGCH - Chirurgische Innovation, Grundlagenforschung**

Kaan Cifcibasi[1], Carmen Mota Reyes[1], Rouzanna Istvanffy[1], Ihsan Ekin Demir[1]

[1]*Klinikum rechts der Isar, Klinik und Poliklinik für Chirurgie, München, Deutschland*

**Background:**
Pancreatic ductal adenocarcinoma (PDAC) is characterized by pronounced intratumoral nerve growth, neural invasion and neuro-inflammation. In our preliminary analysis, we demonstrated mutual trophic effects between myeloid derived suppressor cells (MDSCs) and dorsal root ganglia (DRG) neurons. Furthermore, increased perineural accumulation of MDSCs positively correlates with neural invasion in human PDAC. The pronounced neural growth, spread and growth of cancer cells with epithelial features along nerves, the remarkable "neurogenesis" in cancer and the herein identified enrichment of immunosuppressive immune cells like MDSCs suggest a mechanism of nerve-mediated immunosuppression in cancer. Therefore, to elucidate the role of neuron-mediated immune suppression in pancreatic cancer, we are characterizing the transcriptional profile of DRG neurons innervating tumor bearing mouse pancreata using spatial transcriptomics, and cross-validating our in vivo findings with our intraoperative PDAC patient biopsy samples and further in vitro experiments.

**Materials and methods:**
DRG isolation and FFPE block preparation will be performed from genetically engineered (KC, KPC, and our neuroinvasive TPAC mouse model) and surgically induced (orthotopic implantation and electroporation models) mouse models of PDAC. The DRG neurons will be subject to spatial transcriptomic profiling of neurons located in DRGs. The identified targets will be subject to histopathological analysis and validation of the targets found to be overexpressed in mouse DRG and tumor samples. We will also perform cross-validation of overexpressed targets in human PDAC biopsy samples. In addition, we will analyse the immunosuppressive capacity of DRGs through in vitro immune suppression assays. Finally, we will validate the immunomodulatory capacity of DRGs by inhibition/ ablation of DRG neurons.

**Results:**
Data analysis of spatial transcriptomics of DRGs is currently ongoing. Results will follow shortly.

**Conclusion:**
Our spatial transcriptomic analyses of DRGs in different mouse models of PDAC is going to create a broad perspective on the diverse roles neurons can play in peripheral cancers. This new perspective has the potential to lead us to novel therapeutic targets on the neuroimmune axis to improve the abysmal prognosis of PDAC patients.

**Morbidity and Mortality of Neutropenic Patients in Visceral Surgery**
ID: 85

**Kategorie: DGCH - Chirurgische Konzepte bei seltenen Erkrankungen**

Ann-Kathrin Lederer[1], Fabian Bartsch[1], Markus Möhler[2], Peter Gaßmann[1], Hauke Lang[1]

[1]*Universitätsmedizin der Johannes Gutenberg-Universität Mainz, Klinik für Allgemein-, Viszeral- und Transplantationschirurgie, Mainz, Deutschland*, [2]*Universitätsmedizin der Johannes Gutenberg-Universität Mainz, I. Medizinische Klinik und Poliklinik, Mainz, Deutschland*

**Background:**
Leukocytes are essential for the function of the immune system and cell-cell-interaction in the human body, but hematological diseases as well as chemotherapeutic treatments due to cancer lead to occasionally or even permanent leukocyte deficiency. Leukopenia is mostly characterized by a decrease of neutrophilic granulocytes. The consequence of neutropenia is susceptibility to infection, but also healing disorders are suggestable due to the disturbed cell-cell-interaction. In general, surgery is obviated in neutropenic patients, whenever possible, but patients, who suffer from neutropenia, are sometimes in urgent need of surgery. Less is known about the morbidity and mortality of these patients, which is why this review aimed to critically summarize results of recent research in this particular field.

**Materials and methods:**
We performed a literature review searching databases of Medline and Web of Science from inception to August 2022. All kind of human studies except of case reports and case series were eligible for evaluation. All adult patients regardless of gender and all surgical procedures of the gastrointestinal tract were considered. Eligible studies had to report about preoperative leukopenia/neutropenia and outcome after visceral surgery.
The following research questions were posed before starting database research:
Does preoperative neutropenia affect the morbidity and mortality of patients undergoing emergency visceral surgery?
Does preoperative neutropenia affect the morbidity and mortality of patients undergoing elective visceral surgery?

**Results:**
Overall, 1147 results were evaluated and 17 reports were included for final analysis (Figure 1).
**Figure 1.** Flow-chart of inclusion.

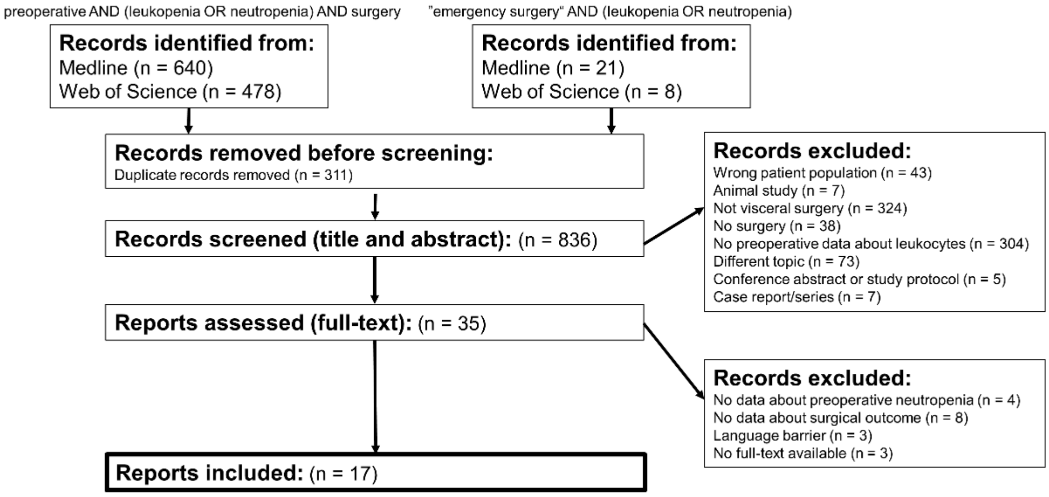

Even if the mortality rate of neutropenic patients undergoing emergency surgery might be higher compared to non-neutropenic patients, surgery might be the only approach for survival in case of impending abdominal sepsis. Similar to other critical-ill patients, it appears that prompt diagnosis followed by an early and specific treatment is essential for the outcome of neutropenic patients.The results emphasize the necessity of being highly attentive as symptoms and disease patterns of neutropenic patients might be different to immunocompetent patients. If in doubt, a surgical colleague should be consulted to evaluate abdominal pain in a neutropenic patient, and rapid radiologic diagnostic should be performed.
In elective surgery, it is not suggested by the results that neutropenic patients are at high risk for postoperative complications or even postoperative death. However, data about detailed analysis of development of postoperative complications in neutropenic patients are widely lacking.
**Table 1.** Overview of all included reports.

| Author | Ref. | Year | Study type | n* | Type of surgery | Disease | Results° |
|---|---|---|---|---|---|---|---|
| Wade et al. | [21] | 1990 | Retrospective | 17 | Emergency | Hematologic | Mortality 41% |
| Nishida et al. | [24] | 1996 | Pilot trial | 8/23 | Emergency | Perforation | Mortality 13% vs. 65%$^{+}$ |
| Hansen et al. | [25] | 1998 | Retrospective | 105 | Emergency | Diverticulitis | Predictor for death |
| Sullivan et al. | [26] | 2012 | Retrospective | 956/956 | Emergency | Diverse | Predictor for death |
| Sudarshan et al. | [27] | 2015 | Retrospective | 527 | Emergency | Diverse | Predictor for death & complications |
| Gulack et al. | [28] | 2015 | Retrospective | 2057/ 18386 | Emergency | Diverse | Predictor for death |
| Mokart et al. | [29] | 2017 | Retrospective | 17/58 | Emergency | Hematologic | No association |
| Joo et al. | [30] | 2020 | Retrospective | 12/79 | Emergency | Colonic perforation | Predictor for death |
| Fokstuen et al. | [31] | 2009 | Retrospective | 274 | Elective | Rectal cancer | No association |
| Reim et al. | [32] | 2010 | Retrospective | 58/156 | Elective | Gastric cancer | No association |
| Bamba et al. | [33] | 2014 | Retrospective | 33/66 | Elective | Diverse | Association to an early port infection |
| Ohira et al. | [34] | 2015 | Retrospective | 44 | Elective | Esophageal cancer | No association |
| Pluta et al. | [35] | 2018 | Retrospective | 101 | Elective | Diverse | No association |
| Chen et al. | [36] | 2019 | Retrospective | 141 | Elective | Liver metastases | Predictor for major complications |
| Grant et al. | [37] | 2020 | Retrospective | 891/ 3493 | Elective | Malignant disease | No association |
| Hara et al. | [38] | 2021 | Retrospective | 52/100 | Elective | Esophageal cancer | No association |
| Zarain-Obrador et al. | [39] | 2021 | Retrospective | 1727 | Elective | Colorectal surgery | Risk factor for surgical site infection |

*In case of controlled study, n is reported as followed: n study group/n control group
°Outcome of leukopenic/neutropenic patients or impact of preoperative leukopenia/neutropenia on morbidity and mortality
+All patients were neutropenic, but study group received G-CSF perioperatively; Ref = Reference, ICU = Intensiv Care Unit

**Conclusion:**
The results emphasize the role of neutrophilic granulocytes in inflammation and immunity in surgical patients, but it appears to be extremely difficult to assess the isolated effect of neutropenia on postoperative morbidity and mortality of visceral surgery patients. Most of the evaluated studies showed methodological flaws due to small sample-sizes or risk of bias. Nevertheless, the results are interesting and emphasize that physicians have to be reckon with atypical clinical patterns obscuring a potential life-threatening severe affection that requires urgent therapy. The results of this review suggest that neutropenic patients in need of emergency surgery have a higher mortality risk compared to non-neutropenic patients. In contrast, in elective surgery there was not a clear tendency for a higher mortality risk of neutropenic patients, but future research should evaluate the risk for postoperative complications, particularly of infectious complications such as surgical site infections.

**Internal Herniation through the Foramen of Winslow**
ID: 324

**Kategorie: DGCH - Chirurgische Konzepte bei seltenen Erkrankungen**

Sarah Öfele[1], Stefan Rüth[1]

[1]*Asklepios Stadtklinik Bad Tölz, Allgemein- und Viszeralchirurgie, Bad Tölz, Deutschland*

**Background:**
Foramen of Winslow hernias are rare events accounting for only 8 % of internal hernias (Harnsberger et al. 2015; Evrard et al. 1996). They are associated with nonspecific symptoms and delayed diagnosis that can lead to a mortality rate up to 49 % (Osvaldt et al 2008). In most cases the herniation comprises small bowel, followed by coecum or ascending colon (Sikiminywa-Kambale et al. 2014; Puig et al. 2013). The patients present with acute abdominal pain related to the associated bowel obstruction (Cohen et al. 1982). Treatment requires surgical intervention with reduction of the hernia, thorough inspection of the bowel and, if necessary, resection of ischemic segments (Martin et al. 2006; Osvaldt et al. 2008). Due to the rarity of this condition, there are no established surgical algorithm to prevent recurrence of herniation. Prophylactic measurements often discussed are closing the foramen of Winslow via stiches or mobilized omentum and fixation or resection of the excessively mobilized viscera (Moris et al. 2018, Ayoob et al. 2019)

**Materials and methods:**
A 50-year-old male patient presented to the emergency department with severe abdominal pain that started approximately 12 h before presentation. It was associated with nausea and one episode of non-bloody vomiting. He had no medical or surgical history. There was no change in bowel habits. Diagnosis was made through computer tomography followed by explorative laparotomy.

**Results:**
Physical examination was nonspecific with tenderness in the epigastrium and mild abdominal distension. Laboratory studies revealed elevated leukocytes (14,3x103 /µl) and lactate (26,4 mg/dl). A computer tomography scan of the abdomen and pelvis with intravenous contrast was performed and showed gastroparesis and an obstruction of the distal small bowel located in the omental bursa. Emergent surgery revealed herniation of the small intestine through the foramen of Winslow. The intestine loop could not be reduced via laparoscopic approach so laparotomy was performed. After reduction the small bowel was seen to be viable and well perfused. To prevent recurrence, constricting strands of the ligamentum hepatoduodenale were resected and the foramen of Winslow was covered with omentum. The postoperative recovery was uneventful and the patient was discharged on postoperative day four.

**Conclusion:**
Early diagnosis and treatment of foramen of Winslow hernias are essential to prevent severe complication as gangrenous necrosis and bowel perforation (Puig et al 2013). Due to the rarity of this condition, there are only limited data available. Further studies are needed to evaluate and compare the benefits of different prophylactic measures to avoid recurrence. Additionally, more data need to be collected to identify risk factors of this rare type of internal hernia.

### The Cyst of canal of Nuck – a very rare diagnosis in adult women

ID: 558

**Kategorie: DGCH - Chirurgische Konzepte bei seltenen Erkrankungen**

Michael Kohlhauser[1], Julian Pirsch[1], Thorsten Maier[2], Christian Viertler[3], Roland Fegerl[1]

[1]*Landeskrankenhaus Weiz, Weiz, Österreich*, [2]*Institut für CT und MRT Weiz, Weiz, Österreich*, [3]*Diagnostik & Forschungsinstitut für Pathologie, LKH-Universitätsklinikum Graz, Graz, Österreich*

**Background:**
In 1691, Anton Nuck, a Dutch anatomist, was the first to describe the canal of Nuck. The canal of Nuck is the female equivalent to the processus vaginalis in males, which usually disappears within the first year of life. It consists of an evagination of peritoneum, which is attached to the uterus by the round ligament, and proceeds through the inguinal ring alongside the round ligament into the labia majora. Usually, the superior part of this outpouch obturates during or just before birth and disappears within the first year of life. In rare cases, this obturation fails, resulting in a persistence of the canal of, which can cause the formation of a female hydrocele, namely the cyst of the canal of Nuck. The cyst of the canal of Nuck is an extremely rare entity, usually occurring in children, but also in adult women. This phenomenon in women was first reported by Coley in 1892. Clinically, a female hydrocele typically manifests as a swelling in the groin or genital region, which allows for a variety of differential diagnoses. Due to its rarity and the lack of awareness among health professionals, the cyst of the canal of Nuck is often misdiagnosed. Precise diagnosis, including a thorough clinical examination and adequate radiological imaging, is required to accurately determine its presence.

**Materials and methods:**
I report on a case of cyst of the canal of Nuck in a 42-year-old woman, who presented with a painful swelling at her right groin. In addition, I give an overview of the anatomy, pathogenesis, classification, diagnostics, differential diagnosis and treatment of the cyst of the canal of Nuck, according to the available literature in order to highlight best practice in medical care of this phenomenon. The following databases were used to search for and identify the included literature: PubMed, Google Scholar, and MEDLINE. This overview should serve to facilitate the diagnostic of female hydroceles and their treatment.

**Results:**
Through interdisciplinary cooperation, the correct diagnosis was made in our case and the patient was treated adequately. The histological intervention confirmed the diagnosis of cyst of the canal of Nuck. In our follow-up 6 months after surgery, the patient was asymptomatic and satisfied with the treatment.

**Conclusion:**
Due to the rare clinical occurrence and the lack of literature, a diagnosis of a cyst of the canal of Nuck is often difficult to make, not only for inexperienced surgeons, but also for medical experts. Thus, interdisciplinary collaboration in healthcare between various different fields, such as radiology and surgery, is necessary to prevent misdiagnosis as well as resultant errors in treatment. A focused physical examination followed by high-resolution sonography enables the diagnosis of a cyst of the canal of Nuck. To plan an adequate surgical intervention, cross-sectional imaging, preferably MRI, allowing clarification of the anatomical conditions is of utmost importance. With this report we would like to raise awareness of the possibility of a female hydrocele, which is of utmost importance.

**Transversus Abdominis Muscle Release (TAR) in Giant Incisional Hernia**
ID: 760

**Kategorie: DGCH - Chirurgische Konzepte bei seltenen Erkrankungen**

Baco Stanko[1], Miloš Mitrić[2]

[1]*General surgery, Public Health Institution Hospital “Dr Mladen Stojanovic”,, Prijedor, Bosnia and Herzegovina*, [2]*Public Health Institution Hospital “Dr Mladen Stojanovic”,, Prijedor, Bosnia and Herzegovina*

**Background:**
Transversus abdominis muscle release (TAR) is a new myofascial release technique that involves the creation of a retro rectal place and mesh placement. It is a modification of the posterior component separation technique (CST) and enables the primary closure of the most challenging abdominal wall reconstructions.
We present a case,of giant ventral incisional hernia, where a favorable outcome was achieved with Transversus abdominis muscle release technique (TAR), in a contaminated environment with almost no perioperative complications and no recurrence after 20 months, with the placement of inexpensive non-absorbable ‘Paha’ mesh.

**Materials and methods:**
A 70-year-old man, who had been twice operated on in our hospital, presented with a giant ventral incisional hernia for an elective operation.
He had undergone an emergency laparotomy for the first time due to an adhesional ileus, with adhesiolysis of the small intestine and colonis sygmoidei adhesions. The postoperative course had been complicated by a medial laparotomy wound dehiscence and evisceration of the small intestine on the 12th postoperative day. After the reoperation with wound revision and closure, a surgical site infection developed, which led to the development of a giant incisional hernia.
At the time of examination, his general condition was good and a ventral hernia, arrising about 80 x 40 cm from the anterior abdominal wall was present. Regarding comorbidities, he had experienced a chronic hepatitis B infection.
The rectus defect in the umbilical region in the transverse direction was 19.9 cm wide and 20 cm in the vertical direction. CT of the abdomen showed a defect area of 398 $cm^2$ and the bowel adherent to the skin. Due to the local findings, we were compelled to resect approximately 15 cm of the intestine tenui and constructed a T-T ileo-ileal anastomosis. The peritoneum and the fascia transversalis were approximated in the midline using absorbable Vicryl 2/0 sutures (some peritoneum fenestrations were repaired). Two non-absorbable, monofilament polypropylene mesh sized 30 x 30 cm were placed retro muscular (sublay). The anterior rectus sheath could not be approximated. Panniculectomy and scar excision followed. The patient was shifted after two hours to the general surgery department with intravenous analgesia. The patient was discharged on the $11^{th}$. A minor postoperative complication was a wound seroma, which spontaneously resolved.

**Results:**
The classic open anterior CST was first reported by Albanese and popularized by Ramirez decades later. It involves the functional reconstruction of the abdominal wall with autologous tissue repair. Wide skin flaps are created to access the external oblique aponeurosis. The external oblique aponeurosis is divided longitudinally lateral to the rectus compartment and separated from the underlying internal oblique muscle. The myofascial flap consisting of the rectus, internal oblique, and transversus abdominis muscle (TAM) is mobilized medially. Because of high complication rates, many new techniques have been developed.
Milburn et al. (2007, cadaver trial) and Carbonell et al. (2008) reported a posterior CST (PCST) technique. It involves the incision of the PRS, after the separation from the rectus muscle, at its most lateral edge with access to the plane between the internal oblique and TAM. The fact that the plane between the internal oblique and TAM contains nerves and blood vessels leads to the main disadvantage of this technique - cutting of the intercostal nerves while advancing from the rectus abdominis to the lateral compartment, which may result in partial or complete rectus muscle paralysis. The problem of extending the dissection from the rectus abdominis muscle to the lateral compartment was resolved by Novitsky et al., who reported TAR as a new technique: a modification of the PCST and an extension of the Rives-Stoppa-Wantz. It represents one of the major advances in abdominal wall surgery in the last decades and is indicated for very large hernias or combined abdominal wall defects. Mesh is placed sublay; retro muscular and perforators to the rectus muscle are identified and preserved (longitudinal incision of the PRS 1.5-2 cm medial to the lateral border of the rectus compartment). It offers superior results over non-mesh reinforced repairs. TAR could be an ideal hernia repair technique for complex defects because it weakens the lateral sides of the abdominal wall to augment the midline repair, thereby also increasing the lateral abdominal wall compliance and reducing the pressure on the midline. However, TAR should not be combined with anterior CST.

**Conclusion:**
Our case shows that even the most challenging abdominal wall hernias can be reconstructed with TAR. It is a safe, effective, and reliable technique with low perioperative morbidity as well as reduced risk of skin necrosis and surgical site infection. Recurrence rates, however, are yet to be analyzed. Additionally, the successful use of synthetic mesh reinforcement (in the most suitable retro muscular sublay plane), even in a contaminated environment, is possible, leading to a favorable outcome.

**Nutritional status is not affected by multivisceral resections and resections of recurrent disease in patients with retroperitonal soft tissue sarcoma**
ID: 929

**Kategorie: DGCH - Chirurgische Konzepte bei seltenen Erkrankungen**

Franziska Willis[1], Anna-Marlen Trunk[1], Martin Schneider[1]

[1]*Heidelberg, Universitätsklinikum Heidelberg, Heidelberg, Deutschland*

**Background:**
Retroperitoneal soft tissue sarcoma (RPS) are rare and heterogeneous tumors and account for 1 % of malignancies in adults. Surgery is the mainstay in RPS treatment since, besides tumor biology such as grading and histological subtype, macroscopic complete resection is the most important prognostic factor for overall survival. To minimize the probability of tumor-infiltrated resection margins, compartmental resection has become a standard technique for RPS resection. This approach includes resection of all organs and structures adjacent to the tumor which is why multivisceral resection is often necessary. Nonetheless, local recurrences and distant metastases are frequent in RPS and constitute a major problem in clinical management. It has been shown that repeated surgery for RPS recurrence is associated with improved overall patient survival. However, the impact of (multiple) often multivisceral resections on patients' nutritional status has not been investigated yet. It is known that cancer patients with reduced nutritional status have worse oncological outcomes. To investigate to what extent the nutritional status is influenced by repeated surgery in RPS and to identify possible areas of improvement regarding perioperative care in terms of (p)rehabilitation were the aims of this study.

**Materials and methods:**
Patients undergoing resection of primary and recurrent RPS ant the University of Heidelberg Department of General, Visceral and Transplantation Surgery were retrospectively analyzed. To assess the nutritional status the body mass index (BMI), the prognostic nutrition index (PNI), the geriatric nutritional risk index (GNRI), and the modified Glasgow prognostic score (mGPS) for cancer outcomes were calculated. The impact of nutritional status on overall and local recurrence free survival was analyzed via Kaplan-Meyer analyses. Hierachical linear models (HLM) were used to analyze nutritional status during the course of the disease and to identify possible predictors for reduced nutritional status, multivisceral resection, resection of specific organs such as the pancreas as well as the presence of postoperative complications were introduced as two-way interaction terms.

**Results:**
332 patients were included in this study. Overall nutritional status was very well in our cohort: At first presentation more than 90% of our patients showed no risk for nutritional deficiencies regarding to the PNI and GNRI. 88 % of all patients of whom assessment of mGPS was possible, fulfilled criteria for the "good prognosis" group. Better scores according the GNRI and mGPS at first presentation were significantly associated with improved overall survival in Kaplan-Meyer analyses ($p = 0.004$ and $p < 0.001$). HLM analyses showed that nutritional parameters were stable during the course of the disease (all $p > 0.5$). Neither the histological subtype, nor the performance of a multivisceral resection or the presence of postoperative complications had an impact on nutritional status. Only (partial) resection of the pancreas was associated with worse mGPS scores.

**Conclusion:**
Multivisceral and repeated resections are common during the course of the disease of patients with RPS. Nutritional status was not significantly affected by multiple operations, corroborating existing findings that multiple operations for recurrent disease are acceptable in terms of postoperative morbidity and quality of life. However, (partial) resection of the pancreas was associated with a worse postoperative prognosis due to reduced nutritional status, indicating that special focus should lie on this patient subgroup during postoperative follow-up. Moreover, reduced nutritional status before first operation was associated with reduced overall survival corroborating the need of preoperative screening of nutritional status and offering prehabilitation programs if necessary.

**Primary anastomosis after gastrointestinal perforations in patients with co-existing hematological malignancies is associated with high anastomotic leakage rate and 30-day mortality**
ID: 137

**Kategorie: DGCH - Chirurgische Notfall- und Intensivmedizin**

Philipp Holger von Kroge[1], Anna Duprée[1], Oliver Mann[1], Jakob Izbicki[1], Jonas Wagner[1], Paymon Ahmadi[2], Sören Weidemann[3], Nicolaus Kröger[2], Carsten Bokemeyer[2], Walter Fiedler[2], Franziska Modemann[2], Susanne Ghandili[2]

[1]*Universitätsklinikum Hamburg-Eppendorf, Klinik für Allgemein-, Viszeral- und Thoraxchirurgie, Hamburg, Deutschland*, [2]*Universitätsklinikum Hamburg-Eppendorf, Klinik für Onkologie, Hämatologie und Knochenmarktransplantation mit der Sektion Pneumologie, Hamburg, Deutschland*, [3]*Universitätsklinikum Hamburg-Eppendorf, Institut für Pathologie mit den Sektionen Molekularpathologie und Zytopathologie, Hamburg, Deutschland*

**Background:**
Hematologic patients requiring abdominal emergency surgery are considered to be a high-risk population based on disease- and treatment-related immunosuppression. However, the optimal surgical therapy and perioperative management of patients with abdominal emergency surgery in patients with co-existing hematological malignancies remain unclear. The aim of this study was to investigate the outcome of patients with hematological malignancies undergoing abdominal emergency surgery for gastrointestinal perforation.

**Materials and methods:**
All patients included in this retrospective single-center study were treated at the University Medical Center Hamburg-Eppendorf between January 2010 and May 2022. They were identified by screening for the ICD 10 diagnostic codes for gastrointestinal perforation (K63.1, K63., K25. and K31.). In addition, a keyword search was performed in the database of all pathology reports in the given time frame. In particular, the keywords gastrointestinal, esophagus, stomach, small intestine, colon, and rectum in combination with lymphoma were used.
The primary endpoint of the study was to investigate the surgery-related 30-days and 90-day mortality. Secondary endpoints were surgical complications defined as anastomotic leakage, fecal peritonitis, wound healing disorders, intraabdominal abscesses, and bleeding events.

**Results:**
A total of 26 patients were included. Of those, 23 had been diagnosed with B-cell non-Hodgkin lymphoma, mainly diffuse large B-cell lymphoma and Burkitt-lymphoma. Two patients had been diagnosed with plasma-cell disorders, and one patient with myeloid neoplasia. 69.2 % had enteral or mesenteric involvement of the underlying hematological disorder.
The most frequent localization of perforation was small bowel (58%) followed by colon and stomach (each 15%). 39% of the perforations were related to hematological disorder and 31% were interpreted as treatment-related perforations. In the majority of patients (18/26), gastrointestinal perforation occurred prior to systemic treatment, during steroid pre-phase, or during the first therapy cycle.
A total of 17 patients received a primary anastomosis, whereas in eight patients a primary stomy was created. The overall 30-day and 90-day mortality were 19.2% and 34.6%, respectively. Six patients (35.3%) developed anastomotic leakage resulting in several reoperations (range from 3 to 15) due to fecal peritonitis. The anastomotic leakage-related 30-day mortality was 80%. In general, none-anastomotic leakage-related causes of death were pneumogenic septic shock and cancer progression. The 30-day mortality was significantly higher in patients with anastomotic leakage compared to those without anastomotic leakage ($p = 0.006$).

**Conclusion:**
We observed a high 30-day and 90-day mortality in patients with gastrointestinal perforation and co-existing hematological malignancies. Based on our results, we suggest that in patients with known or suspected hematologic malignancies who require emergency abdominal surgery due to gastrointestinal perforation, a temporary or permanent stoma should be preferred to a primary intestinal anastomosis.

**Management of acute appendicitis during COVID-19 pandemic. Single center data from a tertiary care hospital in Germany.**
ID: 797

**Kategorie: DGCH - Chirurgische Notfall- und Intensivmedizin**

Mihailo Andric[1], Michael Klös[1], Roland S. Croner[1], Aristotelis Perrakis[2]

[1]*Universitätsklinik für Allgemein-, Viszeral-, Gefäß- und Transplantationschirurgie Otto-von-Guericke-Universität Magdeburg, Magdeburg, Deutschland*, [2]*Uniklinikum Magdeburg, Universitätsklinik für Allgemein-, Viszeral-, Gefäß- und Transplantationschirurgie , Magdeburg, Deutschland*

**Background:**
The unexpected global overload of the health system during COVID-19 pandemic has caused changes in management of acute appendicitis worldwide. Whereas conservative treatment was widely recommended, the appendicectomy remained gold-standard therapy in Germany. We aimed to investigate the impact of COVID-19 pandemic on treatment routine for acute appendicitis at University Hospital of Magdeburg.

**Materials and methods:**
Adult patients with clinical and/or radiological diagnosis of acute appendicitis were included in the single center retrospective study. Data was collected to patient demographics, treatment modality and outcomes including morbidity and length of stay. The patient data related to COVID-19 period from March 22, 2020 to December 31, 2021 (649 days) were compared to the Non-COVID-19 period from June 12, 2018 to March 21, 2020 (649 days). Subgroup analysis related to conservative or surgical treatment has been performed.

**Results:**
A total of 385 patients was included in the study, 203 (52.73%) during Non-COVID-19 period and 182 (47.27%) during COVID-19 period. Mean age of entire collective was 43.28 years, containing 43.9% female patients (p=0.095). Conservative treatment was accomplished in 49 patients (12.7% of entire collective), increased from 9.9% to 15.9% during COVID-19 period (p=0.074). Laparoscopic appendicectomy was performed in 99.3% (n=152) of operated patients during COVID-19 period (p=0.013), followed by less postoperative complications compared to reference period (23.5% vs 13.1%, p=0.015). The initiation of antibiotic therapy after the diagnosis increased from 37.9% to 53.3% (p=0.002) regardless the following treatment modality but lasted shorter during pandemic period (5.57 days vs. 3.16 days, p< 0.001) and was longer in the conservative treatment group (5.63 days vs. 4.26 days, p=0.02).
The overall length of stay was shorter during COVID-19 period (4.12 days vs. 4.67 days, p= 0.052) and in the conservative treatment group (3.08 days vs 4.47 days, p< 0.001). However, the overall morbidity was lower during the COVID-19 period (17.2% vs. 7.7%, p=0.005) and for the conservative therapy (14.3% vs. 2%, p=0.016). There was no mortality documented.

**Conclusion:**
According to our findings the COVID-19 pandemic had a relevant impact on management of acute appendicitis. Although laparoscopic appendicectomy remains the recommended procedure, the conservative treatment of uncomplicated appendicitis with excellent short-term outcome can be a safe alternative to surgery during potential new wave of COVID-19 pandemic and in the daily routine.

**Pre- and intraoperative risk factors associated with the need for intensive care treatment in adult patients with acute small bowel obstruction caused by adhesive bands**
ID: 845

**Kategorie: DGCH - Chirurgische Notfall- und Intensivmedizin**

Chris Paul Günther[1], Jens Strohäker[1], Ruth Ladurner[1], Anke Meier[2]

[1]*Universitätsklinik für Allgemeine, Viszeral- und Transplantationschirurgie, Tübingen, Deutschland*,
[2]*Universitätsklinik für Allgemeine, Viszeral- und Transplantationschirurgie, Tübingen, Deutschland*

**Background:**
Acute mechanical small bowel obstruction (SBO) caused by adhesive bands is a frequent surgical emergency requiring quick clinical evaluation and often leads to immediate surgical treatment. Currently, the indication for postoperative observation in a surgical intensive care unit (SICU) is a clinical pre-, intra-, or postoperative interdisciplinary decision. Since general criteria for postoperative ICU admission still underly a lack of high-quality evidence [1], the aim of our study is to identify reliable pre- and intraoperative indicators determining the need for postoperative observation and treatment in a SICU to support clinical decision making.

**Materials and methods:**
94 adult patients who have been operated at our tertiary hospital with severe SBO caused by adhesive bands between January 2016 and January 2021 were studied retrospectively. Patients without severe SBO and children were excluded. Research was realized in the digital clinical database. Basic demographic data, laboratory evaluations, operation reports, anesthesia data, medication, pre-existing diseases, and radiologic imaging data were analyzed.

**Results:**
The mean age of the study population was 66.6±16.2 years, 63.8% of the patients were female. Mean time of hospitalization was 12.4±12.8 days. Postoperative SICU admission was necessary in 23 cases (24.5%). Two patients passed away during their hospital admission (2.1%). Indicators associated with the need for SICU treatment are the detection of pleural effusion in preoperative radiologic imaging (n=9 (39.1%) in SICU group vs. n=7 (9.9%) in no-SICU group; *p= .003),* intraoperative intestinal resection (n=6 (26.1%) vs. n=6 (8.6%); *p= .066*) and pre-existing atrial fibrillation (n=8 (34.8%) vs. n=11 (15.5%); *p= .07)*. SICU was mainly admitted with pulmonary indication (n=9 / 39.1%), cardiovascular indication (n=7 / 30.4%), or with septic shock (n=5 / 21.7%). One patient was admitted with neurological indication, one with liver failure.

**Conclusion:**
Patients with mechanical SBO caused by adhesive bands are likely to undergo postoperative treatment and surveillance in a SICU. Quick and reliable decision parameters are urgently needed in clinical practice. As shown, the preoperative detection of pleural effusions, intraoperative intestinal resection, and pre-existing atrial fibrillation are associated with the need for SICU treatment in our collective and could thus be used as assisting parameters for an evidence-based decision making. Prospective trials are needed to further investigate the need of and indications for postoperative SICU treatment.

1. Nates, J.L., et al., *ICU Admission, Discharge, and Triage Guidelines: A Framework to Enhance Clinical Operations, Development of Institutional Policies, and Further Research.* Crit Care Med, 2016. **44**(8): p. 1553-602.

### Combining Robot-Assistance and Ultrasound-Guidance for Peripheral Endovascular Interventions – An Update of the RoGUS-PAD-Project

ID: 154

**Kategorie: DGCH - Computergestützte Chirurgie, Robotik, Digitalisierung, Virtual Reality, KI**

Mark Preuß[1], Marco Horn[2], Jonas Osburg[3], Floris Ernst[3], Markus Kleemann[4]

[1]*Universitätsklinikum Hamburg-Eppendorf, Department of Vascular Medicine, Hamburg, Deutschland*, [2]*Universitätsklinikum Schleswig-Holstein, Campus Lübeck, Lübeck, Deutschland*, [3]*Universität zu Lübeck, Lübeck, Deutschland*, [4]*Dr. Erler Kliniken, Nürnberg, Deutschland*

**Background:**

The use of ultrasound is a promising approach to reduce radiation exposure in peripheral endovascular surgery. During endovascular interventions, however, it is inconvenient to operate the endovascular devices and guide the ultrasound probe in parallel. Therefore, an assistance system will be useful to control the ultrasound probe. The development of collaborative robots with highly sensitive force-torque sensors has made it possible to use them in direct contact with humans. Thus, a robotic assistance system that takes over the transducer guidance during the intervention is an ideal solution. Aim of the RoGUS-PAD-Project is the development of a robot-based assistance system to support ultrasound-guided peripheral endovascular interventions.

**Materials and methods:**

In cooperation with the Institute for Robotics and Cognitive Systems at the University of Lübeck, we started the development of an (intelligent) transducer control system for duplex-guided peripheral endovascular interventions.
A 2D ultrasound linear probe (Philips Epiq 7 with Philips XL14-3 probe, Philips Healthcare, Best, Netherlands) was mounted to the end effector of a robot arm (LBR iiwa 7 R800, KUKA, Augsburg, Germany).
For the pilot studies we used a specially designed ultrasound-capable phantom.

**Results:**

In the first step we tested the technical feasibility of the project by performing a semi-automatic 2D ultrasound scan of a peripheral artery on the phantom.
In the next step a 3D U-net was trained to detect and follow the guidewire. Input data were the ultrasound volumes with the guidewire inserted. Output data were the binary representations of the wire. In total xyz data samples are used for training. The data set was splitted up for training, validation and test data.

**Conclusion:**

The new results of the pilot study support the thesis that the development of a robot-based assistance system for ultrasound-guided peripheral endovascular interventions is technically feasible.

### Evaluation of ergonomics and surgeon's stress levels during robotic assisted minimally invasive esophagectomy – the ERASE trial

ID: 217

**Kategorie: DGCH - Computergestützte Chirurgie, Robotik, Digitalisierung, Virtual Reality, KI**

Dolores Thea Müller[1], Juliane Ahn[2], Stefanie Brunner[1], Jennifer Eckhoff[1], Christian Storms[1], Julia Poggemeier[1], Thomas Schmidt[1], Christiane J. Bruns[1], Hans Fuchs[1]

[1]*Klinik für Allgemein-, Viszeral-, Tumor- und Transplantationschirurgie, Universität zu Köln, Medizinische Fakultät und Uniklinik Köln, Köln, Deutschland*, [2]*International School of Design Köln , Technische Hochschule Köln , Köln , Deutschland*

**Background:**

Although often underestimated and even forgotten in everyday routine, especially in a surgical work environment, ergonomics are essential for endurance, efficiency, and a long and healthy work life. According to current studies, the rate of musculoskeletal disorders among minimally invasive surgeons ranges between 23-80%. An efficient and ergonomic design of the operating room has been widely discussed and guidelines have been imposed to create the optimal workspace. However, while all surgeons in a previous survey stated ergonomics to be important and affecting their everyday life, 89% lacked awareness of the guidelines. With the implementation of technical advancements and the introduction of robotic operating techniques, ergonomics have also become an essential differentiating factor between systems.

Current guidelines and recommendations for ergonomics for robotic devices were adopted from working at the microscope, as the body posture mimics the use of the robot. Sitting at the DaVinci Xi surgeon console, it is recommended to maintain a 90-degree angle at the knee joint, keep the upper arms perpendicular to the floor, with elbows close to the body and bent at a 90-degree angle with forearms resting on the arm rest.

The ERASE trial (**E**rgonomics in **R**obotic **AS**issted minimally invasive **E**sophagectomy) aims to analyze the surgeon's posture in combination with the intraoperative subjective and objective stress level and thus identify the ergonomic requirements in the robotic operating room. Data is further synchronized and compared to open surgery to draw conclusions about the workload of surgeons using a robotic device.

**Materials and methods:**

A variety of validated intraoperative measurements evaluating the surgeon and their interaction with the new technology are applied. Measurements do not compromise the surgeon's freedom of movement nor compromise patient safety.

Heart Rate Variability (HRV) represents an objective and validated parameter for assessing psychological distress of the human body while performing a certain task. It is defined as the physiological variation between temporal intervals of heartbeats and is linked to a complex neurovisceral integration model reflecting the interaction of various components of a complex system. HRV can easily be recorded with a chest band and corresponding sensor (i.e. Polar Watch Chest Band). The obtained data is evaluated using device specific software.

For the subjective evaluation of stress level, surgeons are asked to fill out a short form of the State-Trait-Anxiety-Inventory (STAI) questionnaire, which reflects momentary stress. The pre- and postoperative use of the questionnaire reflects the influence the surgical procedure has on psychological stress.

For evaluation of posture, a camera is used to capture the surgeon at the console during surgery. Using video editing programs, an image analyses can be created showing the average posture of the surgeon. Angles of the main body joints are further calculated and a validated scale stratifying measured results into four risk categories is then applied.

In addition, a touch sensor is attached to the surgeon console measuring if and how long the arm rest is used. The data is wirelessly transferred to a cloud based excel spread sheet for analysis. Furthermore, during all surgeries, sound volume in the operating room is measured continuously and then synchronized with the corresponding data. The measuring device is in direct proximity to the surgeon console, recording the noise the surgeon is exposed to during surgery.

For evaluation of technical feasibility, the OR time, time to complete specific surgical steps, and operative outcome is analyzed. Video recordings of the endoscopic camera are obtained and synchronized with the obtained data to supplement the analysis. A video summarizing the innovative study design and analysis of results is available.

**Results:**
Preliminary results of the ERASE trial were able to show a significant decrease in the surgeon’s heart rate a few seconds after the surgery started, which was maintained while the surgical task was performed. A slight increase was seen when a minor bleed occurred. Complex tasks such as the anastomotic phase, the use of a stapling device, and consecutive suturing did not show a significant increase in heart rate.

As can be seen in the video, certain parts of the surgery are accompanied by a low-risk posture. A 90-degree angle at the knee joint is maintained, upper arms are kept perpendicular to the floor, and the elbows are bent at a 90-degree angle with forearms resting on the arm rest.
In other parts of the surgery a critical posture is seen. Further analyses have yet to reveal if a certain surgical phase is associated with a poor ergonomic posture and therefore a high risk of musculoskeletal disorders.

Analysis of the data from the touch sensor revealed that the arm rest is used in less than 25% of the time during robotic surgery, indicating poor surgical ergonomics.

**Conclusion:**
Surgical ergonomics are an important aspect of the surgeon’s everyday work life, however, no validated and standardized measurements evaluating ergonomics in the operating room exist. Preliminary results of our study revealed the need for improvement of ergonomics. The ERASE trial is currently recruiting and further analysis and application of the presented methods to future cases of robotic assisted minimally invasive esophagectomy will be performed. A video summarizing the innovative study design and analysis of results is available.

**Novel machine learning algorithm can identify patients at risk of poor overall survival following curative resection for colorectal liver metastases.**
ID: 220

**Kategorie: DGCH - Computergestützte Chirurgie, Robotik, Digitalisierung, Virtual Reality, KI**

Iakovos Amygdalos[1], Gustav Anton Müller-Franzes[2], Jan Bednarsch[1], Zoltan Czigany[1], Tom Florian Ulmer[1], Philipp Bruners[2], Christiane Kuhl[2], Ulf Neumann[1], Daniel Truhn[2], Sven Arke Lang[1]

[1]*Uniklinik RWTH Aachen, Allgemein-, Viszeral-, und Transplantationschirurgie, Aachen, Deutschland*,
[2]*Uniklinik RWTH Aachen, Diagnostische und Interventionelle Radiologie, Aachen, Deutschland*

**Background:**
The primary cause of mortality in colorectal cancer is metastatic disease. We investigated the ability of a machine learning (ML) algorithm to stratify overall survival (OS) of patients undergoing curative resection for colorectal liver metastases (CRLM).

**Materials and methods:**
Patients undergoing curative liver resection for CRLM between 2010-2021 at the University Hospital RWTH Aachen were eligible for this retrospective study. Patients with recurrent metastases, incomplete resections, or early deaths, were excluded. A gradient-boosted decision tree (GBDT) model identified patients at risk of poor OS, based on clinicopathological characteristics. Differences in survival were compared with Kaplan-Meier analysis and the log-rank test.

**Results:**
A total of 487 patients were split into training (n=389, 80%) and test cohorts (n=98, 20%). Of the latter, 20(20%) were identified by the GBDT model as high-risk and showed significantly reduced OS (23 months vs. 52 months, p=0.005) and increased hazard ratio (2.434, 95%CI 1.280-4.627, p=0.007). The strongest predictors were preoperative serum carcinoembryonic antigen (CEA), age, diameter of the largest metastasis, number of metastases, body mass index, and primary tumor grading.

**Conclusion:**
A GBDT model can identify high-risk patients regarding OS after curative resection of CRLM. Closer follow-up and aggressive systemic treatment strategies may be beneficial to these patients.

**Haptic improvement of surgical instruments in robotic assisted surgery by using optical pressure sensors for the visualization of acting forces.**
ID: 335

**Kategorie: DGCH - Computergestützte Chirurgie, Robotik, Digitalisierung, Virtual Reality, KI**

Ernst Kolbe[1], Christian Rabe[2], Thomas Wellmann[1], Ruoyu Zhang[1], Günther Winde[1]

[1]*Universitätsklinik f. Allgemein- und Viszeralchirurgie, Thoraxchirurgie und Proktologie der Ruhr-Uni-Bochum, Klinikum Herford, Herford, Deutschland*, [2]*Fraunhofers Institut für Angewandte Polymerforschung, Abteilung chromogene Polymere, Potsadam, Deutschland*

**Background:**
Robotic surgery is the latest development in abdominal minimal invasive surgery. The major problem concerning moving of the intraabdominal instruments is the lack of haptic feedback during the tissue handling by the instrumental tips. Only extensive experience from other surgical techniques gives an indirect feeling on how much tension or pressure is compatible without causing harmful damage to the tissue. This lack of haptic needs improvement to increase intraoperative safety.

**Materials and methods:**
Therefore, ultrasensitive optical sensor switches are attached to the finger-tip of the handling devices of the robot’s commanding unit to give digital signals under finger to generate color signals on the surgeons monitor to indicate the applied force to the tissues.
No changes to the robotic device are necessary (here: da Vinci xi-System, Intuitive Surgical, Inc.,Sunnyvale, CA, USA). The handling device with the finger-loops for instrumental movements were loaded with optical pressure sensor switches (LED, photosensitive resistor, flexible medium and pressure knob in a case). Movement of the finger-tip changes the transparency of the flexible sensor material inside and changes of the light intensity transmitted by two LED-strips to the optical resistor (1S balancer, AC-DC amplifier for 8V, Arduino nano board., Bluetooth module HC05 HC06 wireless RF-transceiver RS232, Arduino UNO Rev3, C++ protocol). Signals are logged using a data logger shield. The unit is clamped to the robot handling devices without disturbing their movements.

**Results:**
Applied forces to the photosensitive switches were transformed into visible color signals. The programming allows changes of tactile forces for grasping and for tension to the tissue. Changes of various materials of the flexible medium in the photosensitive switches enables changes of the power displayed in a linear, degressive or progressive signal presentation.

**Conclusion:**
This haptic device is unique. It could be used in training centers by mentors or together with proctors on surgical sites. The system is priceworthy and does not afford changes to sterilization processes.

**Robotic-Assisted versus Laparoscopic Left Hemicolectomy-Postoperative Inflammation Status, Short-Term Outcome and Cost Effectiveness**
ID: 342

**Kategorie: DGCH - Computergestützte Chirurgie, Robotik, Digitalisierung, Virtual Reality, KI**

Sven Flemming[1], Matthias Kelm[1], Anna Widder[1], Niels Matthes[1], Joachim Reibetanz[1], Christoph-Thomas Germer[1], Florian Seyfried[1], Armin Wiegering[1]

[1]*Department of General, Visceral, Transplantat, Vascular and Pediatric Surgery, Center of Operative Medicine (ZOM),, University Hospital of Wuerzburg, Würzburg, Deutschland*

**Background:**
Robotic surgery has gained momentum worldwide with fast-growing expansions in various disciplines during the last decade. While a minimal-invasive approach compared to open surgery has been shown to be vastly superior regarding short-term outcomes, increasing evidence also demonstrates comparable long-term results for oncological and non-oncological indications. Despite robotic surgery offering additional benefits, including elimination of natural tremor with significantly improved visualization, better ergonomics and enhanced dexterity, robust evidence demonstrating that robotic surgery can further increase the advantages of laparoscopic surgery is lacking. Despite the lack of evidence for the superiority of robotic-assisted surgery, numbers of robotic procedures are rapidly increasing, and novel fields, including colon, gastric and pancreatic surgery, are evolving. While there are consistent improvements in the technical and surgical aspects of robotic-assisted surgery, criticisms of those mostly non-evidence-based developments remain relevant and are not deniable. While operating times are significantly prolonged for robotic-assisted approaches, enhanced costs of robotic-assisted surgery in comparison to laparoscopic surgery without clear evidence for an improved short- and long-term outcome remain another major issue. Therefore, the advantage of robotic surgery in comparison to laparoscopic surgery for standardized minimally invasive procedures in visceral surgery still needs to be determined. This study aimed to analyze postoperative inflammation status, short-term outcome and cost-effectiveness of robotic-assisted versus laparoscopic left hemicolectomy.

**Materials and methods:**
All consecutive patients who received minimal-invasive left hemicolectomy at the Department of Surgery I at the University Hospital of Wuerzburg in 2021 were prospectively included. Importantly, no patient selection for either procedure was carried out. The robotic-assisted versus laparoscopic approaches were compared head to head for postoperative short-term outcomes as well as cost-effectiveness. The primary endpoint was defined as the length of hospital stay. Secondary endpoints were postoperative complications within 30 days, including MTL30. Furthermore, serum levels of leukocytes and C-reactive protein (CRP) were collected on postoperative days 1, 3 and 5.

**Results:**
A total of 61 patients were included, with 26 patients having received a robotic-assisted approach. Baseline characteristics did not differ among the groups. Operating times in patients receiving robotic-assisted left hemicolectomy were significantly longer compared to patients who were operated on laparoscopically (254 min versus 173 min; $p = 0.001$). However, rates of conversion were comparable between both groups (11.4% versus 11.5%; $p = 0.989$). Length of hospital stay, the primary endpoint of patients receiving robotic-assisted surgery, was significantly reduced compared to patients who underwent laparoscopic resection (6 versus 10 days, $p = 0.025$). In a multivariate analysis, this trend was confirmed without reaching statistical significance. Similarly, laparoscopic surgery tended to increase levels of postoperative complications such as anastomotic leakage (8.6% versus 0%, $p = 0.126$). Serum levels of leukocytes and C-reactive protein (CRP) were analyzed during the postoperative course to evaluate differences in inflammatory responses of patients receiving minimally invasive surgery. While on postoperative day 3, serum levels of CRP trended to be lower following robotic-assisted surgery in comparison to laparoscopic resection, CRP levels were significantly decreased on postoperative day 5 in patients who received robotic-assisted colon resection. No differences in serum levels of leukocytes were observed between both groups. In a multivariate analysis, decreased levels of CRP at postoperative days 3 and 5 were identified as independent prognostic factors. Interestingly, despite higher costs for the robotic-assisted procedure itself (EUR 1412.83 versus EUR 2017.17), overall costs were lower when patients were operated on in a robotic-assisted manner (EUR 6796.37 versus EUR 6559.89), with a cost-effectiveness of EUR 236.48 per patient. The main difference regarding cost-effectiveness between both groups was seen in the costs of the hospital stay, which were much higher for laparoscopic surgery (EUR 3455.50 versus EUR 2073.20). Importantly, costs for the robotic system, including acquisition and maintenance, were calculated per patient over a period of ten years with eight operating days per month.

**Conclusion:**

In conclusion, we demonstrated in a single-center study that robotic-assisted left hemicolectomy resulted in decreased length of hospital stay and trended to lower rates of complications, leading to improved cost-effectiveness. Based on that, our study supports the ongoing implementation of robotic-assisted colon surgery, but further prospective studies are necessary to confirm the results and to evaluate the mechanistic background of a potentially decreased inflammatory stress response.

**A Data Management Guideline for Application of Artificial Intelligence to Robotic Assisted Minimal Invasive Esophagectomy (RAMIE) Video Data**
ID: 399

**Kategorie: DGCH - Computergestützte Chirurgie, Robotik, Digitalisierung, Virtual Reality, KI**

Jennifer Eckhoff[1,2], Yinan Xu[1], Dolores Müller[1], Alissa Reisewitz[1], Julia Poggemeier[1], Yue Zhao[3], Christiane Bruns[3], Hans Fuchs[1]

[1]*Department of General, Visceral, Cancer and Transplantation Surgery, University Hospital Cologne, Cologne , Deutschland*, [2]*Surgical Artificial Intelligence and Innovation Laboratory, Massachusetts General Hospital, Boston, United States*, [3]*Surgical Research Laboratory, Department of General, Visceral, Cancer and Transplantation Surgery, University Hospital Cologne, Cologne, Deutschland*

**Background:**
With over 70 robotic assisted minimally invasive esophagectomies (RAMIE) performed at the University Hospital Cologne each year, there is opportunity for exploration of the video data for research, education and clinical evaluation. Specifically, the application of Artificial Intelligence (AI) to surgical video data promises to provide intraoperative decision support, mitigate operative risk and augment surgical training. However, to increase generalizability of AI algorithms and enable thorough comprehension of surgical workflow, algorithms need to be presented with diverse training data accounting for various surgical scenarios and patient factors. We provide a detailed guideline to the composition of a surgical dataset for RAMIE, accounting for clinical variability as well as providing suitability for various AI applications. This infrastructure will serve as a starting point for minimally invasive surgeons to build their own datasets, facilitate data acquisition, storage and management, and subsequently promote data sharing and data diversity.

**Materials and methods:**
We evaluated a hierarchical team-based approach for effortless recording and data collection of all performed RAMIE cases in the operating from. Various contributors were involved in video recording and data collection, ranging from medical students to nursing staff. Throughout the process we composed a recording manual, which serves as instructions for the recording process and provided troubleshooting for encountered technical difficulties. Two recording systems were addressed for the two parts of RAMIE: the Stryker SDC3 Recording System was used for recording of the laparoscopic conduit preparation from the Stryker platform and the thoracic part was recorded using the Medicap USB300 from the Da Vinci Xi platform. We report on a concise data storage and preprocessing pipeline, including the video format conversion, data cropping and merging, and de-identification of the data to comply with privacy regulations and secure link to clinical data. Additionally, we elaborate on our annotation pipeline of RAMIE video data with respect to the desired clinically-relevant target features to build the dataset for AI model training and validation.

**Results:**
Since the successful implementation of a surgical video data infrastructure in 01/2022, a dataset of 50 RAMIE cases were collected and used for AI research in surgical phase recognition and tool detection. The video acquisition, data preprocessing, and annotation in our center led to the generation of a successful data management guideline, which will help to compose a databank analogous to other biodata sources. Besides that, we present a recording manual, which can be adapted to various recording technologies and deployed in any operating room for surgical video recording, leading to the generation of large and diverse datasets and facilitating future AI research. We elaborate on various pitfalls throughout the implementation of this infrastructure and how to overcome them.

**Conclusion:**
This infrastructure and data management guideline for the composition of a RAMIE databank will support future AI-based research for intraoperative risk mitigation in the operating room. The infrastructure we elaborate on may serve as a basis for future projects and can be extrapolated to many different procedures and institutions. Surgical video data is an essential data source for clinical outcome improvement and should be collected for research, education and patient care. Standardization and Automation of data collection and management pipelines are essential to ensure holistic datasets and comply with ethical and legal regulations.

**The SAlzburg PEritoneal SUrface CAlculator (SAPESUCA): The first web-based application for peritoneal surface quantification in cytoreduction and HIPEC**
ID: 421

**Kategorie: DGCH - Computergestützte Chirurgie, Robotik, Digitalisierung, Virtual Reality, KI**

Tarkan Jäger[1], Stephan Regenbogen[1,2], Philipp Schredl[1], Daniel Neureiter[3], Klaus Emmanuel[1], Jaroslav Presl[1], Jan Philipp Ramspott[1,4]

[1]*Department of Surgery, Paracelsus Medical University Salzburg, Salzburg, Österreich*, [2]*Department for Trauma Surgery, BG Trauma Center Murnau, Murnau, Deutschland*, [3]*Institute of Pathology, Paracelsus Medical University Salzburg, Salzburg, Österreich*, [4]*Department for General, Visceral and Transplant Surgery, University Hospital Muenster, Muenster, Deutschland*

**Background:**
One challenge of cytoreductive surgery (CRS) and hyperthermic intraperitoneal chemotherapy (HIPEC) in patients with peritoneal surface malignancies remains the high inter- and intraindividual resection variability of the peritoneal surface area (PSA) and its standardized documentation. To reduce this variability, we developed the SAlzburg PEritoneal Surface CAlculator (SAPESUCA).

**Materials and methods:**
SAPESUCA was programmed with R-Shiny framework. The programming-algorithm incorporates patient's body surface area (BSA) and its correlated peritoneal surface area (PSA) based on the 13 peritoneal cancer index (PCI) regions.

**Results:**
Forty colorectal cancer patients with peritoneal metastasis (pmCRC), who underwent CRS and HIPEC between 2012 and 2020 were extracted from our prospectively maintained international HIPEC registry. Patients median age was 55 years (interquartile range [IQR] 47-62 years). Median PCI score was 10 (IQR 5-17). SAPESUCA revealed a mean PSA of 19,093 cm2 ± 2,069 of all patients before CRS compared to 13,906 cm2 ± 4,998 after CRS (reduction rate 27%). Highest peritonectomy extent was found in the central PCI region (1,567 cm2 ± 801), whereas upper jejunum region showed lowest extent (24 cm2 ± 51).

**Conclusion:**
SAPESUCA is the first free web-based application for standardized determination and thus comparability of the resected and remaining PSA after CRS. Multicentric application and ongoing research of SAPESUCA is necessary for further validation.

**Hyperspectral imaging and machine learning for evaluation of gastric venous congestion after total pancreatectomy**
ID: 523

**Kategorie: DGCH - Computergestützte Chirurgie, Robotik, Digitalisierung, Virtual Reality, KI**

Amila Cizmic[1], Patrick Fariwar[1], Marc Bressan[1], Berkin Özdemir[1], Jan Odenthal[1], Gabriel Salg[1], Silvia Seidlitz[2], Jan Sellner[2], Leonardo Ayala[2], Lena Maier-Hein[2], Beat Müller-Stich[1], Alexander Studier-Fischer[1], Felix Nickel[1]

[1]*Heidelberg University Hospital, Department of General-, Visceral- and Transplantation Surgery, Heidelberg, Deutschland*, [2]*Division of Computer Assisted Medical Interventions, German Cancer Research Center, Heidelberg, Deutschland*

**Background:**
Total pancreatectomy can be indicated for both malignant and benign pancreatic diseases. When spleen preservation is not possible for oncological or technical reasons there is a risk for venous congestion of the stomach as venous drainage might be impaired. This is especially important when left gastric vein and right gastroepiploic vein cannot be preserved or reconstructed. Impaired gastric perfusion and venous drainage can lead to impaired stomach function, but also to gastric necrosis and anastomotic insufficiency. Partial or total gastrectomy can be performed, if deemed necessary intraoperatively to avoid these complications, but will impair quality of life. Intraoperative visual inspection of gastric perfusion and venous congestion is limited by its subjective nature. The existence of an intraoperative evaluation tool that could already identify venous congestion beyond the level of gastric tolerance would aid in avoiding postoperative complications, but could also avoid unnecessary gastric resections. Hyperspectral Imaging (HSI) is an electromagnetic surface detection method allowing evaluation of oxygenation and perfusion on a microvascular level and therefore has the potential to intraoperatively detect venous congestion of the stomach wall.

**Materials and methods:**
In a porcine model, HSI spectral reflectance baseline data was collected for (1.) physiological stomach, (2.) avascular stomach (complete ligation of all vessels) and (3.) stomach with venous congestion (ligation of all structures except arteries) across 54, 14 and 5 in-vivo pig models in over 600 recordings. This was done in order to obtain the true spectral reflectance fingerprints of the porcine stomach in various perfusion states. With the knowledge of what the true spectral signature of venously congested stomach is, now total pancreatectomy was performed in 5 pigs (4.). Spectral reflectance data was collected over a period of one hour in order to evaluate if total pancreatectomy in in-vivo pig models can actually reproduce gastric venous congestion (3.). Oxygenation index values ($StO_2$) were extracted from all groups and a machine learning algorithm was trained to visualize and differentiate these using principal component analysis (PCA).

**Results:**
Spectral reflectance between the 3 baseline groups (Figure 1) was different and characteristic. $StO_2$ values showed significant differences with 72.36% (± 9.16%) for physiological stomach (1.), 34.61% (± 6.07%) for avascular stomach (2.) and 65.53% (± 8.25%) for stomach with venous congestion (3.). Machine learning algorithms were able to differentiate these three groups with a great degree of explained variance of 89.17% via PCA. Spectral reflectance of stomach after total pancreatectomy (4.) in the porcine model was found to be in the middle of the spectrum between physiological stomach (1.) and stomach with complete venous congestion (3).

**Conclusion:**
Different states of gastric perfusion can be differentiated using HSI. Total pancreatectomy in an in-vivo porcine models appears to induces a certain degree of venous congestion and therefore has the potential to be an adequate model to investigate the phenomenon of venous congestion after total pancreatectomy in patients. HSI with machine learning is capable of identifying this venous congestion. In order to properly quantify the precision and predictive value of HSI for clinically relevant congestion, experimental groups will be further expanded and compensation mechanisms for venous congestion will be evaluated systematically in the porcine model. Effects on patient outcome of intraoperative HSI measurements will have to be further investigated in a clinical trial.

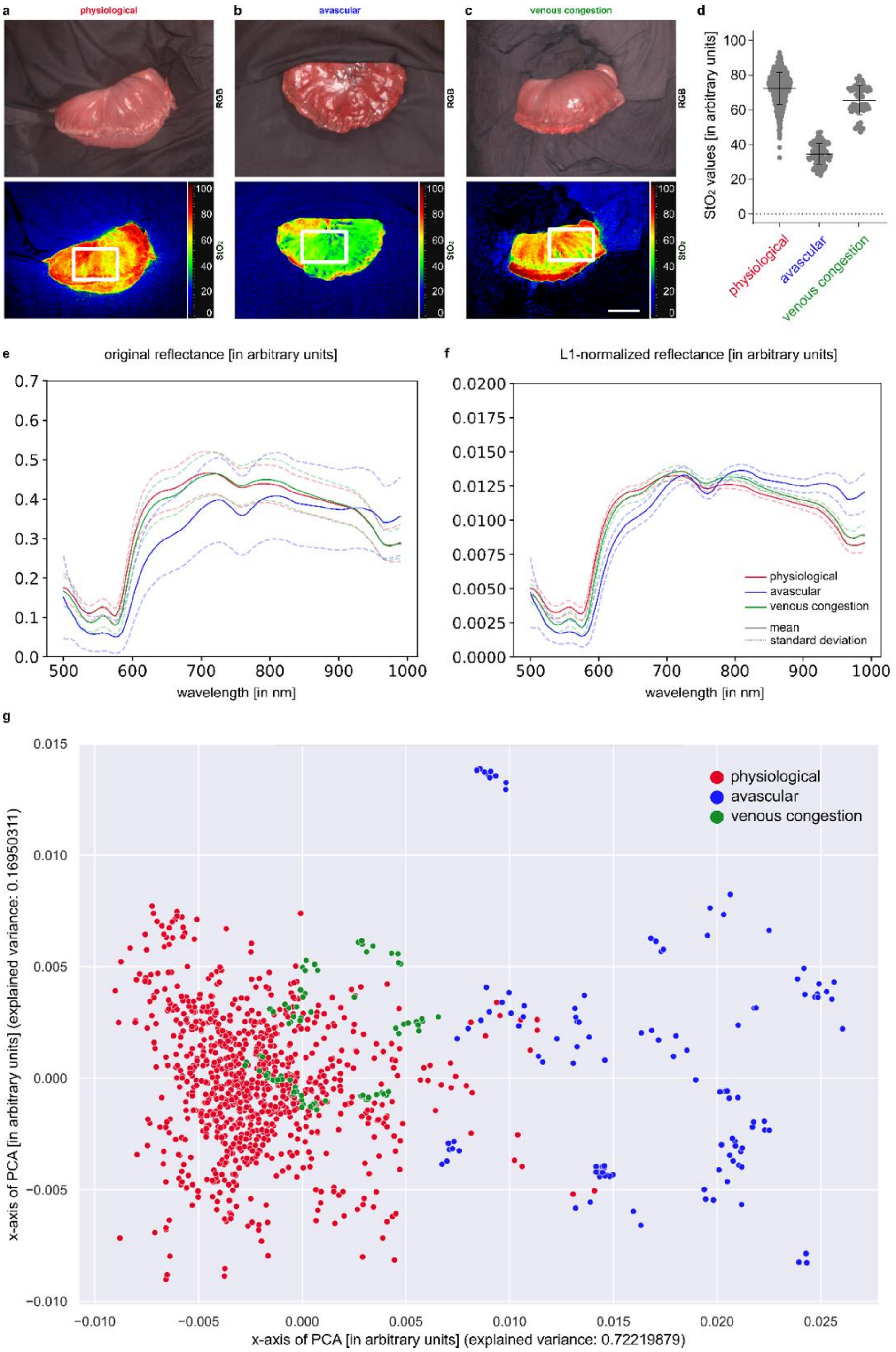

**Figure 1 | Baseline of stomach data.** HSI Color-Index pictures and respective spectra for porcine stomach. **a**, physiological stomach (A = 54). **b**, avascular stomach (A = 14). **c**, stomach with venous congestion (A = 5). **d**, quantification of hyperspectral index values for $StO_2$. **e-f**, overlay of reflectance spectra. **g**, principal component analysis of baseline groups. Scale bar equals 5 cm.

**Intraoperative extent of resection control with cone-beam computed tomography in pituitary adenoma surgery**
ID: 552

**Kategorie: DGCH - Computergestützte Chirurgie, Robotik, Digitalisierung, Virtual Reality, KI**

Laurèl Rauschenbach[1], Marvin Darkwah Oppong[1], Ramazan Jabbarli[1], Ulrich Sure[1], Karsten Henning Wrede[1]

[1]*Universitätsmedizin Essen, Klinik für Neurochirurgie und Wirbelsäulenchirurgie, Essen, Deutschland*

**Background:**
Cone-beam computed tomography (CBCT) is increasingly used for intraoperative real-time bone imaging. Unfortunately, the spatial resolution of soft tissue is limited due to several imaging artifacts. In this feasibility study on pituitary adenomas, the applicability of CBCT technology was investigated with regard to the visu-alization of tumors and tumor remnants.

**Materials and methods:**
The study comprised six consecutive patients with sellar lesions suspicious of pituitary adenomas that un-derwent endoscopic neuronavigation-guided transnasal transsphenoidal surgery. Intraoperative contrast-enhanced CBCT (C-arm ARTIS Pheno, Siemens Healthcare GmbH, Erlangen, Germany) was used to visual-ize tumor masses before and after resection. Patients received early postoperative magnetic resonance imaging (MRI) to determine the extent of resection. Tumor remnant visualization was compared between CBCT and MRI.

**Results:**
Intraoperative and postoperative imaging could be performed without complications in all six patients. Intraoperative CBCT allowed high-resolution imaging of the sellar lesions and reliably visualized tumor remnants. Comparison between intraoperative CBCT and early postoperative MRI revealed high diagnostic accuracy of CBCT.

**Conclusion:**
Intraoperative CBCT is a promising tool to assess the extent of resection in pituitary adenoma surgery. Studies with a larger sample size are warranted to validate the value of CBCT in pituitary adenoma surgery.

**RoCS - Robotic Curriculum for young Surgeons**
ID: 656

**Kategorie: DGCH - Computergestützte Chirurgie, Robotik, Digitalisierung, Virtual Reality, KI**

Jessica Stockheim[1], Roland Croner[1]

[1]*Universitätsklinikum Magdeburg, Klinik für Allgemein-, Viszeral-, Gefäß- und Transplantationschirurgie, Magdeburg, Deutschland*

**Background:**
Robotic-assisted procedures gain increasing acceptance for daily surgical routine. However, structured training programs are designed for surgeons with high expertise. Hence, a comprehensive training curriculum was established to ensure a basic competence in robotic abdominal surgery for novice surgeons during their residency. The aim of the current work is to propose a feasible and effective training concept.

**Materials and methods:**
The development process of this training curriculum is based on a comprehensive literature review which led to the concept of "Robotic Curriculum for young Surgeons" (RoCS). It was implemented in the daily routine of a German university hospital starting in 2020. The robotic assessment questionnaire (RAQ) was used by electronic data collection via RedCap®. After the initial phase adjustments, it led to an improvement of the initial version of the curriculum.

**Results:**
RoCS is a multimodal training program containing basic training through assistance at the operation table during robotic assisted operations and basic console training. Key elements are the robotic team time-out (rTTO; performed preoperatively in 88,2% for table assistance), perioperative process standardization including feasible personnel scheduling (in 82,7% realized) and useful procedure clustering into organ systems, procedural steps and procedural step complexity. Evaluation of the parameters is realizable: standardized communication (postoperative feedback in 81,3% expert to assistant, in 83,6% assistant to expert), performance assessment (ability to perform assistance independently: overall table assistance in 42,6%, patient positioning in 82,1%, port placement/docking in 52,0%, intraoperative assistance in 74,8%), patient factors and individual overall workload using NASA Task Load Index (22,9 ± 19,4).

**Conclusion:**
Flexibility and adaptability to internal organization processes of surgical departments are the main advantages of the concept. RoCS is a strong training tool to meet the specific needs of young surgeons and evaluate their learning success of robotic procedural training. Furthermore, comparison within the different robotic systems should be considered. Further studies are needed to validate a multicenter concept design.

**Telestration with Augmented Reality improves Performance of repeated ex-vivo Porcine Laparoscopic Cholecystectomy in Novices: a Randomized Study**
ID: 682

**Kategorie: DGCH - Computergestützte Chirurgie, Robotik, Digitalisierung, Virtual Reality, KI**

Felix Müller[1], Amila Cizmic[1], Philipp Wise[1], Frida Häberle[2], Felix Gabel[2], Karl-Friedrich Kowalewski[3], Hannes Kenngott[1], Beat Müller[1], Felix Nickel[1]

[1]*Universität Heidelberg, Department of General, Visceral and Transplantation Surgery, Heidelberg , Deutschland*, [2]*Universität Heidelberg, Heidelberg, Deutschland*, [3]*University Medical Center Mannheim at Heidelberg University, Department of Urology, Mannheim, Deutschland*

**Background:**
The learning curve in minimally invasive surgery (MIS) is steep compared to open surgery. One of the reasons is that training in the operating room in MIS is limited to verbal instructions. The iSurgeon telestration device with augmented reality enables visual instructions, guidance, and feedback by experts during MIS procedures. This study aims to compare the effects of the iSurgeon on the training of trainees performing repeated laparoscopic cholecystectomy (LC) on a porcine liver compared to traditional verbal instruction methods.

**Materials and methods:**
Forty medical students were randomized into the study and the control group. The study group performed 10 LCs with iSurgeon visual instructions and guidance in addition to verbal instructions by trainers. The control group performed 10 LCs receiving conventional verbal guidance by trainers. Total operating time, complications, and performance assessment with Objective Structured Assessments of Technical Skills (OSATS) and Global Operative Assessment of Laparoscopic Skills (GOALS) scores were compared between the two groups. The difficulty of the LC was assessed using the Visual Analog Scale (VAS).

**Results:**
The study group with iSurgeon had significantly less intraoperative complications in total (2.7 ± 2.0 vs. 3.6 ± 2.0, $p < 0.001$) and during final (10$^{th}$) LC (1.7 ± 1.3 vs. 3.1 ± 2.2, $p < 0.001$). The study group with iSurgeon performed LC significantly better (global GOALS 17.3 ± vs. 16 ± 2.6, $p < 0.001$, LC specific GOALS 7 ± 2 vs. 5.9 ± 2.1, $p <0.001$, global OSATS 25.3 ± 4.3 vs. 23.5 ± 3.9, $p < 0.001$, LC specific OSATS 50.8 ± 11.1 vs. 41.2 ± 9.4, $p < 0.001$) compared to the control group. The study group had more difficult cases of the LC according to the VAS (51.48 ± 6.68 vs. 47.08 ± 8.38, $p < 0.001$).

**Conclusion:**
Visual expert instructions with telestration using the augmented reality system iSurgeon improves performance in LC training during the first ten LCs and even in difficult cases compared to conventional verbal expert guidance.

### The Evaluation of Vibroacoustic Signals Acquired with Surgical Audio Guidance Technology for Veress Needle Placement in Animal Cadaver Model

ID: 696

**Kategorie: DGCH - Computergestützte Chirurgie, Robotik, Digitalisierung, Virtual Reality, KI**

Moritz Spiller[1], Nazila Esmaeili[1], Thomas Sühn[1], Axel Boese[1,2], Michael Friebe[1,2,3], Alfredo Illanes[1], Salmai Turial[4]

[1]*SURAG Medical GmbH, Magdeburg, Deutschland*, [2]*INKA-Innovation Laboratory for Image Guided Therapy (IGTLAB), Medical Faculty, Otto-von-Guericke-University Magdeburg, Magdeburg, Deutschland*, [3]*Department of Measurement and Electronics, AGH University of Science and Technology, Kraków, Poland*, [4]*Division of Pediatric Surgery, Pediatric Trauma Surgery and Pediatric Urology, University Medical Centre Magdeburg, Magdeburg, Deutschland*

**Background:**

Needle-based procedures constitute a significant part of diagnostic and therapeutic minimally invasive interventions. These operations range from simple percutaneous needle insertion to more complex tissue biopsies. Several imaging and tracking guidance techniques have been integrated into these interventions to reach more accurate and optimum placement of needles. However, despite the recent advances, many needle procedures are still performed under no guidance, where the clinicians need to rely on their haptic sense and experience.

Surgical Audio Guidance (SURAG) is a novel sensing modality that uses vibroacoustic (VA) waves resulting from interactions between the surgical instrument tip and tissue [1]. The SURAG technology has been tested and validated in ex-vivo and synthetic phantoms for various needle-based procedures [2, 3, 4]. By processing the generated VA signal, the SURAG system can magnify in real-time needle tip-tissue interactions such as tissue-tissue passage and punctures.

In laparoscopy, the Veress needle has been used for decades as an access technique for establishing the pneumoperitoneum at the beginning of the procedure. However, the literature has reported up to 4% of accidental and even severe injuries of intra-abdominal organs due to the wrong insertion of the Veress needle. As another use case, the SURAG system has been connected to the proximal end of a Veress needle to analyze the VA signals generated from the needle peritoneum passage (cavity puncture event) in porcine ex-vivo tissue phantoms [5]. As this experiment was conducted under laboratory conditions, this study aims to verify the results of these evaluations on a model of the pig cadaver abdominal wall.

**Materials and methods:**

The Veress needle with the SURAG system connected at its proximal end was inserted into pig cadavers shortly after euthanasia. Another research group used the same pigs before euthanasia to perform different clinical experiments; however, these tests were conducted outside the abdominal cavity, so it did not affect our study. The required regulatory and ethical approvals were available for the original animal experimentation project.

The procedure started by marking 12 insertion points on the pig's abdominal area along the longitudinal axis, with a 2 cm lateral distance to the median line on each side. Then, every layer of the abdominal wall at the defined insertion points was verified and documented using a commercially available Ultrasound (US) device (LOGIQ e, GE Healthcare, Chicago, Illinois, United States). The skin at the puncture site was then incised with a scalpel to a length of approximately 5 mm so that the relatively thick and hard skin of pig carcass did not have to be punctured with the Veress needle. The Veress needle was then inserted at the marked insertion points until it was assumed that the abdominal cavity had been reached. The VA signal was continuously acquired and stored by the SURAG system during every needle insertion process. After each needle insertion, a small drop of a blue ink liquid was injected through the Veress needle to visualize the penetration depth and localization of the Veress needle tip on the peritoneal side for the subsequent assessment. After completing all needle insertions, the entire ventral abdominal wall of each pig cadaver was excised en bloc to examine and assess the Veress needle puncture points on the peritoneal side.
The acoustic signals acquired with the SURAG system were compared to the assessed peritoneal puncture to investigate the following hypotheses: a) a peritoneal cavity needle puncture results in a significant VA signal excitation, b) the VA excitation resulting from a peritoneal cavity puncture involve defined and identifiable characteristics. The data analysis was performed using advanced time and time-frequency domain signal processing techniques.

**Results:**
A total of 216 Veress needle insertions were conducted on nine pig cadavers weighing 30 to 70 kg during the first 30 minutes after the pigs' euthanasia. The data analysis showed that the cavity puncture event produces a unique signature in the VA signal that can be parametrized into four distinct phases representing: a) Veress needle inner-outer core friction, b) tissue layer breakage (puncture), c) Veress needle click, and d) post-puncture needle vibration. These phases occurred within an average of 60 milliseconds, from the moment the sharp Veress needle tip penetrates the abdominal cavity until the typical release of the clicking sound of the Veress needle (triggered by the spring movement of the protective sheath over the sharp tip). The presence or absence of each identified phase can allow the complete identification of a cavity puncture event.
Moreover, the assessment of the extracted abdominal wall of all pigs showed that the Veress needle had reached the peritoneal cavity in 194 (89.8%) insertions. In all these cases where the Veress needle has got to the abdominal cavity, a significant excitation in the VA signal was also registered by the SURAG system representing the cavity puncture event.

**Conclusion:**
The VA signals acquired by the SURAG sensing modality contain clear information regarding the Veress needle tip and tissue interactions in animal and human cadavers that can be used to identify cavity punctures automatically. This represents the potential of the SURAG technology to provide real-time feedback for peritoneum cavity targeting during Veress needle insertion.
In addition, the features related to the dynamic characteristics of these events can be used for automatic classification with artificial intelligence-based methods for future applications.

**Bibliography:**
[1] Illanes A, Sühn T, Esmaeili N, Maldonado I, Schaufler A, Chen CH, Boese A, Friebe M. Surgical Audio Guidance SurAG: extracting non-invasively meaningful guidance information during minimally invasive procedures. In2019 IEEE 19th international conference on bioinformatics and bioengineering (BIBE) 2019 Oct 28 (pp. 567-570). IEEE.
[2] Illanes A, Boese A, Maldonado I, Pashazadeh A, Schaufler A, Navab N, Friebe M. Novel clinical device tracking and tissue event characterization using proximally placed audio signal acquisition and processing. Scientific reports. 2018 Aug 13;8(1):1-1.
[3] Illanes A, Boese A, Friebe M, Hansen C. Feasibility check: can audio be a simple alternative to force-based feedback for needle guidance?. InInternational Conference on Medical Image Computing and Computer-Assisted Intervention 2020 Oct 4 (pp. 24-33). Springer, Cham.
[4] Sabieleish M, Heryan K, Boese A, Hansen C, Friebe M, Illanes A. Study of needle punctures into soft tissue through audio and force sensing: can audio be a simple alternative for needle guidance?. International Journal of Computer Assisted Radiology and Surgery. 2021 Oct;16(10):1683-97.
[5] Schaufler A, Sühn T, Esmaeili N, Boese A, Wex C, Croner R, Friebe M, Illanes A. Automatic differentiation between Veress needle events in laparoscopic access using proximally attached audio signal characterization. Current Directions in Biomedical Engineering. 2019 Sep 1;5(1):369-71.1.

**On-demand Robotic Gastrectomy with the Dexter Robotic SystemTM**
ID: 702

**Kategorie: DGCH - Computergestützte Chirurgie, Robotik, Digitalisierung, Virtual Reality, KI**

Felix Berlth[1], Giovanni Capovilla[1,2], Caterina Froiio[1,3], Sou Jing Kim[1], Lana Fourie[4,5], Richard van Hillegersberg[4], Peter Grimminger[1]

[1]*Department of General, Visceral and Transplant Surgery, University Medical Center Mainz, Mainz, Deutschland*, [2]*Department Surgery, University of Padova, Clinica Chirurgica 3°, Padova, Italy*, [3]*Department of General Surgery , IRCCS Policlinico San Donato, University of Milan , Milano, Italy*, [4]*Department of Surgery, University Medical Center Utrecht, Utrecht, , Utrecht, Netherlands*, [5]*Clarunis, Department of Visceral Surgery, University Centre for Gastrointestinal and Liver Diseases,, St. Clara Hospital and University Hospital Basel, Basel, Schweiz*

**Background:**
The Distalmotion Dexter Robotic System™ aims to combine benefits of robotic and laparoscopic surgery through fast and efficient changes of both settings. This study evaluates the feasibility by two cadaver gastrectomy cases and dexterity practice.

**Materials and methods:**
A group of seven surgeons, with and without robotic surgery experience participated the workshop. After introduction of the system four surgeons performed an intracorporeal stichting and knotting task. Each surgeon performed three knots and time was measured for each. Then after, two cadaver gastrectomy cases were performed by a team of two surgeons each. The procedures were performed under sterile conditions, similar to the real operation room setting. The procedure was divided into eleven steps and time was recorded for each step. During the procedure a change from laparoscopic to robotic and vice versa was performed three times.

**Results:**
The mean times of 1.59min (Knot 1), 1.37min (Knot 2) and 1.30min (Knot 3) indicate quick adoption to this new system. The gastrectomy was performed in 1h38min and 1h39min with consistent progress for each step of operation. The mean set up time (Incision to finish docking) was 7min08sec and the three changes of the setting (laparoscopic <-> robotic) were performed in 3min06sec, 1min27sec and 2min43sec, respectively. The robotically hand-sutured intracorporeal esophagojejunostomy took 17min01sec. Overall, 29min18sec of the procedure were performed laparoscopically and 55min41sec robotically

**Conclusion:**
This cadaver study suggests the feasibility of the Distalmotion Dexter Robotic System™ for complex surgery and indicates beneficial learning curve effects. A rapid change between laparoscopic and robotic setting is provided by the system.

**Modern Computer-assisted and KI-based Concepts for Surgical Simulation and Education with Hands-on Training Models in Neurosurgery**
ID: 743

**Kategorie: DGCH - Computergestützte Chirurgie, Robotik, Digitalisierung, Virtual Reality, KI**

Uwe Spetzger[1]

[1]*Neurochirurgische Klinik, Klinikum Karlsruhe & Karlsruhe Institute of Technology, KIT, Karlsruhe, Deutschland*

**Background:**
Practical training concepts are necessary to enhance skillful cranial and spinal surgery. At present, we have a rapidly ongoing specialization in any field of modern surgical therapy and need adequate practical tools for the surgical training.

Especially, in the field of vascular micro-neurosurgery. An increased number of patients treated successfully by endovascular techniques, consecutively reduce the possibility for adequate microsurgical training of the younger generation. A lack of expertise in cerebrovascular operations is the result. However, highly complex aneurysms with a wide neck will remain and will be a challenge for cerebrovascular neurosurgeons in the future. On the other hand, revascularization and bypass techniques will become more and more in demand.

Due to these conditions, concepts and realistic models for the microsurgical training are mandatory for modern teaching and training. On the other hand, more and more virtual models are available to train mental and manual skills.

**Materials and methods:**
We present a comprehensive overview of both our own models and the common commercially available. The PVC rat model (Microsurgical Developments Foundation, The Netherlands). The 3D printed skull and brain model - Deep Anastomosis Kit (Kezlex, Japan Medical Company, Japan). Aneurysm Box, UpSurgeOn, Italy). Additionally, a set-up of computer-assisted simulation and training modalities for neurosurgical education by using virtual-reality (VR) and augmented-reality (AR) for simulation and planning of brain tumor surgery is presented. Also, an easy but realistic “unbloody” navigation-guided training tool for training of virtual ventricular drainage is shown.

Additionally, different microsurgical in-vitro and in-vivo experimental hands-on training models will be demonstrated for experimental aneurysm clipping and microsurgical bypass surgery and practicing a ventricular drainage application.

**Results:**
Generally, all our demonstrated practical in-vitro training tools could easily be integrated into an educational surgical curriculum and improve the skillful practical training. Especially, the interactive learning in the navigation-guided virtual ventricular drainage model creates more understanding of this routine neurosurgical procedure and finally creates more safety for our patients.
The Aneurysm Box is good for beginners to learn and practice the craniotomy and understand the microsurgical approach. The PVC rat model is adequate for beginners and more experienced neurosurgeons to improve the technique of microvascular anastomosis. The Japanese 3D model gives the best haptic feedback and the vessels adequately mimic human conditions. The different microvascular training models are a prerequisite to start with highly complex surgical manipulation of aneurysm clipping and bypass surgery and teaches the young residents the adequate handling of the instruments. The complexity of the in-vitro and especially the demonstrated in-vivo models is stepwise adaptable and due to a rising complexity, these models are also useful for surgical experts, because these models maintain the skillfulness and are optimal for a live-long learning. Postoperatively, the final degree of clipping, as well as the patency rate of the anastomosis could be evaluated exactly and will demonstrate the level of the surgical expertise.

**Conclusion:**
The use of VR and AR simulation tools improve the practical education and training of young surgeons and enhance the safety of neurosurgical procedures in our daily routine. The microvascular in-vitro and in-vivo models are a perfect set-up for the advanced training of vascular neurosurgeons.

**Prediction of clinically relevant postoperative pancreatic fistula using radiomic features and preoperative data**
ID: 812

**Kategorie: DGCH - Computergestützte Chirurgie, Robotik, Digitalisierung, Virtual Reality, KI**

Fiona Kolbinger[1,2], Nithya Bhasker[3], Nadiia Skorobohach[4], Alex Zwanenburg[5], Steffen Löck[5], Jürgen Weitz[1,2], Stefanie Speidel[2,3], Marius Distler[1], Ralf-Thorsten Hoffmann[4], Stefan Leger[3], Jens-Peter Kühn[4]

[1]*Dresden, Department of Visceral, Thoracic and Vascular Surgery, University Hospital and Faculty of Medicine Carl Gustav Carus, Technische Universität Dresden, Dresden, Deutschland*, [2]*Dresden, Else Kröner Fresenius Center for Digital Health (EKFZ), Dresden, Deutschland*, [3]*Dresden, Division of Translational Surgical Oncology, National Center for Tumor Diseases (NCT), Partner Site Dresden, Dresden, Deutschland*, [4]*Dresden, Institute and Polyclinic for Diagnostic and Interventional Radiology, University Hospital Carl Gustav Carus Dresden, TU Dresden, Dresden, Deutschland*, [5]*Dresden, OncoRay - National Center for Radiation Research in Oncology, Faculty of Medicine and University Hospital Carl Gustav Carus, Technische Universität Dresden, Helmholtz-Zentrum Dresden-Rossendorf, Dresden, Deutschland*

**Background:**
Clinically relevant postoperative pancreatic fistula (CR-POPF) can significantly affect the treatment course and outcome in pancreatic cancer patients. Preoperative prediction of CR-POPF can aid surgical decision-making process and lead to better perioperative management of patients.

**Materials and methods:**
In this retrospective study, we have developed radiomic risk models that use preoperative computed tomography (CT)-based radiomic features in a cohort of 148 patients having undergone pancreaticoduodenectomy. The developed models were compared with risk models using preoperative clinical features and mesh-based volumes of the annotated structures. The model signatures were analysed in detail by visualising feature expression maps and comparing significant features to the standard CR-POPF risk measures.

**Results:**
The best average area under receiver operating characteristic curve (AUC) of 0.81 on validation data was achieved by the radiomic signature model retrained using the most important features. The following features showed significant correlation with outcome ($p < 0.05$): texture and morphology of healthy pancreatic segment, intensity volume histogram-based feature of pancreatic duct segment and morphology of combined segment; mesh-based volumes of healthy pancreatic segment, segment containing arteries, combined segment and pancreatic duct segment; BMI, and sex.

**Conclusion:**
The results of the study indicate that preoperative data can be useful for an accurate risk assessment of CR-POPF.

**Pediatric surgery care after 44 years of war in Afghanistan**
ID: 660

**Kategorie: DGCH - Humanitäre Hilfe der Chirurgie**

Turyalai Hakimi[1], Tareq Rahimi[2], Ajab Gul Momand[3], Salmai Turial[4]

[1]*The Maiwand University Teaching Hospital, Department of Pediatric Surgery, Kabul, Afghanistan*, [2]*The French Medical Institute for Mothers and Children- FMIC , Department of Pediatric Surgery, Kabul, Afghanistan*, [3]*The Indira Gandhi Children's hospital Kabul, Department of Pediatric Surgery, Kabul, Afghanistan*, [4]*Universitätsmedizin Magdeburg, Abteilung für Kinderchirurgie, Kindertraumatologie und Kinderurologie , Magdeburg, Deutschland*

**Background:**
Since 1978 (!), the Afghan population has been suffering from the continuous war actions with different international participations. The medical care as a whole and that of the children in particular has come to a standstill in this country. Within the framework of a humanitarian aid mission in the field of pediatric surgery in the capital Kabul as well as in the provinces of Herat and Farah, in the west of the country, an attempt was made to evaluate the pediatric surgical care. The results of a nationwide survey of data regarding pediatric surgical inpatient care in Afghanistan are presented.

**Materials and methods:**
All pediatric surgery departments in Afghanistan were contacted in person or by telephone and the head of each department was interviewed regarding the number of beds, number of medical staff, qualification of physicians, spectrum of departments, and about the important deficiencies from the pediatric surgery point of view. Furthermore, the help of the Association of Pediatric Surgeons in Afghanistan, based in Kabul, was sought for the purpose of the highest possible completion of the data.

**Results:**
Data collection took place between August and September 2022. A pediatric surgeon of Afghan origin working in a University Medical Centre in Germany who spent two weeks in Afghanistan in August 2022 coordinated the study
The pediatric surgery department at Maiwand University Teaching Hospital in Kabul conducted data collection. A request to the Ministry of Health in Kabul was sent.
There are five pediatric surgery departments in the entire Afghanistan, approximately 38.9 million population; all of them are located in the capital Kabul, only. In the rest of the country, 1.8 times larger than Germany in area, there is no specific pediatric surgical care and de facto no neonatal surgery at all. In the other four major cities of the country, there are pediatric surgeons working individually, but they are not exclusively dedicated to pediatric surgery. The adult surgeons integrated into the respective adult specialty department provide the limited care of surgically ill children.
Here, however, the necessary special knowledge of pediatric surgery is lacking and there are certainly no instruments suitable for children. In many of the provinces, with over a million inhabitants, there is no hospital at all, or only one very rudimentarily structured hospital. Pediatric intensive care, neonatal and preterm intensive care units or departments are non-existent in the provinces and in the periphery.
The pediatric surgery departments in Kabul have the following number of beds: The Maiwand University Teaching Hospital 20, the French Medical Institute for Mothers and Children- FMIC 36, the Indira Gandhi Children's hospital 70, the Irene Salimi Children's Hospital 25, the Atatürk National Children's Hospital 40 beds, respectively.
The five pediatric surgical departments in Kabul, as well as the hospital structures in the entire country, differ greatly in terms of equipment and resources: the facilities supported by the state are the least equipped, while the lack is less obvious in the facilities that are mostly supported by foreign aid organizations. The majority of children and newborns have no access to medical care, and those who survive the first year of life suffering from congenital malformations have little chance of receiving surgical care later on. Thus, there are many adolescents living in the country with untreated anal atresia or urogenital malformations, for example. Only very few parents can afford the temporary treatment in the neighboring countries and in India, unfortunately the results are also unsatisfactory, here for various reasons.

**Conclusion:**
Even now, and still in Afghanistan, a great many children die without reaching a hospital or in hospital without being able to receive the necessary diagnosis and treatment there because of marked deficiencies. There is an extreme distress and misery in this area in Afghanistan. Now, the help from outside, through the help of individuals, groups or small and large aid organizations, is urgently needed.

**INTACT-Lymph: Interventional approach to lymphatic leakage - current results**
ID: 163

**Kategorie: DGCH - Komplikationsmanagement in der Chirurgie**

Wilhelm Flatz[1], Max Seidensticker[1], Moritz Wildgruber[1], Sinan Deniz[1], Osman Oecal[1], Matthias Frölich[2], Jens Ricke[1]

[1]*Klinik und Poliklinik für Radiologie, Klinikum der Universität München, LMU München, München, Deutschland*, [2]*Klinik für Radiologie und Nukleasmedizin, Universitätsklinikum Mannheim, Mannheim, Deutschland*

**Background:**
Purpose of the study is to visualize lymphatic vessels and detect lymphatic leakage. To evaluate therapeutic potential of intranodal and CT-guided lymphatic embolisation.

**Materials and methods:**
In this prospective study 50 patients suffering from lymphatic leakage due to different causes (e.g. nephrectomy, prostatectomy) resistent to conservative treatment have been included. Intranodal lymphography using lipiodol and in case of non-responding, further interventions via CT-guided embolisation using lipiodol and/or histoacryl were performed. Succesfull technical outcome was defined as visualization of the lymphatic system and mostly visualisation of a leakage site. Clinically successfull outcome was defined as significant reduction of chylus collected via drainage (>90% reduction) with no significant increase of lymphatic leakage in control exams and an improvement in quality of life (Karnofsky index).

**Results:**
Lymphatic system visualisation was successfull in 100% of patients (50/50). Detection of lymph fistula was successful in X% of patients. The interventions were clinically successfull in 82% of cases (41/50). Reasons for not successful treatment were e.g. treatment for several other diseases and lost to follow up, only 25% reduction of chyle flow, thoracic chyle leakage or chylascites without visualisation of site of leakage. Therapeutic embolisation using lipiodol alone was successfull as a „one-stop-shop“ in 38% of cases (19/50). In 25 patients CT-guided embolisation was performed, 19 were clinically successfull, three were partially successfull and one patient showed only partial improvement, Karnofsky-Index 50->70. Karnofsky-Index improved from 73 to 90 in all patients.

**Conclusion:**
Direct intranodal lymphography is highly recommended for visualisation and therapeutic occlusion of lymphatic leaks. Additional CT-guided embolisation using lipiodol and/or histoacryl improves the clinical outcome in almost all cases.

**Management of Sternal Wound Infection – Determinants of length of stay and recurrence of infection after muscle flap coverage**
ID: 265

**Kategorie: DGCH - Komplikationsmanagement in der Chirurgie**

Juan Liu[1], Kathrin Feller[1]

[1] *Helios Klinikum Krefeld, Krefeld, Deutschland*

**Background:**
The aim of this study was to define determinants of *length of hospital stay (LOS) longer than mean* and *recurrence of infection (ROI) after complete healing* patients with deep sternal wound infections (DSWI).

**Materials and methods:**
In this observational study we included 303 patients (155 females, 148 males, mean age 68 years) treated from 2016 to 2020 at the Department of Plastic Surgery of the HELIOS Klinik Krefeld, Germany. All patients received extensive necrosectomy, repetitive negative pressure treatment periods and final transplantation of a pectoral musculocutaneus flap. In the German DRG-System, the mean inpatient *LOS* depends on the number of surgical procedures and is longer in those with ≥4 surgical procedures (DRG *IO2B*) than with fewer procedures (DRGs *I02C* and *I02D*). The determinants which have a significant effect on LOS longer than mean and *ROI after complete healing* were identified by estimating a logistic regression model. The effect of the different calculated determinants were quantified as Odds ratio. To measure the discriminant ability of the model between patients, we determined a receiver operating characteristic (ROC) curve. The fit of the model was quantified by comparing predicted probabilities of the model with empirical probabilities of the data. The goodness of fit was then measured by applying the Hosmer-Lemeshow test.

**Results:**
Among patients in DRG *IO2B* (n = 246), the variable *clopidogrel and therapeutic anticoagulation* was the most important determinant for a longer *LOS*, with an Odds ratio of 5.83 (95%-CI 0.83/40.80). Female sex and renal insufficiency also prolonged *LOS*. Applying this analyses to the patients with group DRG groups I02C and I02D (n = 57) none of these parameters were predictive. The variable immunosuppression was the most important determinant for *ROI* (n = 49) (OR 4.67; 95%-CI 1.01/21.52). BMI also played a role, but with a much smaller influence.

**Conclusion:**
There are specific risk factors for *LOS longer than mean* and *ROI* in patients with DSWI that can be identified on admission. Addressing these risk factors, if possible, could reduce the rate of patients with *LOS longer than mean* and *ROI*.

**Preoperative respiratory training with incentive spirometry prevents pulmonary complications after open liver surgery - a randomized, controlled pilot trial.**
ID: 355

**Kategorie: DGCH - Komplikationsmanagement in der Chirurgie**

Esther Giehl-Brown[1], Henriette Rangnick[1], Johannes Schweipert[1], Michael Halank[2], Dirk Koschel[2], Jürgen Weitz[1], Carina Riediger[1]

[1]*Universitätsklinikum Carl Gustav Carus, Viszeral-, Thorax- und Gefäßchirurgie (VTG), Dresden, Deutschland*, [2]*Division of Pulmonology, Medical Department I, University Hospital Carl Gustav Carus, Technische Universität Dresden, Dresden, Deutschland*

**Background:**
Postoperative pulmonary complications (PPCs) after major abdominal surgery, especially hepatic surgery, are frequent and associated with considerable morbidity and mortality. Even though preoperative respiratory training is considered as a preventive measurement to reduce the risk of PPCs, there are no valid data available to prove the effect of respiratory training. In the current trial, we analyzed the impact of preoperative respiratory training on the pulmonary function as well as the postoperative outcome in patients undergoing open hepatic surgery.

**Materials and methods:**
This monocentric, randomized controlled pilot trial (RCT) was conducted from August 2020 to July 2022 at the department of Visceral, Thoracic and Vascular surgery at the University Hospital Carl Gustav Carus, Technische Universität Dresden. All patients who underwent open hepatic surgery were screened and after matching with inclusion/exclusion criteria randomized 1:1 to the intervention or control group. The intervention group received preoperative respiratory training by using incentive spirometer with adjustable resistance for 14 consecutive days before surgery. The control group did not receive any preoperative respiratory training. Primary endpoint was PPCs according to the definition of the European Perioperative Clinical Outcome (EPCO). Secondary endpoints were postoperative morbidity and mortality as well as pulmonary function. Pulmonary function was measured by several tests including (ergo)-spirometry at inclusion, 14 days after training before surgery and postoperatively. Exclusion criteria were previous pulmonary surgery, advanced liver cirrhosis or heart failure as well as thromboembolic events in the previous 6 months.

**Results:**
A total of 50 patients were randomized and 41 patients included in the final analysis of this RCT. The indication for liver surgery was malignant disease in the majority of cases (85.7% vs. 95% in the intervention and control group, respectively) and preoperative risk stratification revealed a high risk for PPCs in most patients according to the ARISCAT score (95.2% and 90% in the intervention and control group, respectively). Functional pulmonary tests validated that inspiratory muscle training for 14 consecutive days significantly decreased the residual lung volume (p□0.05) and the total lung capacity (p□0.05) in the intervention group compared to the control group. Importantly, parameters of preoperative ergospirometry revealed no significant differences in cardiopulmonary function between the two groups at time of inclusion. We observed a significantly lower incidence of postoperative pneumonia, pleural effusions and atelectasis in the intervention group in comparison to the control group (p□0.05, p□0.005 and p□0.05, respectively).

**Conclusion:**
Short-term preoperative respiratory training leads to improved pulmonary function with a significant reduction of postoperative pulmonary complications after hepatic surgery in the present pilot trial. An adequately powered RCT has been initiated to prove those findings.

Trial Registration German Clinical Trials Register DRKS0003032

**Surgical management in a case of a sharp-edged ingested foreign body in a 9-month-old infant**
ID: 532

**Kategorie: DGCH - Komplikationsmanagement in der Chirurgie**

Kristina Lisec[1], Oliver Muensterer[1], Jan Gödeke[1]

[1]*Dr. von Haunerschen Kinderspital, LMU München, Kinderchirurgie, München, Deutschland*

**Background:**
Foreign body ingestions are a frequent problem in pediatric emergency departments. The typical age for those ingestions' ranges between 6 months to 3 years old. The nature of the object varies depending on the geographic region and cultural background. Coins are the most common ingested object in Northern America and Europe. Other possible objects include batteries, small toys, screws, hairclips and possibly sharp objects as needles, pins and safety pins.
Up to 80-90% of those foreign bodies coming to medical attention pass uneventfully through the gastrointestinal tract after 4-6 days, 10-20% require an endoscopic removal and less than 1% require an operative intervention.
Over half of foreign bodies are found in the stomach at diagnosis. Depending on the length and thickness of the foreign body in relation to the age of the child in some cases a passage through the pylorus is not possible and removal of the foreign body is usually advised if there is no passage after about 1 week. Sharp and pointed objects are associated with the highest risk of complications, the risk of perforation due to the object increases up to 35%. We describe the unusual case of a successfully managed ingested hairclip that opened up and exposed a sharp edge causing damage to the esophagus.

**Materials and methods:**
A 9-month-old infant presented to our emergency department after ingestion of a 4 cm diameter hairclip configurated in the form of a butterfly. On initial radiographs, the object was found in the stomach. Endoscopic removal was planned after 9 days in case it had not passed distally through the pylorus
During the endoscopic removal, the corroded rivet of the hairclip opened spontaneously in the mid-esophagus, exposing a sharp surface in the area close to the indentation of the aorta While trying to push the object back into the study, two full-thickness lesions of the lower esophagus were noted. The child developed pneumomediastinum and a left sided pneumothorax, which was immediately decompressed using a large-bore intravenous cannula.
In this situation, we opted for a laparoscopic assisted foreign body removal via umbilical single port, placement of a gastrostomy and endoluminal vacuum therapy for treatment of the esophageal lesions.

**Results:**
The described treatment option led to full healing of the esophagus without any mucosal defects or stenosis and without any relevant signs of mediastinitis within days. Full enteral nutrition via the esophagus was achieved after 7 days, and the child was discharged without any symptoms of dysphagia or other complaints on day 14 after severe esophageal trauma. The temporary gastrostomy closed spontaneously after removal of the button at xxx days postoperatively and she remained asymptomatic throughout a 6 month (?) follow-up period.

**Conclusion:**
Inadvertent lower esophageal perforations resulting from sharp esophageal foreign bodies can be treated by temporary minimal-invasive gastric access to remove the object, along with endoluminal vacuum therapy to heal the lesions. Pediatric surgeons should consider this treatment sequence when they encounter similar situations.

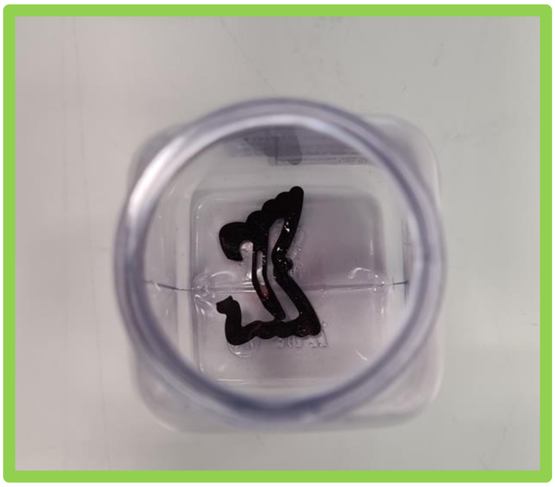

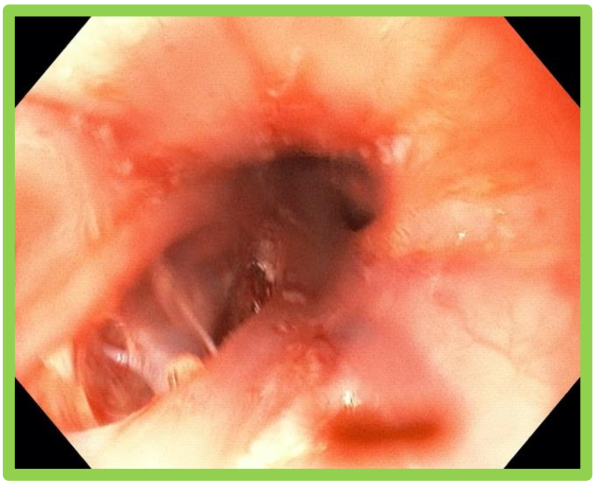

Ingested hariclip

Lesions of the lower esophagus

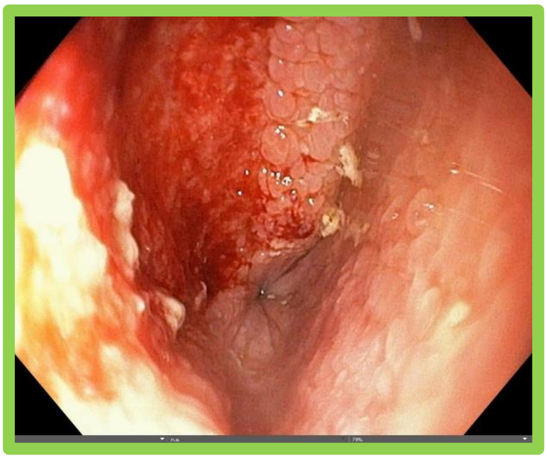

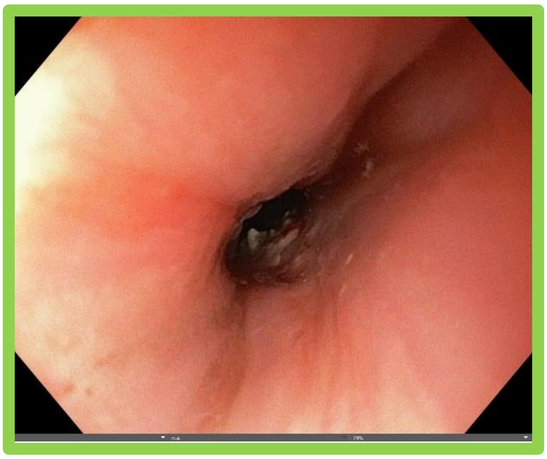

After the endoluminal vacuum therapy

**Bacterial content of the human pancreatic duct and complications after pancreatic resection: an observational study**
ID: 713

**Kategorie: DGCH - Komplikationsmanagement in der Chirurgie**

Elke Demir[1], Bengi Su Yilmaz[1], Carsten Jäger[1], Güralp Ceyhan[2], Helmut Friess[1], Ihsan Ekin Demir[1]

[1]*Klinikum rechts der Isar, Technische Universität München, Klinik und Poliklinik für Chirurgie, MUENCHEN, Deutschland*, [2]*Acibadem University, HPB Surgery, Istanbul, Turkey*

**Background:**
Pancreatic fistula/PF is a challenging surgical complication. We could recently show that intestinal bacteria such as *Enterobacterales* colonize the PF fluid even after a “sterile” operation like distal pancreatectomy/DP. Therefore, we explored the bacterial flora of the human pancreatic duct in a patient collective undergoing pancreatic surgery.

**Materials and methods:**
In this observational study, upon transection of the pancreas during surgery, a swab was inserted into the main duct, and the micro-organismal content was correlated with clinical characteristics.

**Results:**
Between February 2017 and February 2020, an intraoperative swab from the pancreatic duct was obtained from a total of 54 patients who underwent pancreatico-duodenectomy/PD or DP. The swabs were sterile in 39 cases (72.2%), detected intestinal bacteria in 10 cases (18.5%), and other bacteria in 5 cases (9.3%). There was no correlation of the micro-organismal content of the pancreatic duct swab with bacteria detected in the PF fluid or bile. Preoperative ERCP was associated with a higher frequency of bacterial colonization of the pancreatic duct (33.3% vs. 6.7%, $p=0.005$). There was no correlation of the pancreatic duct swabs with postoperative complications.

**Conclusion:**
The human main pancreatic duct is usually sterile. Therefore, the mechanisms leading to infection of PF warrant in-depth, mechanistic investigation.

**Volume Outcome Relationship in Adrenal Surgery from 2009-2017 in Germany**
ID: 776

**Kategorie: DGCH - Komplikationsmanagement in der Chirurgie**

Konstantin Lukas Uttinger[1,2], Joachim Reibetanz[2], Christoph-Thomas Germer[2], Armin Wiegering[2]

[1]*Universitätsklinik Leipzig, Viszeral- Transplantations- Thorax- und Gefäßchirurgie, Leipzig, Deutschland*, [2]*Universitätsklinik Würzburg, Viszeralchirurgie, Würzburg, Deutschland*

**Background:**
Adrenal resections are rare procedures of heterogenous nature. Recent European guidelines advocate a minimum caseload for adrenalectomies (6 per surgeon/year). However, there has been little evidence for a volume outcome relationship in adrenalectomies.

**Materials and Methods:**
Retrospective analysis of adrenal resections of hospital billing data in Germany (2009-2017). Hospitals were grouped into three volume tertiles of approximately equal size. Descriptive, univariate and multivariate analyses were applied to identify possible volume outcome relationship.

**Results:**
17,040 primary adrenal resections were included. Most common diagnoses were benign adrenal tumors (n=8,213, 48.2%), followed by adrenal metastases of extraadrenal malignancies (n=3582, 21.0%) and primary adrenal malignancies (n=1,666, 9.8%). Most frequent surgical procedure was laparoscopic adrenalectomy (n=9,012, 52.9%). 632 low-volume hospitals performed an equal number of resections as 23 high-volume hospitals (median surgeries/hospital/year 3 vs. 31, p<0.001). In 23.1% of patients in low-volume hospitals complications were documented in comparison to 17.3% in high-volume hospitals (p<0.001). Overall in-house mortality was 0.7% (n=126). Age, malignancy, an accompanying resection, complications and open surgery were associated with in-house mortality. In a univariate analysis, surgery in a high-volume hospital was associated with lower in-house mortality (odds ratio 0.47, p<0.001). In a multivariate model, the tendency remained equal (odds ratio 0.59, p=0.104). Regarding failure-to-rescue (defined as death in patients in case of complications), there was a trend to lower mortality in high-volume hospitals.

**Conclusions:**
There is a striking difference in annual caseload of adrenal resections among German hospitals. We provide evidence that surgery in high-volume centers is advantageous for patient outcome, although, fortunately, fatal complications are rare.

**Estimation of optimal Surgical Minimum Caseloads for DKG certified major oncological resections using a DRG billing data based Attributable Risk Fraction Model**
ID: 782

**Kategorie: DGCH - Komplikationsmanagement in der Chirurgie**

Konstantin Lukas Uttinger[1], Armin Wiegering[2]

[1]*Universitätsklinik Leipzig, Viszeral- /Transplantations-/ Thorax- und Gefäßchirurgie, Leipzig, Deutschland*, [2]*Universitätsklinik Würzburg, Viszeralchirurgie, Würzburg, Deutschland*

**Background:**
The establishment of surgical minimum caseloads (SMC) has proven to be an effective tool in centralization programs with the overall aim of optimizing patient care. The German Cancer Society (DKG) certifies hospitals on the basis of SMCs, which have been confirmed to be a benchmark for in-house mortality and failure to rescue if these SMCs are met. However, it has not been analyzed whether or not these current threshold SMCs are optimal with regards to in-house mortality.

**Materials and Methods:**
A statistical modelling on the basis of retrospective anonymized nationwide hospital billing data (DRG data, 2009-2017) was used to estimate optimal SMCs with regards to in-house mortality. Patient record identification was done on the basis of DKG certification criteria. Non-fulfillment of fictitious SMCs (fSMCs) of 5 to 100 resections per year was defined as risk factor in an attributable risk fraction model. The primary endpoint was the attributable death fraction due to fSMC non-fulfillment among total deaths at an optimal fSMC.

**Results:**
There was an overall trend toward lower in-house mortality with rising fSMC in case of fulfillment (7.5% at fSMC=5 and 5.0% at fSMC=50 in pancreatic resections) in all but liver resections. In adjusted approach, the attributable risk of fSMC non-fulfillment accounted for between approx. 20% of total deaths at a threshold in esophageal resections (fSMC=26) and approx. 31% (fSMC=37) in gastric resections. Optimal fSMCs in the adjusted approach were approx. 31 in pancreatic resections. There was a close to linear trend between fSMC fulfillment and the attributable death fraction until up to fSMC=21 in esophageal, fSMC=25 in gastric and fSMC=28 in pancreatic resections. Estimation of optimal fSMC was impossible in liver resections due to a lack of patient volume association.

**Conclusion:**
On the basis of this analysis with its restrictions due to the nature of retrospective data, this work presents optimized minimum caseloads thresholds in specialized surgery with regards to in-house mortality. Most importantly, SMCs should be chosen at higher numbers than the limits of linear trends. Beyond this, this work introduces an approach for optimal SMC determination in procedures with a volume-outcome relationship in general.

**Interdisciplinary evidence based tumor board simulation during surgical term time**
ID: 405

**Kategorie: DGCH - Lehre in der Chirurgie, vom Vorbild bis zur Simulation**

Nora Corinna Altmayer[1]

[1]*Department of General, Visceral and Transplantation Surgery, Heidelberg University Hospital, Heidelberg, Deutschland*

**Background:**
Interdisciplinary activities and scientific competence are becoming increasingly important in the new Medical license Assessment (ÄApprO) 2025. Until now, both were not included in the present curriculum. The practical integration of the topic "Evidence based medicine" (EbM) in the curriculum of surgical training will prepare students for clinical situations in which interdisciplinary or interprofessional discussions are held on a medical topic using current evidence. "Evidence-based medicine (EbM) is the conscientious, explicit, and judicious use of current best evidence in making decisions about the care of individual patients [1]. Including interdisciplinary education and experiences in a curriculum increases students' interdisciplinary communication skills [2]. As studies have shown that students do not feel well prepared for the profession after graduation [3], the "National Competence Based Learning Objectives Course in Medicine" (NKLM) and the new Medical license Assessment (ÄApprO) teach psychomotor and affective skills in addition to cognitive skills. Studies show that multidisciplinary approaches are the best way to provide the complex care that cancer patients need [4]. A positive impact of a higher number of interdisciplinary tumor boards on the clinical outcome are proven [5]. Interdisciplinary tumor boards were associated with decreased mortality over time with a positive influence on patient care by improving survival [6]. Therefore, we implemented the lessons "Evidence based tumor board simulation" so that scientific competence is integrated into the curriculum in an interdisciplinary practical way preparing students more effectively for the profession after graduation and enhancing scientific competences.

**Materials and methods:**
The study was conducted using questionnaires specifically designed for this study, which were handed out before the start and after the end of the teaching unit including a knowledge query and an evaluation. Before the start of the tumor board simulation, a 1-hour lecture on evidence-based medicine was given. The students dealt with real patient cases. After a preparation time with literature research, simulations took place in groups of maximum 10 students played the role of each physician represented in a tumor board (surgeon, internal medicine/oncologist, radiology, radiation medicine, nuclear medicine, pathology). Nearly 200 fourth year medical students participated in the study. The study type was an interventional, quantitative and qualitative cross-sectional study. The quantitative questions were evaluated by a 5-step Likert scale. A p-value $\leq 0.05$ was stated to be significant.

**Results:**
The project "Interdisciplinary evidence-based tumor board simulation during surgical term time" resulted in an increase knowledge outcome regarding learning content. Scientific competence is integrated into the curriculum in a practical way, so that students are prepared for the 12-week scientific work required by the new Medical license Assessment (ÄApprO) 2025. Additionally, soft skills such as team communication were practiced, which is a core competence in the medical profession. Also, students indicated that they had gained interdisciplinary experience that they did not have before. The results also showed that interdisciplinary work has a high value for students within the medical profession and an increase in soft skills quality was shown.

**Conclusion:**
Both scientific competence and soft skills in the sense of interdisciplinary and interprofessional communication are supported by this project in fourth year medical students. Thus, the students are better prepared for different clinical situations independent of the specialty, in which a scientific professional action is required.

1. Sackett, D.L., et al., *Evidence based medicine: What it is and what it isn't - It's about integrating individual clinical expertise and the best external evidence.* British Medical Journal, 1996. **312**(7023): p. 71-72.
2. Chen, A.M.H., et al., *Fostering Interdisciplinary Communication between Pharmacy and Nursing Students.* American Journal of Pharmaceutical Education, 2015. **79**(6).
3. e.V., M.M.F.d.B.D. *Kompetenzbasierte Lernzielkataloge (NKLM, NKLZ) – aus den Fakultäten und für die Fakultäten*. [cited 2022 29.09.2022]; Available from: https://medizinische-fakultaeten.de/themen/studium/nklm-nklz/.
4. Specchia, M.L., et al., *The impact of tumor board on cancer care: evidence from an umbrella review.* BMC Health Serv Res, 2020. **20**(1): p. 73.
5. Freytag, M., et al., *Higher number of multidisciplinary tumor board meetings per case leads to improved clinical outcome.* BMC Cancer, 2020. **20**(1): p. 355.
6. Basendowah, M., et al., *Impact of optional multidisciplinary tumor board meeting on the mortality of patients with gastrointestinal cancer: A retrospective observational study.* Cancer Rep (Hoboken), 2021. **4**(4): p. e1373.

**Minimal-invasive surgery training during universitary medical education: a timely alternative?**
ID: 472

**Kategorie: DGCH - Lehre in der Chirurgie, vom Vorbild bis zur Simulation**

Sophia Jahrstorfer[1], Andreas Lindner[1], Julia Haehl[1], Jan Gödeke[1], Peter Habermeyer[2], Oliver Muensterer[1]

[1]*LMU Klinikum München, Kinderchirurgische Klinik und Poliklinik, München, Deutschland*,
[2]*Schulterzentrum Prof. Habermeyer, München, Deutschland*

**Background:**
Minimal-invasive surgery (MIS) training usually starts at the beginning of postgraduate training. A potential challenge to effective skill development may pose the high workload associated with the first years of surgical training. A possible solution to this problem may be the shifting of basic minimal-invasive surgical skill development into the medical school curriculum. However, there is still considerable controversy on the best timing and sequence of practical MIS training for students. In this study, we compared different approaches to medical school MIS training, including the sequence and timing of training.

**Materials and methods:**
After successfully establishing a basic MIS practical curriculum termed SuSiPed 1.0, we developed a more advanced training program by the name of SuSiPed2.0. This program consisting of six individual training modules: stitching (Fig.1), continuous stitching (Fig.2), 3D-knot-tying (Fig.3), general knot-tying (Fig.4), and the simulation of a fundoplication (Fig.5) and esophageal anastomosis (Fig.6) using slipknots. All trainees repeated the curriculum three times to characterize a learning curve. Time to completion was measured as the primary outcome parameter. Errors committed during completion of the modules were considered secondary parameters.
Instructions for accomplishing the tasks were given by video tutorial prior to the hands-on training. To analyse the subjective relevance of attending MIS training and the amount of money for participating at the three appointments the subjects completed a survey.
Medical students were recruited as study participants and divided into three groups:
Group 1 attended all three training appointments within 14 days. The second group (group 2) completed the three appointments in more than 15 days. Group 1 and 2 started their training with SuSiPed 2.0, without previous basic training. Group 3 had completed basic MIS simulation training (SuSiPed1.0) a year earlier and completed SuSiPed 2.0 in the context of this study within 25 days.
We compared the time needed for absolving the whole curriculum at the third appointment of each group and evaluated the students' subjective appreciation for the SuSiPed 2.0 program in a Likert point scale from one to six. Subjects were also asked how much they would pay out-of-pocket for participating in such SuSiPed2.0 in euro.
Statistical analysis was conducted with the Welsch's T-test.

**Results:**
A total of 44 participants was recruited (19 in group 1, 20 in group 2, 5 in group 3). The study reveals a significant difference between the third and the other groups and no difference between the first and the second group.
Group 1 needed 1675s±497 (n=19) versus 1225s±153 for group 3 (n=5) $p<0.003$
Group 2 needed 1896s±715 (n=20) versus 1225s±153 for group 3 (n=5) $p<0.001$
Group 1 needed 1675s±497 (n=19) versus 1896s±715 for group 2 (n=20) $p>0.27$
Regarding the questionnaires most students (n=42) ranked the general relevance of laparoscopic education for their personal skills on an average of 5.7 in a scale from 1 (irrelevant) to 6 (very relevant). Taking an economic value into consideration there can be indicated the willingness of paying around 74.17 euro for the three appointments.

**Conclusion:**
Our study shows that prior completion of a basic simulation training improved performance in a subsequent advanced MIS training program. The timing itself in terms of intervals between sessions played no significant role. Including basic MIS surgical simulation training early on during medical school may be useful for later surgical skill development. The value of this model is underscored by the subjective feedback of the students and the fact that they would be hypothetically willing to pay a substantial amount out-of-pocket for such training during their medical studies.

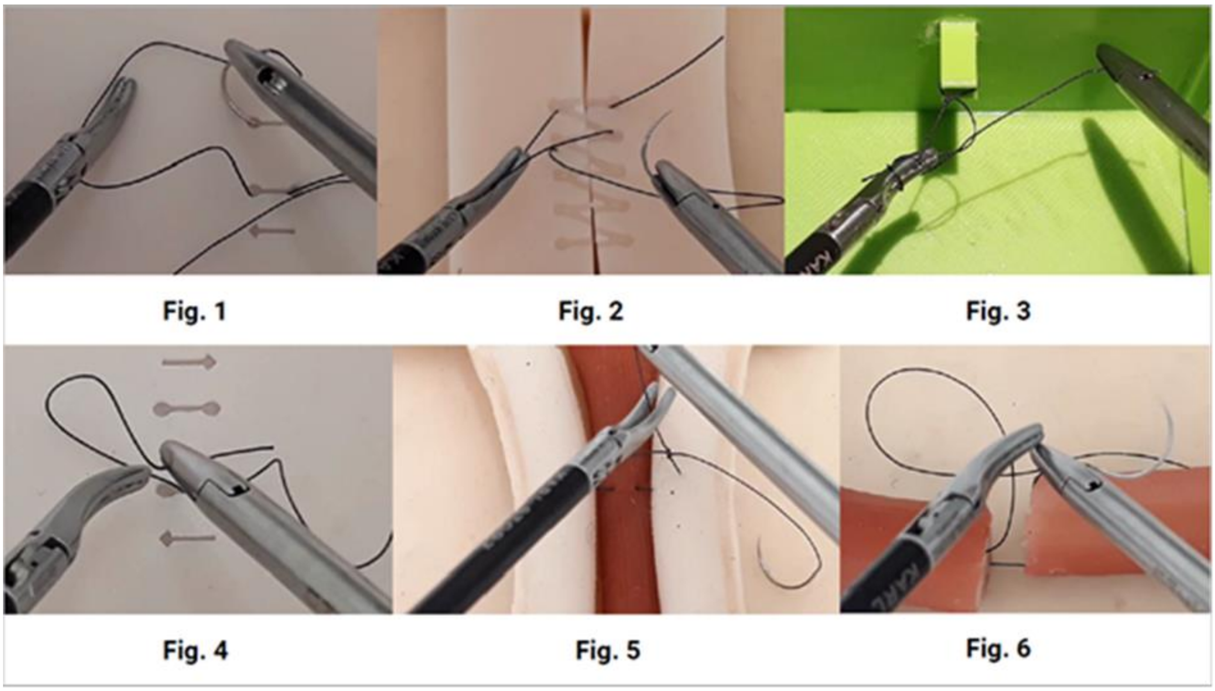

Fig. 1 Fig. 2 Fig. 3
Fig. 4 Fig. 5 Fig. 6

**Changing Reality – augmented and virtual Reality in neurosurgery as a teaching tool for medical students under the surge of COVID-19**
ID: 562

**Kategorie: DGCH - Lehre in der Chirurgie, vom Vorbild bis zur Simulation**

Rubén Silvero Isidre[1], Michael Müther[1], Hendrik Friederichs[2,3], Walter Stummer[1], Markus Holling[1]

[1]*Department of Neurosurgery, University Hospital Münster, Münster, Deutschland*, [2]*AG Medical Education, University Bielefeld, Bielefeld, Deutschland*, [3]*Institut für Ausbildung und Studienangelegenheiten (IfAS), University Münster, Münster, Deutschland*

**Background:**
The COVID-19 pandemic led to more limited exposure to surgical subspecialties such as neurosurgery and consequently restricted medical insight into the specialty. There is, therefore, a need for a high-quality educational experience to promote undergraduate neurosurgical education. Augmented (AR) and Virtual Reality (VR) technology are promising teaching tools. For example, it has been commonly used in neurosurgery to develop technical competencies, such as surgical skills, or to conceptualize complex 3D anatomic relationships. Another essential aspect that favors utilizing this technology is that learners find this technology motivating, engaging, and entertaining for learning purposes. Hence, we incorporated Augmented Reality (AR) and cloud rendering Virtual Reality (VR) methods into the curriculum to ensure patient safety while teaching patient-specific aspects with real-time 3D image reconstruction. This study aims to identify whether this teaching intervention within an undergraduate neurosurgical workshop setting could improve the course rating.

**Materials and methods:**
The study was conducted from January 2019 to June 2022. The workshop for medical students is conducted yearly. It is divided into two parts. First, the medical students are trained in the basics of systematic neurosurgical/neurological examinations and learn about the most common neurological diseases. Then, in the second part, exercises with neurosurgical instruments, an endoscope, and microscopes are carried out on various simulation models in our Skills Lab.
Unlike the conventional group, participants in the intervention group could additionally virtually interact with 3D models using AR (Magic Leap One) and VR (Occulus quest 2) and plan the surgical intervention. The students evaluated the teaching experience through a Likert-scale questionnaire using the standardized, anonymous online tool EVALuna (1 for the best and 100 for the worst evaluation).

**Results:**
Comparison of course participants' ratings showed improvement in the course evaluation from 11,73 ± 13,3 (control group, n = 120) to 6,78 ± 7,4 points (intervention group, n = 103). The Welch's t-test concluded that the difference between groups were statistically significant [T-Welch (192,16) =3,5; $p \leq 0.001$] with moderate effect size (Hedges' g=0,46, 95% CI [0.20, 0.72]).

**Conclusion:**
AR and VR may provide an effective complementary tool to expose medical students to neurosurgery. In addition, the feedback from the medical students indicates a high acceptance of this new teaching format and encourages the adoption of disruptive technology into medical school curricula.

**The Learning Curve for Ivor Lewis Minimally Invasive Esophagectomy – a single high volume centre experience.**
ID: 619

**Kategorie: DGCH - Lehre in der Chirurgie, vom Vorbild bis zur Simulation**

Philippa Seika[1], Jonas Raakow[1], Andreas Brandl[1], Eva Maria Dobrindt[1], Max Magnus Maurer[1], Ramin Raul Ossami Saidy[1], Matthias Biebl[2], Johann Pratschke[1], Christian Denecke[1]

[1]*Charité Universitätsmedizin Berlin, Chirurgische Klinik, Berlin, Deutschland*, [2]*Ordensklinikum Linz, Department of Surgery, Linz, Österreich*

**Background:**
Minimally Invasive Esophagectomy (MIE) has been established as the standard surgical therapy for patients with esophageal cancer (GC) at our institution. While vital for improving patient outcomes, surgical innovation and pioneering may often be accompanied with learning curves. Evidence-based training and safe implementation programs are essential to avoid implementation-related morbidity.

**Materials and methods:**
From 2010 to 2020, 174 consecutive patients who received MIE for primary esophageal malignancy at our institution were included. Using varying-coefficient logistic regression models, risk factors for perioperative morbidity and mortality were evaluated. This model was then used to establish a risk-adjusted cumulative sum model (RA-CUSUM) to examine surgical outcomes. The patients were arranged according to the procedure number and separated into training and competent group based on perioperative outcome and operating time.

**Results:**
A total of 174 MIE with thoracic anastomosis were performed. Forty-nine cases were required for the completion of the learning curve in terms of perioperative outcome while 103 cases were required in terms of operating time. MIE performed after a learning curve was associated with a shorter median operating time (OT) (458 min vs. 434 min, 0.033). There was no difference in length of stay (A = 22 days vs. B= 18 days) (p=0.333) or anastomotic leakage rates between the two groups (group A vs. group B, 8(16.3%) vs. 15(11.8%) p=0.753). Furthermore, no differences were seen in terms of quality criteria such as R0 resection (p=0.418) and perioperative mortality between the groups (p=0.198). Upon multivariate analysis, no differences were seen in overall survival (OS) and disease-free survival (DFS).

**Conclusion:**
For experienced laparoscopic surgeons to attain technical competence in MIE, a minimum of 49 cases are necessary given extensive prior laparoscopic experience. Surgical training in MIE is possible under the preservation of oncological outcome for fellows with extensive prior laparoscopic experience.

**Surgical OSCE before, during and after COVID-19 pandemic**
ID: 918

**Kategorie: DGCH - Lehre in der Chirurgie, vom Vorbild bis zur Simulation**

Giulia Manzini[1], Maximilian Denzinger[2], Marko Kornmann[2], Ian Hines[3], Michael Kremer[1]

[1]*KSA Kantonsspital Aarau, Aarau, Schweiz*, [2]*Uniklinik Ulm Abt Chirurgie, Ulm, Deutschland*, [3]*East Carolina University, Greenville, United States*

**Background:**
The Objective Structured Clinical Examination (OSCE) was introduced by Harden et al. (1975) as an instrument to assess clinical, theoretical, and practical competencies. The COVID-19 pandemic had a significant impact on medical education, especially in the area of practical clerkships of medical students where in person, hands-on, instruction was replaced by online seminars. Aim is to assess if the pandemic has influenced medical student performance on the surgical OSCE.

**Materials and methods:**
Student performance at a German University Hospital during three surgical OSCE examination windows, winter semester 2018/2019, 2020/2021 and 2021/2022, corresponding to before, during, and after pandemic changes to instruction were compared. For this comparison, 3 out of the 12 total OSCE stations were considered: suturing (0 to 20 points scale), determination of visceral surgical cases with clinical abdominal examination (0 to 10 points), and visceral description of surgical operation technics (0 to 8 points). The marks achieved by the students were compared among the three groups above using the Student t-test for independent samples. Evaluators at both visceral surgical stations were the same during the three OSCE and are senior consultants in visceral surgery. Evaluators of suturing were older students which changed at each OSCE.

**Results:**
A total of 423 students took part in one of the three OSCE examination; 125 students were examined in winter semester 2018/2019, 155 in winter semester 2020/2021 and 143 students in winter semester 2021/2022. The pandemic-impacted group had a higher average score than the group before pandemic (170.52 vs. 157.43) as well as the group after pandemic (170.52 vs. 168.60). Student performance in suturing was better during the pandemic than before pandemic pandemic (19.59 (± 0.95) vs 17.00 (± 2.04), p<0.001) as well as during pandemic than after pandemic (19.59 (± 0.95) vs 18.25 (± 1.87), p<0.001). Student performance in theoretical solving of a clinical case and performance of a complete abdominal examination was better before the COVID-19 pandemic than during the pandemic (8.32 (± 1.37) vs 7.94 (± 1.61), p=0,04) as well as after pandemic than during pandemic (8.36 (± 1.37) vs 7.94 (± 1.61), p=0.02). No difference was found in the comparison of the performance before and after pandemic (8.32 (± 1.37) vs 8.36 (± 1.37), p=0.83). Students` performance in surgical technique was not different both before pandemic versus pandemic (p=0.37) and during pandemic versus after pandemic (p=0.14).

**Conclusion:**
Cessation of the practical, in person, hands-on internship reduced student performance on the OSCE clinical case and abdominal examination section but not on suturing or surgical technique sections. These data suggest that alternative approaches to instruction including online education may be sufficient for some but not all aspects of the surgical internship. Further research is needed to understand how other competencies may have been affected during the pandemic and how instruction methodologies may be amended in the future to better serve student success.

**Validation of the international classification for grading the resectability of the PDAC with regard to its prognostic accuracy.**
ID: 346

**Kategorie: DGCH - Onkologische Chirurgie im interdisziplinären Kontext**

Alexander Genchev[1], Ulf Neumann[1], Tom Florian Ulmer[1], Sven Arke Lang[1], Georg Wiltberger[1]

[1]*Uniklinik Aachen, Aachen, Deutschland*

**Background:**
The 2017 international consensus criteria (ICC) expanded the definition of anatomically (A) borderline resectable (BR) pancreatic ductal adenocarcinoma (PDAC) to include both a biological (B) and a conditional (C) dimension. To date, the prognostic accuracy in relation to the individual groups has only been insufficiently investigated in this new classification. Anatomical factors are determined by the tumor contact with the main visceral blood vessels. BR-B are patients with preoperative CA19-9 values greater than 500 U/ml. BR-C is defined as a preoperative Eastern Cooperative Oncology Group (ECOG) status ≥2.)

**Materials and methods:**
In the period 2010-2021, a total of 378 patients who underwent pancreatic resection due to adenocarcinoma were included retrospectively in the study. They were divided into 12 groups according to the international consensus classification with all possible combinations of the different variables: Resectable (R)/BR/LA-A; R/BR-B; R/BR-C. Group 1 (n = 227) - R-ABC consisted of patients who appeared resectable in all 3 variables. Group 2 (n = 41) were patients with R-AC, but BR-B. Group 3 (n = 29) consisted of R-AB, but BR-B. Group 4 (n = 6) consisted of R-A and BR-BC. Group 5 (n = 37): BR-A, R-BC. Group 6 (n = 7): BR-AB, R-C. Group 7 (n = 1): BR-AC, R-B. Group 8 (n = 1): BR in all 3 components. Group 9 (n = 20): LA-A, R-BC. Group 10 (n = 6): LA-A, BR-B, R-C. Group 11 (n = 3): LA-A, BR-C, R-B. Group 12 - LA-A, BR-BC could not be build.
Differences in overall survival (OS) and disease-free survival (DFS) were examined using Kaplan-Maier curves for the entire troop and for the subgroups. An analysis of the perioperative morbidity and mortality as well as the long-term outcome was carried out. A univariate analysis was performed to investigate the significance with whom the different variables influence the general outcome.

**Results:**
A total of 378 patients underwent oncological resection. Median age of the total cohort was 67.2 (20-86 years), 200 male and 180 female. R-A were 304 patients, R-B - 315 and R-C - 339. The median OS (mOS) was 25 months and disease-free survival (DFS) was 9 months for the entire force. There was a significant difference between the subgroups with reduced mOS and mDFS in the groups involving patients with BR-B or -C variable, showing consistently a worsened oncological outcome in comparison to those with resectable B/C condition despite having the same anatomical tumor relation. Interestingly, LA-A groups had a similar outcome compared to ones, consisting of patients with anatomically less advanced PDAC, showing that well selected patients, which used to be called irresectable, could be operated and possibly cured after appropriate pre-treatment. Furthermore, presented patients with highly elevated CA 19-9 levels worsened long time oncological outcome, showing that elevated tumor markers should also be considered, when planning the best therapeutic approach before surgery.

**Conclusion:**
The preoperative classification into anatomical as well as biological and conditional resectability criteria has a high prognostic accuracy. The international consensus criteria for resectability should be taken into account when planning the individual therapy for the patient. Further studies are necessary to validate the new criteria in prospective studies.

### Prognostic value of metastatic lymph node ratio for patients undergoing curative colon cancer resection

ID: 422

**Kategorie: DGCH - Onkologische Chirurgie im interdisziplinären Kontext**

Pawel Mroczkowski[1,2], Samuel Kim[2], Rony Otto[2], Hans Lippert[2], Olof Jannasch[2]

[1]*Univeritätsklinikum Knappschaftskrankenhaus , Bochum, Deutschland*, [2]*An-Instutut für Qualitätssicherung in der operativen Medizin GmbH, Otto-von-Guericke- Universität, Magdeburg, Deutschland*

**Background:**

Due to the impact of nodal metastasis on colon cancer prognosis, accurate lymph node evaluation is required. Prognosis of nodepositive patients is not homogenous within TNM stages, so additional parameters are needed. The ratio of metastatic to examined nodes (LNR) may have additional prognostic value to the actual staging system. The study analyses the identification of influencing factors of a high lymph node yield (LNY) and its impact on survival. The LNR is evaluated in patients with fewer than 12 or at least 12 evaluated nodes

**Materials and methods:**

From 01.01.2008 to 31.12.2012 7012 patients after radical resected colon cancer in UICC stage II and III were included. For LNR analysis (cut-offs: 0,05; 0,2 and 0,4) nodepositive patients were included and stratified in <12 and >12 evaluated nodes.

**Results:**

In 94,5% at least 12 nodes were evaluated showing an improved 5-years-OS (68% vs. 63%, p=0,027). Patients with at least 12 evaluated nodes were younger, had a lower ASA classification, high pT and pN stage and often a right sided tumour. LNR was identified as an independent factor correlating with overall survival with hazard ratios of 1,016 to 2,698 for patients with less than 12 and HR of 1,248 to 3,615 for those with at least 12 nodes. The LNR and pN stage were independent prognostic factors in both cohorts (<12 and ≥12 nodes). Comparing the HR and OS, the LNR in patients with ≥ 12 lymph nodes appears to be superior to the pN stage: HR (LNR 4) =3,615 vs. HR (pN2) =1,832, while OS (LNR 4) = 33,1% vs. OS (pN2) = 49,0%. LNR better predicts overall survival than the pN stage, when at least 12 nodes are evaluated.

**Conclusion:**

Metastatic LNR is an independent predictor for survival. In patients with ≥12 evaluated nodes the LNR delivers additional prognostic information.

**Proteinkinase-inhibition augments the response against multidrug chemotherapy in pancreatic cancer**
ID: 508

**Kategorie: DGCH - Onkologische Chirurgie im interdisziplinären Kontext**

Danilo Hackner[1], Siyuan Zeng[1], Hai Yang[1], Eva Lentsch[1], Maximilian Brunner[1], Isabella Dörflein[1], Susanne Pfeffer[1], Robert Gruetzmann[1], Christian Pilarsky[1]

[1]*University Hospital Erlangen, Department of Surgery, Erlangen, Deutschland*

**Background:**
Pancreatic ductal adenocarcinoma (PDAC) is worldwide the 7$^{th}$ common cause of cancer related deaths with 4.6% of all cancer mortalities worldwide [1, 2]. Due to its aggressive nature and silent character, only leading to symptoms rather late in the progression of the disease, most of the patients are in advanced stages when diagnosed. In recent years, only moderate and not really successful results have been achieved in causal therapy. Hence, surgical therapy in the form of pancreatoduodenectomy (PD) is still the only curative procedure. PD is whenever possible combined with an adjuvant chemotherapy, in palliative situation chemotherapy is the only treatment route. However, there are commonly patients which show only little to non-response to the different administered chemotherapy-schemes [3, 4]. The dysregulation of protein kinases is associated with numerous carcinogenic processes and furthermore chemoresistance [5, 6]. Thus, there is a huge need to explore the molecular mechanisms which cause chemotherapy-resistance and find strategies to implement these findings in clinical practice.

**Materials and methods:**
Pancreatic cancer cells were transduced with a protein kinase library followed by continuous exposure to vehicle control or oxaliplatin (IC 90) chemotherapy for 21 days. Based on CISPR Cas9 knockout screening and next-generation sequencing as well as MAGeCK-VISPR analysis we identified candidate genes. We used CRISPR-Cas9 to knock out the target gene, and verified the screening hits for overcoming chemoresistance through in vivo and in vitro experiments.

**Results:**
We identified several cell cycle checkpoint kinases and DNA damage-related kinases as targets for overcoming chemoresistance, including $MAP_3K_7$, which encodes for a protein of the serine/threonine protein kinase family. We state, that loss of function of $MAP_3K_7$ makes PDAC cells sensitive to chemotherapy with oxaliplatin. In preliminary work we already showed, that the targeted inhibition of Cyclin dependent kinase 7 (CDK7) made PDAC cells sensitive to drug combination chemotherapy [7].

**Conclusion:**
Our results suggest a new therapeutic combination for PDAC, and support the application of CRISPR-Cas9-based functional screening in identifying novel therapeutic targets to potentiate existing cancer therapies.

**References:**
1. Huang J, Lok V, Ngai CH et al. Worldwide burden of, risk factors for, and trends in pancreatic cancer. Gastroenterology 2021; 160: 744-754
2. Lippi G, Mattiuzzi C. The global burden of pancreatic cancer. Arch Med Sci 2020; 16: 820-824. doi:10.5114/aoms.2020.94845
3. Zeng S, Pöttler M, Lan B et al. Chemoresistance in pancreatic cancer. International journal of molecular sciences 2019; 20: 4504
4. Sarvepalli D, Rashid MU, Rahman AU, et al. Gemcitabine: A Review of Chemoresistance in Pancreatic Cancer. Crit Rev Oncog. 2019;24(2):199-212

5. Liu R, Chen Y, Liu G et al. PI3K/AKT pathway as a key link modulates the multidrug resistance of cancers. Cell death & disease 2020; 11: 1-12
6. Uppada SB, Gowrikumar S, Ahmad R et al. MASTL induces Colon Cancer progression and Chemoresistance by promoting Wnt/β-catenin signaling. Molecular cancer 2018; 17: 1-15
7. Zeng S, Lan B, Ren X et al. CDK7 inhibition augments response to multidrug chemotherapy in pancreatic cancer. J Exp Clin Cancer Res. 2022 Aug 10;41(1):241.

### Analysis of peripheral immune events in colorectal cancer

ID: 647

**Kategorie: DGCH - Onkologische Chirurgie im interdisziplinären Kontext**

Can Lu[1], Viktor H von Ehrlich-Treuenstätt[1], Josephine Schardey[1], Ulrich Wirth[1], Jens Neumann[1], Clemens Gießen-Jung[1], Jens Werner[1], Alexandr V Bazhin[1], Florian Kühn[1]

[1]*LMU Klinikum München, München, Deutschland*

**Background:**
The interaction between tumor and immune cells is known to affect the progression of colorectal cancer (CRC), but the effect of CRC cells on systemic immunity remains unclear. We aimed to comprehensively evaluate circulating immune subsets and gene expression analysis of CRC patients.

**Materials and methods:**
Circulating immune subsets were utilized to construct a diagnostic model. The subset composition and phenotype of B and T lymphocytes, neutrophils, monocytes, dendritic cells (DCs), myeloid-derived suppressor cells (MDSCs), NK, and NKT cells were investigated in peripheral blood samples from 12 CRC patients and 11 healthy controls by multicolor flow cytometry. Furthermore, we performed bioinformatics analyses to obtain the differential expression genes (DEGs) and immune cells between CRC patients and normal controls. Correlation analysis was conducted to discover regulatory genes and clinical test parameters associated with the distribution of circulating immune subsets.

**Results:**
In contrast to healthy controls, CRC patients had a reduced proportion of B and T lymphocytes, T helper (Th) cells, non-classical monocytes, DCs, and an increased proportion of polymorphonuclear MDSCs, as well as reduced expression of CD69 on both $CD56^{dim}$ and $CD56^{bright}$ NK cells. A diagnostic model integrating seven immune subsets was constructed to discriminate CRC patients and healthy controls with an AUC of 1.000 (95% CI 1.000-1.000). Moreover, NR3C2, CAMK4, and TRAT1 were identified as candidate genes regulating the number of circulating Th cells in CRC patients. Right-sided CRC patients had a similar systemic immune landscape to left-sided ones. In addition, we found a robust positive correlation between immune subsets and three clinical test parameters, including gamma-glutamyltransferase, aspartate aminotransferase, and alanine aminotransferase.

**Conclusion:**
The immune suppression of systemic immune responses is evident in CRC patients. The altered composition of circulating immune subsets in CRC could complement the regional immune status of the tumor microenvironment and contribute to the discovery of immune-related biomarkers for the diagnosis of CRC.

**MACC1 as a novel biomarker for early recurrence and survival in pancreatic ductal adenocarcinoma (PDAC) patients**
ID: 712

**Kategorie: DGCH - Onkologische Chirurgie im interdisziplinären Kontext**

Andrew Moufarrej[1,2], Alexander Genchev[1], Phillip Rum[3], Tom Florian Ulmer[1], Sven Arke Lang[1], Jan Bednarsch[1], Andreas Lambertz[1], Ulf Neumann[1], Anne Andert[1], Georg Wiltberger[1]

[1]*Klinik für Allgemein-, Viszeral- und Transplantationschirurgie , Uniklinik Aachen, Aachen, Deutschland*, [2]*Klinik für Dermatologie, Uniklinik Düsseldorf, Düsseldorf, Deutschland*, [3]*RWTH Aachen, Aachen, Deutschland*

**Background:**
Pancreatic ductal adenocarcinoma (PDAC) is linked to a dismal prognosis with a 5-year survival of ~10%. Many patients receiving surgical resection recur early after resection, hampering overall and disease-free survival. Current risk stratification strategies are insufficient to determine patient subgroups at high risk for early recurrence. Metastasis-Associated in Colon Cancer 1 (MACC1) has been shown to be a promising molecular marker to predict postoperative cancer recurrence and metastasis in several solid tumor entities. We aim to measure MACC1 plasma and tissue expression in PDAC patients to determine its potential as a biomarker for early recurrence and survival.

**Materials and methods:**
All PDAC patients treated at the RWTH Aachen University Clinic were assessed for inclusion. Plasma samples were obtained prior to surgical resection or chemotherapy. MACC1 plasma expression was determined by enzyme-linked immunosorbent assay. MACC1 tissue expression was evaluated by immunohistochemistry. MACC1 expression was compared between PDAC patients and healthy controls. A survival analysis and cox regression analysis was performed to determine the predictive value of MACC1 expression on overall and disease-free survival.

**Results:**
Plasma samples were obtained from 118 PDAC patients and 30 controls, whereas tissue samples were available from 258 PDAC patients. MACC1 plasma concentrations were significantly higher in patients with stage III/IV disease (Median 17.8 ng/ml (IQR 9.2)) if compared to stage I/II (Median 12.3 ng/ml (IQR 11.0) ($p = 0.005$). All stages had higher MACC1 plasma expressions if compared to controls. A high MACC1 plasma expression was an independent predictor for disease-free survival, whereas a high MACC1 tissue expression predicted overall survival in PDAC patients.

**Conclusion:**
MACC1 is highly expressed in PDAC tissue and patient plasma, with high MACC1 plasma concentrations correlating to advanced disease. Moreover, we for the first time reveal, that high MACC1 expression predicts early recurrence and worse survival in PDAC patients.

### Disease progression during multimodal two-staged hepatectomy for treatment of bilobar colorectal liver metastases

ID: 771

**Kategorie: DGCH - Onkologische Chirurgie im interdisziplinären Kontext**

Simon Moosburner[1,2], Annika Kurreck[3], Ioanna Pouyiourou[3], Nora Nevermann[4], Dominik Geisel[5], Uli Fehrenbach[5], Felix Krenzien[1,2], Sebastian Knitter[1], Hannes Freitag[1], Moritz Blank[1], Wenzel Schöning[1], Georg Lurje[1], Moritz Schmelzle[4], Igor Sauer[1], Dominik Modest[3], Johann Pratschke[1], Nathanael Raschzok[1,2]

[1]*Charité – Universitätsmedizin Berlin, corporate member of Freie Universität Berlin and Humboldt-Universität zu Berlin, Department of Surgery Campus Charité Mitte | Campus Virchow-Klinikum, Berlin, Deutschland*, [2]*Berlin Institute of Health at Charité – Universitätsmedizin Berlin, BIH Academy, Clinician Scientist Program, Berlin, Deutschland*, [3]*Charité – Universitätsmedizin Berlin, corporate member of Freie Universität Berlin and Humboldt-Universität zu Berlin, Department of Hematology, Oncology, and Tumor Immunology, Berlin, Deutschland*, [4]*Hannover Medical School, Clinic for General, Visceral and Transplant Surgery, Hannover, Deutschland*, [5]*Charité – Universitätsmedizin Berlin, corporate member of Freie Universität Berlin and Humboldt-Universität zu Berlin, Department of Radiology, Berlin, Deutschland*

**Background:**
Both patients with synchronous and metachronous colorectal liver metastases (CRLM) benefit from local treatment. If bilobar CRLM cannot be treated with one single procedure due to bilobar involvement and/or insufficient tumor-free future liver remnant (FLR), a multimodal two-staged hemi-hepatectomy (TSH) can be considered. TSH may consist of surgical and/or interventional clearance of the FLR followed by hypertrophy induction by portal venous embolization (PVE), and consecutive final resection However, tumor growth during the hypertrophy phase may affect the resectability of metastases. We aimed to analyze the rate of disease progression during the hypertrophy phase of TSH with PVE in patients with bilobar CRLM.

**Materials and methods:**
Patients with CRLM surgically treated between 2019 and 2021 at the Charité – Universitätsmedizin Berlin, Campus Charité Mitte and Campus Virchow Klinikum, were included in this retrospective analysis. Patients undergoing TSH received a computed tomography (CT) prior to the initial resection and 6 weeks after PVE prior to the final resection. The respective CT scans were assessed by means of response evaluation criteria in solid tumors (RECIST) version 1.1. Patient characteristics, information on local treatment procedures, and follow-up data were retrospectively retrieved from the electronic health records.

**Results:**
Between 2019 and 2021 TSH was planned for 51 patients, who received PVE prior to the intended second hepatectomy. Of these, 24 47% had evidence of progressive disease (PD) following PVE after the first stage resection, i.e., growth of pre-existing CRLM or occurrence of new metastases. In 9 cases of these 24 (38%), the intended second resection was not performed (n=7 intrahepatic PD, n=1 extrahepatic PD, n=1 insufficient liver hypertrophy). The 1-year overall survival was 89%, and the 1-year recurrence-free survival was 35%.

**Conclusion:**
The significant rate of intrahepatic disease progression during the hypertrophy interval in patients undergoing TSH with PVE suggest that a hypertrophy interval of six weeks might be a critical limitation of multi-stage surgical strategies. This result supports innovative treatment strategies with the objective to increase disease control in this treatment algorithm.

**Locally advanced rectal cancer (LARC): Tailoring preoperative treatment according to tumor location – is there any benefit?**
ID: 871

**Kategorie: DGCH - Onkologische Chirurgie im interdisziplinären Kontext**

Recca Talaulicar[1], Andreas Leha[2], Julie Schanz[3], Johanna Kreutzer[1], Timotheus Ritter[1], Philipp Schüler[1], Tatiana Grammatikopoulou[1], Torsten Liersch[1]

[1]*Klinik für Allgemein-, Viszeral- und Kinderchirurgie - Universitätsmedizin Göttingen, Göttingen, Deutschland*, [2]*Institut für Medizinische Statistik - Universitätsmedizin Göttingen, Göttingen, Deutschland*, [3]*Klinik für Hämatologie und Medizinische Onkologie - Universitätsmedizin Göttingen, Göttingen, Deutschland*

**Background:**
Preoperative 5-fluorouracil (5-FU)-based chemoradiotherapy (CRT ± oxaliplatin, OX) for locally advanced adenocarcinoma of the rectum (LARC; stages II and III) is expected to improve resectability, sphincter-preservation and survival. Compared with surgery alone (Heald et al. 2017), synergistic effects of multimodal treatment (MMT, pre-/postoperatively) on prognosis are controversial. For stage II / III cancers in the upper rectum (≥ 12 cm from anal verge), the benefit of preoperative MMT is under debate. In this single site study, we evaluated the efficacy of preopCRT ± OX followed by total mesorectal excision (TME) and adjuvant CTx vs. upfront surgery in patients with LARC (< 16 cm from anal verge).

**Materials and methods:**
Between 1998 and 2013, 402 patients (m: 67.2%, f: 32.8%; median age: 64 years) underwent MMT according to the protocols of the CAO/ARO/AIO-94, -04 and GAST-05 (ISRCTN35198481) trials of the German Rectal Cancer Study Group. LARC was present in the lower (33.8%, < 6 cm from anal verge), middle (33.3%, ≥ 6 cm to < 12 cm), and upper (32.9%, ≥ 12 cm) rectum as clinical stages II / III in 28.4% and 68.7%, respectively. Anterior (ARR; PME), low anterior (LAR; TME or PME) and abdominoperineal resections (APR; TME) were performed in 9.0%, 68.6% and 22.1%, respectively. R0 and negative CRM status (circumferential resection margin, ≥ 1 mm) were achieved in 98.5% and in 88.8%, while optimal quality of the specimen was confirmed by the pathologist in 77.8%. The therapeutic efficacy was assessed by using logrank tests, Kaplan-Meier estimators and uni-/multivariable survival models. Cox proportional hazards regression models were fitted for risk factors and survival endpoints, adjusted for risk factors, pretreatment, tumor location, their 2-way interactions, and the treatment regimen.

**Results:**
After upfront surgery (n = 180), stages I to IV were noted in 13.9%, 40.0%, 41.7%, and 4.4%, respectively, and CRM positivity in 30.3%. After preoperative CRT (n = 222), ypUICC stages 0 to IV were present in 14.9%, 19.4%, 33.6%, 33.1%, and 5.7%, respectively. CRM positivity was identified in only 7.2%. During 91-months (median) follow-up, 119 recurrences were diagnosed in 30.0% of patients; of these, local recurrences were detected in 1% (alone) and 5.3% (with distant metastases). Paired contrast tests for OS showed a favorable effect of preopCRT on stages ≤ II by downsizing / -staging (HR: 0.37; p < 0.001). Similarly, preopCRT showed a positive effect for stages ≤ II in the upper rectum (HR: 0.16; p = 0.004). After upfront surgery, OS was also better in stages ≤ II vs. ≥ III (HR: 0.37; p = 0.018). For DFS, there was an effect of preopCRT in favor of stages ≤ II (HR: 0.37; p < 0.001). In CSS, preopCRT was beneficial for stages ≤ II (HR: 0.22; p < 0.001) in the lower and mid rectum and for carcinomas in the upper third (HR: 0.0096; p = 0.001). In this tumor location, patients with stages ≤ II vs. ≥ III *per se* had better CSS (HR: 0.029; p = 0.007); thus, the actual benefit of preopCRT remains questionable.

**Conclusion:**
Preoperative MMT resulted in better survival for patients with stages ≤ II, especially for cancers in the mid and lower rectum (< 12 cm). Patients with residual stages ≥ III were identified as "non-responders". For patients with upper rectal cancer, there was no disadvantage in stages ≤ II when preoperative MMT was omitted.

**TME or PME in locally advanced upper rectal cancer – really equivalent?**
ID: 898

**Kategorie: DGCH - Onkologische Chirurgie im interdisziplinären Kontext**

Laura Werle[1], Andreas Leha[2], Johanna Kreutzer[1], Friederike Braulke[3], Philipp Schüler[1], Julia Kitz[4], Charlotte de Boer[1], Heiko Sülberg[5], Torsten Liersch[1]

[1]*Klinik für Allgemein-, Viszeral- und Kinderchirurgie - Universitätsmedizin Göttingen, Göttingen, Deutschland*, [2]*Institut für Medizinische Statistik - Universitätsmedizin Göttingen, Göttingen, Deutschland*, [3]*Klinik für Hämatologie und Medizinische Onkologie - Universitätsmedizin Göttingen, Göttingen, Deutschland*, [4]*Institut für Pathologie - Universitätsmedizin Göttingen, Göttingen, Deutschland*, [5]*X-act Cologne Clinical Research GmbH, Köln, Deutschland*

**Background:**
The randomized multicentric GAST-05 phase-IIb study (ISRCTN 35198481) was designed to clarify whether partial mesorectal excision (PME, with a distal cancer-free safety margin of 5 cm) is sufficient compared to total mesorectal excision (TME) for patients with adenocarcinomas (cUICC stages ≥ II) in the upper rectum (> 12 cm from anal verge). In this study we assessed the results of surgery in participants of one GAST-05 study site, taking various clinicopathological parameters into account.

**Materials and methods:**
Surgery was performed in 98 (median age: 69 years) patients (f: 33,7%, m: 66,3%) with clinical stages ≥ II, randomized to PME (experimental) vs. TME (control). Based on standardized staging procedures, surgery was evaluated in terms of feasibility, peri-/postoperatively assessment of specimen`s quality (according to MERCURY criteria), and of acute as well as late adverse events (AE) using the Dindo-classification and NCI-CTCAE-criteria (vs 3.0). Diverse clinico-pathological parameters (e.g. safety margins, circumferential resection margin (CRM), conversion rate between TME and PME, technique of anastomosis) were included in recurrence-free (RFS) and overall (OS) survival analyses using the logrank test, Kaplan-Meier estimator, and multivariable Cox proportional hazard regression models to test interaction effects between selected predictors.

**Results:**
Postsurgical assessment of the specimen revealed UICC stages I to IV in 14.2%, 42.9%, 37.8% and 5.1%, respectively. PME (55.1%) and TME (44.9%) were performed with good, moderate, or poor quality in 82.7%, 16.3% and 1%, respectively. The preferred procedure was open (96,9 %) surgery (laparoscopic in 3,1%) and a protective ileostomy was carried out in 11.1 % (PME) and 79.6 % (TME). Quality of surgery (p = 0,08) and distal safety margins (after PME, p < 0,01) showed impact on the RFS. Within 6 months after surgery most common acute complications (all grades) resulted in abdominal wound healing disorders (WHD) and anastomotic leaks in 16.3% and 9.2%, respectively. The occurrence of fistulas, and urinary disturbances with 6.8% (p = 0.08) and 11.4% (p = 0.24) was more frequent after TME. Anastomotic leaks (AL) and wound healing disorders (WHD) had no significant impact on OS (p = 0.308; HR: 1.5; 95%-CI: 0.70 - 3.10). Late disorders (> 6 months after surgery) with CTCAE-grades > 2 were significantly higher after TME (36.4%) vs. PME (9.3%). The most frequent disorders (grade > 2) after TME were diarrhea, faecal incontinence, erectile and urinary dysfunction in 18.2%, 4.6%, 6.8% and 4.6% vs. only diarrhea in 9.3% after PME, respectively. Restrictive mean of RFS for stage II patients (108.9 months) was significantly better than for stage III (78.7 months; p<0.001). Tumor infiltration depth (< 5 vs. ≥ 5 mm; p = 0.031) and age (p = 0.005) had impact on RFS (univariable models). More detailed results from multivariable survival analyses will be presented at the DCK-2023.

**Conclusion:**
Based on a good surgical quality, PME seems to be not inferior in oncological outcome for patients with advanced upper rectal cancer compared to TME. TME is associated with more acute and late postsurgical complications.

**Total neoadjuvant therapy (TNT) for locally advanced rectal cancer (LARC) – does the surgical plane affect prognosis?**
ID: 902

**Kategorie: DGCH - Onkologische Chirurgie im interdisziplinären Kontext**

Timotheus Ritter[1], Julia Kitz[2], Andreas Leha[3], Lena-Christin Conradi[1], Johanna Kreutzer[1], Heiko Sülberg[4], Philipp Schüler[1], Torsten Liersch[1]

[1]*Klinik für Allgemein-, Viszeral- und Kinderchirurgie - Universitätsmedizin Göttingen, Göttingen, Deutschland*, [2]*Institut für Pathologie - Universitätsmedizin Göttingen, Göttingen, Deutschland*, [3]*Institut für Medizinische Statistik - Universitätsmedizin Göttingen, Göttingen, Deutschland*, [4]*X-act Cologne Clinical Research GmbH, Köln, Deutschland*

**Background:**
Previous clinical trials on patients with LARC have shown that quality of total mesorectal excision (TME) influences local control. For TNT, consisting of sequential preoperative 5-fluorouracil(5-FU)-based chemoradiotherapy (CRT) + oxaliplatin (OX) followed by FOLFOX chemotherapy (CTx) and TME, the prognostic impact of surgical plane is still under debate. In this secondary endpoint analysis of a single arm study, the long-term impact of peri/postoperatively assessed TME was evaluated.

**Materials and methods:**
62 patients (15 f, 47 m; median age: 62 years) with LARC of the lower (43.5%, < 6 cm from anal verge) or mid rectal third (56.5%, ≥ 6 to 12 cm) were included. Staging procedures revealed stages II to IV in 3.2%, 88.7% and 8.1%, respectively. TNT was applied according to the TransValid-B-phase-I/II-protocol (WHO-UTN-U1111-1132-0235) with irradiation (28 x 1.8 Gy; in total 50.4 Gy) and concomitant infusion of 5-FU (250 mg/m$^2$/d, civ, d1 - d14 and d22 - d35) and OX (50 mg/m$^2$ on d1, d8, d22 and d29). Four weeks later, 3 FOLFOX cycles were administered with OX (80-100 mg/m$^2$), FA (400 mg/m$^2$) and 5-FU (2400 mg/m$^2$, civ over 46h) on d1, d15 and d30. TME was performed five weeks later. Perioperatively, the completeness of mesorectal surface was assessed (*ex situ*) by staining the specimen with methylene blue solution (via inferior mesenteric artery). Ink leakages on the surface (surgical plane) were categorized in none (optimal), punctuate (moderate) and extensive leakages (poor quality). Furthermore, TME was prospectively classified as good (mesorectal), moderate (intramesorectal), and poor (muscularis propria) plane by surgeons and independently by pathologists according to MERCURY criteria. All findings were correlated with clinicopathological parameters and survival (e.g. PFS, CSS, OS) using Kaplan Meier estimators, logrank tests and uni-/multivariable models with Cox proportional hazards regression analyses. Interaction models and pairwise contrast tests were built to identify (surrogate-) parameters associated with local and/or distant recurrence.

**Results:**
58 patients (93.5%) underwent TME-surgery (LAR in 60.3%, APR in 39,7%). Postoperatively, stages 0 to IV were diagnosed in 15.5%, 19%, 31%, 24.1%, and 10.3%, respectively. R0, negative CRM status and optimal quality of the specimen were confirmed by the pathologist in 94.8%, 91.4%, and 89.7%, respectively. Perioperatively, intactness of the surgical plane during the methylene blue staining procedure was demonstrated as good, moderate and poor in 32.8%, 56.9% and 3.4%, respectively. Macroscopically, surgeons had categorized TME quality as good, moderate and poor in 82.8%, 15.7% and 1,7%, respectively. In comparison of pathologist- vs. surgeon-based evaluation of TME quality, there was a high agreement (97.9%) in the mesorectal TME cohort, but a significant discrepancy for intramesorectal plane (66,7%). Only in 1 (1.7%) case poor quality of TME-surgery was found by both, surgeons and pathologists. During follow-up (median: 63 months, quartiles: 48 - 103 mo) no local but 16 (25.8%) distant recurrences occurred. PFS and CSS were significantly higher for ypUICC stages ≤ II vs. stages ≥ III (PFS: 111 ± 7.2 mo vs. 67 ± 12.2 mo, p = 0.006; CSS: 130 ± 3.5 mo vs. 94 ± 10.2

mo, $p = 0.004$). OS was 117 ± 6.4 mo for stages ≤ II vs. 91 ± 10.1 mo for stages ≥ III ($p = 0.087$). Patients with optimal TME specimen (no ink leakage and optimal quality grade according MERCURY) showed in trend better PFS ($p = 0.348$) and a lower risk for early (< 36 mo) distant metastases vs. patients without excellent TME. After TNT, patients with stages ≥ III had a higher risk for early distant metastases compared to patients with stages ≤ II ($p < 0.001$). Further detailed results will be presented at the DCK 2023.

**Conclusion:**
TNT approach in LARC provides favorable outcomes for patients with downstaging to stages ≤ II. In addition, excellent quality of TME plane offers a long-term clinical benefit in terms of local and distant recurrences. Valid information on TME quality with prognostic relevance depends on two key players in the multidisciplinary rectal cancer treatment team, the surgeons and the pathologists.

**Targeted Larotrectenib Therapy of Infantile Fibrosarcoma**
ID: 912

**Kategorie: DGCH - Onkologische Chirurgie im interdisziplinären Kontext**

Dietmar Cholewa[1]

[1]*Department of Pediatric Surgery , Inselspital University Hospital, Bern, Schweiz*

**Background:**
Complete surgical resection of infantile fibrosarcoma (IFS) is not possible in every case because of expected mutilating side effects. Tropomyosin receptor kinase inhibitor (Larotrectinib) treatment is a promising alternative or at least neoadjuvant targeted therapy in patient carrying the characteristic gene fusion.

**Materials and methods:**
A 75-day-old male infant presented with a persistend mass in the neck. At birth it was interpreted as birth traumatic damage. Ultrasound and MRI showed a highly perfused soft tissue mass of the neck measuring 4 x 4 x 3 cm. The tumor has grown trough M. trapecius, M. capitis longus et M. splenius capitis without respecting interfaces. A biopsy was performed revealing characteristic features of fibrosarcoma. Molecular Cancer Assay RNA Panel confirmed presence of an ETV6-NTRK3 (neurotrophic receptor tyrosine kinase 3) fusion. Complete surgical resection was viewed as possible mutilating. The patient was enrolled in phase 1/2 study NCT02637687 and larotrectinib oral treatment 60 mg/d started.

**Results:**
By 2 weeks of treatment there was a considerable decrease of the tumor mass. Repeat MRI 2 month after showed partial response with 89 % volume reduction. Again complete surgical resection was viewed as possible mutilating. Repeat MRI 4 month after did not dedect the tumor any more. Repeat true cut histology at the same time was without living tumor cells. Treatment was finished. Repeat MRI 4, 7, 12 month after treatment stop showed complete response. Therefore we continued with observation.

**Conclusion:**
In selected cases first line tropomyosin receptor kinase inhibitor Larotrectinib treatment it seems recommendable. Is crucial that the pediatric surgeon consider this in the assessment of complete resectability.

**Response to total neoadjuvant therapy (TNT) in rectal cancer– a predictor for anything?**
ID: 925

**Kategorie: DGCH - Onkologische Chirurgie im interdisziplinären Kontext**

Timotheus Ritter[1], Andreas Leha[2], Johanna Kreutzer[1], Julia Kitz[3], Leif-Hendrik Dröge[4], Beate Michels[1,5], Ute König[6], Heiko Sülberg[7], Philipp Schüler[1], Torsten Liersch[1]

[1]*Klinik für Allgemein-, Viszeral- und Kinderchirurgie - Universitätsmedizin Göttingen, Göttingen, Deutschland*, [2]*Institut für Medizinische Statistik - Universitätsmedizin Göttingen, Göttingen, Deutschland*, [3]*Institut für Pathologie - Universitätsmedizin Göttingen, Göttingen, Deutschland*, [4]*Klinik für Strahlentherapie und Radioonkologie - Universitätsmedizin Göttingen, Göttingen, Deutschland*, [5]*Klinik für Hämatologie und Medizinische Onkologie - Universitätsmedizin Göttingen, Göttingen, Deutschland*, [6]*Klinik für Gastroenterologie, gastrointestinale Onkologie & Endokrinologie - Universitätsmedizin Göttingen, Göttingen, Deutschland*, [7]*X-act Cologne Clinical Research GmbH, Köln, Deutschland*

**Background:**
The occurrence of early distant metastases (DM) is the most limiting factor in multimodal treatment (MMT) of locally advanced rectal cancer (LARC). TNT, consisting of preoperative 5-fluorouracil(5-FU)-based chemoradiation (CRT) + oxaliplatin (OX), followed by 3 cycles of FOLFOX chemotherapy (CTx) and total mesorectal excision (TME), is an innovative MMT for patients with LARC (< 12 cm from anal verge). TNT is expected to achieve high rates on complete (CR) or near complete (nCR) remissions, followed by low rates of local and distant recurrences as well as favorable outcome.

**Materials and methods:**
62 patients (15 f, 47 m; median age: 62 years) with LARC of the lower (43.5%; < 6 cm from anal verge) or mid rectal third (56.5%, ≥ 6 to 12 cm) entered this monocentric single arm study (as performed according to the TransValid-B-phase-I/II-protocol, WHO-UTN-U1111-1132-0235). Staging revealed clinical stages II to IV in 3.2%, 88.7% and 8.1%, respectively. Positive circumferential resection margins (CRM < 2 mm) and nodal status (N+) were diagnosed in 85.5% and 96.8%. CRT was performed with 28 fractions of irradiation (1.8 Gy each, total dose 50.4 Gy) and concomitant CTx with 5-FU (250 $mg/m^2/d$, civ, d1 - d14 and d22 - d35) and OX (50 $mg/m^2$ on d1, d8, d22 and d29). Four weeks later, 3 FOLFOX cycles were applied with OX (80 to 100 $mg/m^2$), followed by folinic acid (400 $mg/m^2$) and 5-FU (2400 $mg/m^2$ civ over 46h) on d1, d15 and d30. TME was performed five weeks later with assessment of the TME specimen (MERCURY-criteria, $7^{th}$ TNM/UICC-classification) and TNT-induced tumor regression (using a five-point TRG system according to Dworak et al. 1997). Survival (e. g. PFS, OS and CSS) was calculated using Kaplan-Meier estimators, logrank tests and multiparametric Cox proportional-hazards regression models.

**Results:**
58 patients (93.5%) underwent TME-surgery with R0, negative CRM and N status as well as optimal TME quality in 94.8%, 91.4%, 69.0%, and 89.7%, respectively. Postoperatively, stages 0 to IV and CR (ypT0 N0) were diagnosed in 15.5%, 19%, 31%, 24.1%, 10.3% and 17.2%, respectively. nCR (ypT1-T2 N0 and TRG 2 or TRG 3) was achieved in 17.2%. During 63 months (median) follow-up (quartiles: 48-103 mo) no local relapse occurred. The cumulative incidence of DM at 3 and 5 years was 22.6% and 24.2%, respectively. Patients with ypUICC stages ≥ III had a higher and earlier risk for DM vs. stages ≤ II ($p < 0.001$). The same clinical effect was found in patients with CR/nCR vs. poor response ($p = 0,043$). After TNT, PFS and CSS were enhanced for stages ≤ II vs. stages ≥ III (PFS: 111 ± 7.2 mo vs. 67 ± 12.2 mo, $p = 0.006$; CSS: 130 ± 3.5 mo vs. 94 ± 10.2 mo, $p = 0.004$). OS resulted in 117 ± 6.4 mo for stages ≤ II vs. 91 ± 10.1 mo for stages ≥ III ($p = 0.087$). OS and PFS for patients with good (CR/nCR) vs. poor response were 125 ± 7.0 mo vs. 101.5 ± 7.8 mo ($p = 0.043$) and 114 ± 9.6 mo vs 87.9 ± 9.2 mo ($p = 0.083$). Further detailed results will be presented at the DCK 2023.

**Conclusion:**
TNT-induced CR/nCR-status or residual tumor stages ≤ II are highly beneficial for PFS, CSS and OS and associated with a lower risk for distant metastases. Patients with CR/nCR status after TNT would be suitable candidates for a "watch and wait" strategy. In patients with poor response (e. g. stages ≥ III) to TNT alone, the value of further adjuvant CTx should be discussed and tested in multicenter trials.

**Upfront surgery in upper rectal cancer followed by adjuvant FOLFOX CTx – what can we await?**
ID: 933

**Kategorie: DGCH - Onkologische Chirurgie im interdisziplinären Kontext**

Charlotte de Boer[1], Andreas Leha[2], Julie Schanz[3], Beate Michels[3], Johanna Kreutzer[1], Heiko Sülberg[4], Philine-Sophie Hoyer[1], Timotheus Ritter[1], Torsten Liersch[1]

[1]*Klinik für Allgemein-, Viszeral- und Kinderchirurgie - Universitätsmedizin Göttingen, Göttingen, Deutschland*, [2]*Institut für Medizinische Statistik - Universitätsmedizin Göttingen, Göttingen, Deutschland*, [3]*Klinik für Hämatologie und Medizinische Onkologie - Universitätsmedizin Göttingen, Göttingen, Deutschland*, [4]*X-act Cologne Clinical Research GmbH, Köln, Deutschland*

**Background:**
Up to date, there is an ongoing debate about optimal surgery (total vs. partial mesorectal excision, TME vs. PME) and the requirement of adjuvant treatment (± 5-FU monotherapy vs. FOLFOX chemotherapy, CTx) for patients with locally advanced adenocarcinomas (LARC, stages II and III) in the upper rectum (≥ 12 cm from anal verge). In this single center study, we evaluate safety, feasibility and efficacy of 4 cycles FOLFOX CTx in patients with upper rectal cancer (stages II and III) after standardized, peri-/ postoperatively controlled TME vs. PME (according to the GAST-05 phase-IIb trial, ISRCTN35198481).

**Materials and methods:**
Upfront surgery was performed in 98 trial participants (median age: 69 years, range: 38 – 87 years; f: 33, m: 65). After confirmation of UICC stages II or III, adCTx with 4 cycles of FOLFOX (FA: 400 $mg/m^2$, 5-FU: 2400 $mg/m^2$, OX: 100 $mg/m^2$) was indicated. For all patients, the occurrence of acute / late adverse events (NCI-CTC-AE grades 1 to 4, vs 3.0) due to treatment was recorded. Survival was calculated using the logrank test, Kaplan-Meier estimator, and uni-/ multiparametric Cox proportional hazard regression models.

**Results:**
Postsurgical quality of the specimen was good, moderate, or poor (according to MERCURY-criteria) in 84.7%, 14.3% and 1.0%, respectively. AdCTx could be started in 79.5% of patients (stages II / III in 30 and 32 patients) and was administered in 96.6% (regardless of any dose reduction). The applied dosages of 5-FU / OX were 90.8% / 73.4% of the target. In total, 772 AEs (all grades) with hematologic (26.6%), gastrointestinal (28.6%), and neurologic (19.6%) side-effects occurred, with clinically relevant CTC-AE grades 3 / 4 in only 3.5%. During follow-up (in median > 5 years), 386 late AEs were recorded, including CTC-AE grades 3 / 4 in 11.1%. Chronic neurotoxicity was present in 24.4% (grade 2 in 17.0%) and diarrhea in 34.7% (grades 3 / 4 in 18.4%). A low anterior resection syndrome (LARS) was diagnosed in 44.1% of evaluable patients; this was more frequent after TME ($p = 0.007$). There was no correlation between the presence of LARS and adCTx ($p = 0.209$). Stage III patients showed a 5-year DFS of 65.6% (vs. 40.0% without adCTx; $p = 0.058$); 5-year OS was 78.1% (vs. 40.0% without adCTx, $p = 0.003$). 8-months landmark analyses confirmed the benefit in OS for LARC patients treated with adCTx ($p = 0.0043$).

**Conclusion:**
After standardized surgery, adCTx with FOLFOX offers longer survival to patients with stage III upper rectal cancer. AdCTx was applicable with a good safety profile, a high compliance rate, and favorable treatment adherence.

**MRI-based re-assessment after preoperative multimodal treatment (MMT) in rectal cancer – really an early prognosticator?**
ID: 935

**Kategorie: DGCH - Onkologische Chirurgie im interdisziplinären Kontext**

Torsten Liersch[1], Ali Seif Amir Hosseini[2], Timotheus Ritter[1], Carsten-Oliver Sahlmann[3], Johanna Kreutzer[1], Leif-Hendrik Dröge[4], Julia Kitz[5], Andreas Leha[6]

[1]*Klinik für Allgemein-, Viszeral- und Kinderchirurgie - Universitätsmedizin Göttingen, Göttingen, Deutschland*, [2]*Institut für Diagnostische und Interventionelle Radiologie - Universitätsmedizin Göttingen, Göttingen, Deutschland*, [3]*Klinik für Nuklearmedizin - Universitätsmedizin Göttingen, Göttingen, Deutschland*, [4]*Klinik für Strahlentherapie und Radioonkologie - Universitätsmedizin Göttingen, Göttingen, Deutschland*, [5]*Institut für Pathologie - Universitätsmedizin Göttingen, Göttingen, Deutschland*, [6]*Institut für Medizinische Statistik - Universitätsmedizin Göttingen, Göttingen, Deutschland*

**Background:**
Baseline 1.5 Tesla MRI (MRI-I) is standard for locally advanced rectal cancer (LARC) to indicate preoperative 5-FU-based chemoradiotherapy (preopCRT) followed by total mesorectal excision (TME). The prognostic significance of pre-surgical re-assessment with MRI (MRI-II in difference (Δ) to MRI-I) is still controversial.

**Materials and methods:**
In this monocentric study, 125 patients (47 f, 78 m; median age: 68 years) with LARC (clinical stages II to IV in 5.6%, 92.0%, and 2.4%, respectively) received preopCRT ± Oxaliplatin followed by TME surgery. Imaging and post-surgical parameters were tested for association with recurrence-free (RFS), overall (OS) and cancer-specific (CSS) survival. Three multiple Cox regression models were built using grouped lasso penalization: (1) pre-surgical (imaging), (2) post-surgical (pathological) and (3) post-treatment (all variables). Patients were classified in high- or low-risk survivors in a 10-fold cross validation. The post-surgical models were extended with additionally informative imaging parameters as confirmed by likelihood ratio tests.

**Results:**
Post-surgical staging revealed ypUICC-stages 0 to IV in 13.6%, 28.0%, 25.6%, 23.2% and 9.6%, respectively. Restricted mean for RFS, OS and CSS were 88.9 ± 5.5 months, 100.4 ± 4.9 months and 119.4 ± 4.7 months. Survival rates after 2.5, 5 and 7.5 years amounted to 68.8%, 59.9%, 56.4% for RFS; to 84.8%, 68.7%, 60.3% for OS, and to 89%, 80% and 73% for CSS, respectively. In the pre-surgical models increased hazard ratios (HR) for RFS were observed in MRI-I_Δ_MRI-II for T status ($p = 0.01$) and MRI-I_Δ_MRI-II for TE (longitudinal tumor extent) ($p = 0.04$). Increased HR for OS was seen in MRI-I_Δ_MRI-II for TE ($p = 0.02$). Cross validation of the pre-surgical risk models differentiated survivors in RFS ($p=0.019$) and OS ($p<0.001$).

**Conclusion:**
MRI monitoring is useful for early prediction of clinical survival benefit in advanced rectal cancer. Our findings strongly suggest MRI as an easily available pre-surgical tool for early adjustment of (multimodal) treatment.

**Magnetic resonance imaging (MRI) in upper rectal cancer – the surgical point of view**
ID: 938

**Kategorie: DGCH - Onkologische Chirurgie im interdisziplinären Kontext**

Anastasia Dr. Scheuring[1], Andreas Leha[2], Ali Seif Amir Hosseini[3], Johanna Kreutzer[1], Laura Werle[1], Timotheus Ritter[1], Carsten-Oliver Sahlmann[4], Torsten Liersch[1]

[1]*Klinik für Allgemein-, Viszeral- und Kinderchirurgie - Universitätsmedizin Göttingen, Göttingen, Deutschland*, [2]*Institut für Medizinische Statistik - Universitätsmedizin Göttingen, Göttingen, Deutschland*, [3]*Institut für Diagnostische und Interventionelle Radiologie - Universitätsmedizin Göttingen, Göttingen, Deutschland*, [4]*Klinik für Nuklearmedizin - Universitätsmedizin Göttingen, Göttingen, Deutschland*

**Background:**
Pelvic MRI is well established in staging of advanced cancer (stages ≥ II) in the mid and lower third of the rectum (< 12 cm from anal verge). In upper rectal cancer (≥ 12 cm), its value is still under discussion.

**Materials and methods:**
In this single-center project, staging with MRI, rES, and CT was compared with outcomes after upfront surgery (TME vs PME) in 98 participants of the GAST-05 phase II trial (ISRCTN35198481). MRI accuracy (mrAC) was determined based on postsurgical cancer stage (7th TNM/UICC-classification). Parameters such as T, N, and UICC status, circumferential resection margin (CRM), extramural vascular infiltration (EMVI) were analyzed for recurrence-free survival (RFS) using logrank test, Kaplan-Meier estimators, and multiparametric Cox proportional hazards-regression models. Blinded MRI re-evaluations (MRI 2nd read) of tumor site, peritoneal reflection fold (PRF), and mrEMVI were then performed in 35 patients.

**Results:**
Postsurgical staging revealed stages I to IV in 14.3, 43.9, 36.7 and 5.1%, respectively. mrAC was 79.3% for distinguishing stages ≥ II vs. < II and 74.5% and 85.1% for N status and stage (≥ II), respectively (vs. 59.6% and 72.3% with rES). In addition, MRI was superior to rES and CT in describing CRM and EMVI. Using survival models in cohort A (n = 47; with MRI) vs. B (n = 51; without MRI), patients age, stage ≥ III, and T status were shown to have significant impact on RFS. MRI 2nd read revealed that the PRF was significantly closer to the AV and the aboral tumor margin was more distant in females (median 40 mm vs 12.5 mm). mrAC in EMVI was 48.6%, but the initially postulated positive mrEMVI (82.9%) was confirmed as pL-/V-/Pn + status in only 11 specimens (31.4%). Patients with a negative pL-/V-/Pn status had better CSS.

**Conclusion:**
Staging MRI combined with rES and rigid rectoscopy should be used in patients with cancer in the upper rectum (≥ 12 cm) and rectosigmoid (> 16 cm to 30 cm) to optimize preoperative assessment of T, N, UICC, CRM, EMVI status and tumor location. Considering surgical and anatomical landmarks, MRI staging in upper rectal cancer could facilitate the decision for a more individualized treatment.

**Peri-/postoperative assessment of rectal cancer specimens after preoperative chemoradiotherapy (CRT) vs. upfront surgery – what can we learn?**
ID: 940

**Kategorie: DGCH - Onkologische Chirurgie im interdisziplinären Kontext**

Tatiana Grammatikopoulou[1], Andreas Leha[2], Julie Schanz[3], Julia Kitz[4], Johanna Kreutzer[1], Heiko Sülberg[5], Recca Talaulicar[1], Timotheus Ritter[1], Torsten Liersch[1]

[1]*Klinik für Allgemein-, Viszeral- und Kinderchirurgie - Universitätsmedizin Göttingen, Göttingen, Deutschland*, [2]*Institut für Medizinische Statistik - Universitätsmedizin Göttingen, Göttingen, Deutschland*, [3]*Klinik für Hämatologie und Medizinische Onkologie - Universitätsmedizin Göttingen, Göttingen, Deutschland*, [4]*Institut für Pathologie - Universitätsmedizin Göttingen, Göttingen, Deutschland*, [5]*X-act Cologne Clinical Research GmbH, Köln, Deutschland*

**Background:**
It is assumed, that both high-quality surgery in rectal cancer and comprehensive (histo-) pathologic work-up of the specimen have major impact on local recurrence rates in patients with locally advanced rectal cancer (LARC). The prognostic impact of clinicopathological parameters, like tumor location (< 12 cm vs ≥ 12 cm from anal verge), circumferential resection margin (CRM), lymphnode metastases (LNM) and UICC stage under terms of preoperative CRT or upfront surgery is controversial. We evaluate standardized peri-/postoperative assessment of rectal specimen after total (TME) or partial (PME) mesorectal excision in patients treated by upfront surgery or preoperative 5-FU-based CRT ± Oxaliplatin (OX).

**Materials and methods:**
402 participants of the CAO/ARO/AIO-94, -04 and GAST-05 (ISRCTN 35198481) trials with stage ≥ II rectal cancers were included in this monocentric study. Peri-/postoperative controls of the specimen were performed according to trial protocols (e.g. MERCURY criteria). The impact of selected parameters on survival was investigated by using logrank test, Kaplan-Meier estimators, uni-/multiparametric Cox proportional hazard regression and interaction effects testing models.

**Results:**
Optimal quality in TME-/PME-surgery was achieved in 80.4 % and 78.7 %, respectively. After preopCRT + OX, it was 82.5 % and 77.8 % vs. 76.6 % and 70.3 % (- OX). A negative CRM status (> 1 mm) was determined in 87.8 %. After upfront surgery, a CRM of < 1 mm or 1 mm was present in 14 % and 9.5 % vs. 3.1 % and 2.6 % (after preopCRT). PreopCRT resulted in a reduction of LNM count; the ratio of LNM to LN had decreased to 0.027.
In the multivariable Cox model, LARC patients (≥ 12 cm) showed a higher risk of CSS events depending on CRM + and upfront surgery (HR: 0.2; adjusted p = 0.005). Paired contrast tests pointed out, that patients with CRM + after upfront surgery might have a relevant risk for death from rectal cancer (HR: 3.8; p = 0.031). Patients with LARC (< 12 cm) and ypN0-status showed prolonged OS (HR: 0.39; p < 0.001), like in LARC (≥ 12 cm) after preopCRT (HR: 1.7; p = 0.007). DFS was better in patients with ypN0 (HR: 0.41; p < 0.001), too. For carcinomas in the upper rectum, patients with N0 status after upfront surgery (HR: 0.44; p = 0.041) or after preopCRT (HR: 0.25; p = 0.035) had a benefit in DFS.
Accordingly, contrast tests demonstrated a high risk for death from LARC in N+ patients (LARC < 12 cm) after preopCRT (HR: 0.24; p < 0.001) and for LARC in the upper rectum (HR: 0.011; p = 0.001). Patients with upper LARC, pN0 status, and upfront surgery had a remarkable risk of death from another cause (HR: 0.031; adj p = 0.008). More results will be presented in detail at DCK 2023.

**Conclusion:**
Assuming high-quality surgery and comprehensive (histo-)pathologic work-up of the TME/PME-specimen, visceral surgeons and pathologists have key roles in MMT of patients with LARC.

**Should I trust the guidelines? - the case of neoadjuvant therapy in rectal cancer**
ID: 954

**Kategorie: DGCH - Onkologische Chirurgie im interdisziplinären Kontext**

Pawel Mroczkowski[1], Selim Atay[1], Iurii Snopok[1]

[1]*Klinik für Chirurgie, Bochum, Deutschland*

**Background:**
Clinical guidelines claim to be a crucial point within Evidence Based Medicine (EBM). However the production process of guidelines remains not always transparent, with many stakeholders presenting controversial interests. We have analysed the validity of recommendation for neoadjuvant therapy in rectal cancer presented in different international guidelines.

**Materials and methods:**
Systematic search of internet databases revealed current (published within last 10 years) guidelines for treatment of rectal cancer. The analysis included the authorship of the guidelines, conflicts of interests, expressed recommendation, validity of references.

**Results:**
32 guidelines from Europe, Asia, South and North America, Australia were identified. Despite the fact that rectal cancer is a primary surgical disease, surgeons / surgical societies consist usually only a marginal part of authors. Most guidelines recommend neoadjuvant treatment using historical references and ignoring primary surgical options. The side effects and functional impairments of combined therapy regimes are rarely mentioned. A significant part of authors reveals conflicts of interest and connections with pharmaceutical industry.

**Conclusion:**
Primary surgical options are not adequately presented in current international treatment guidelines for rectal cancer. The recommendation for neoadjuvant therapy is based on sparse historical data. Unfortunately the quality of presented evidence is low, making implementation of the recommendations questionable.

**Postoperative sleep disturbances: A retrospective cohort analysis of gynecological, urologic and general surgical patients**
ID: 86

**Kategorie: DGCH - Perioperative Medizin in der Chirurgie**

Anne-Sophie Winter[1], Christian Haverkamp[2], Roman Huber[1], Ann-Kathrin Lederer[3]

[1]*Uniklinik Freiburg, Uni-Zentrum Naturheilkunde, Innere Medizin II, Freiburg im Breisgau, Deutschland*, [2]*Uniklinik Freiburg, Institut für Digitalisierung in der Medizin, Freiburg im Breisgau, Deutschland*, [3]*Universitätsmedizin der Johannes Gutenberg-Universität Mainz, Klinik für Allgemein-, Viszeral- und Transplantationschirurgie, Mainz, Deutschland*

**Background:**
Postoperative sleep disturbances appear to be a common issue after surgery being treated with sleep-promoting medication. It is suggested that sleep disturbances might detoriate the postoperative recovery. To date, robust data about frequency of occurence and evidence of medicinal approaches for treatment are lacking.

**Materials and methods:**
We performed a retrospective cohort analysis of 21168 urological, gynecological and general surgical patients of the University Medical Center Freiburg, Germany, who underwent surgery between 2015 and 2020. Target parameters were the usage of sleep-promoting medication to estimate the occurrence of postoperative sleep disturbances as well as the kind of sleep medication with a special focus on herbal medicine.

**Results:**
Drug-treated sleep disturbances occurred in 15% (n = 3083) of the patients. Classic benzodiazepines (n = 1138, 36.9%) were the most applied drugs followed by valerian (n = 814, 26.4%). The majority of patients received monotherapies. Age, length of stay and comorbidities were associated with demand for sleep medication in general ($p < 0.001$). Valerian monotherapy was more common in women (OR 1.53, 95% CI 1.33-1.77, $p < 0.001$), elderly patients (OR 1.50, 95% CI 1.29-1.75, $p < 0.001$) and patients with prolonged hospital stay (OR 2.23, 95% CI 1.91-2.61, $p < 0.001$).

| | **Sleep Medication (n = 3083)** | **No Sleep Medication (n = 18085)** | **Total (n = 21168)** |
|---|---|---|---|
| **Sex** | | | |
| Male | 1473 (47.8%) | 8718 (48.2%) | 10191 (48.1%) |
| Female | 1610 (52.2%) | 9367 (51.8%) | 10977 (51.9%) |
| **Age** | | | |
| Mean (SD) | 62.40 (16.44) | 56.06 (17.78) | 56.99 (17.73) |
| Range | 18.00 - 98.00 | 18.00 - 106.00 | 18.00 - 106.00 |
| **Age categories** | | | |
| <60 years | 1160 (37.6%) | 9706 (53.7%) | 10866 (51.3%) |
| 60+ years | 1923 (62.4%) | 8379 (46.3%) | 10302 (48.7%) |
| **Department of hospitalization** | | | |
| General and Visceral Surgery | 1482 (48.1%) | 8034 (44.4%) | 9516 (45.0%) |
| Urology | 881 (28.6%) | 5430 (30.0%) | 6311 (29.8%) |
| Gynecology | 720 (23.4%) | 4621 (25.6%) | 5341 (25.2%) |

| | Sleep Medication (n = 3083) | No Sleep Medication (n = 18085) | Total (n = 21168) |
|---|---|---|---|
| **Hospital stay** | | | |
| Mean (SD, days) | 9.33 (7.49) | 6.03 (5.87) | 6.51 (6.24) |
| Range | 1.42 - 77.92 | 0.21 - 99.25 | 0.21 - 99.25 |
| <=5 days | 920 (29.8%) | 10713 (59.2%) | 11633 (55.0%) |
| >5 days | 2163 (70.2%) | 7372 (40.8%) | 9535 (45.0%) |
| **Localization of surgery** | | | |
| Hernia repair | 85 (2.8%) | 654 (3.6%) | 739 (3.5%) |
| Digestive tract | 837 (27.1%) | 4848 (26.8%) | 5685 (26.9%) |
| Urinary tract | 513 (16.6%) | 3015 (16.7%) | 3528 (16.7%) |
| Male reproductive organs | 377 (12.2%) | 2166 (12.0%) | 2543 (12.0%) |
| Female reproductive organs | 222 (7.2%) | 2282 (12.6%) | 2504 (11.8%) |
| Obstetrics | 6 (0.2%) | 120 (0.7%) | 126 (0.6%) |
| Breast surgery | 331 (10.7%) | 1409 (7.8%) | 1740 (8.2%) |
| Other | 712 (23.1%) | 3591 (19.9%) | 4303 (20.3%) |
| **Elixhauser, van Walraven Score** | | | |
| Mean (SD, points) | 6.05 (7.53) | 3.89 (6.37) | 4.21 (6.59) |
| Range | -14.00- 53.00 | -11.00 – 58.00 | -14.00 – 58.00 |

| | | | |
|---|---|---|---|
| **Sleep Medication** | | | |
| Mono: Valerian | 814 (26.4%) | 0 (0.0%) | 814 (3.8%) |
| Mono: Classic benzodiazepines | 1138 (36.9%) | 0 (0.0%) | 1138 (5.4%) |
| Mono: Benzodiazepine receptor agonists | 538 (17.5%) | 0 (0.0%) | 538 (2.5%) |
| Comb: Valerian/benzodiazepine | 191 (6.2%) | 0 (0.0%) | 191 (0.9%) |
| Comb: Valerian/benzodiazepine/ other | 5 (0.2%) | 0 (0.0%) | 5 (0.0%) |
| Comb: Valerian/other | 22 (0.7%) | 0 (0.0%) | 22 (0.1%) |
| Mono: Risperidone or haloperidol | 62 (2.0%) | 0 (0.0%) | 62 (0.3%) |
| Mono: Melperone | 84 (2.7%) | 0 (0.0%) | 84 (0.4%) |
| Mono: Quetiapine | 54 (1.8%) | 0 (0.0%) | 54 (0.3%) |
| Other | 175 (5.7%) | 0 (0.0%) | 175 (0.8%) |
| Rescue therapy, valerian to benzodiazepines within twelve hours | 121 (55.5%) | 0 | 121 (55.5%) |
| No rescue | 97 (44.5%) | 0 | 97 (44.5%) |

**Conclusion:**
Sleep disturbances are a typical postoperative issue in surgical patients, but evidence for treatment is lacking. In our study, valerian played an important role in the treatment of postoperative sleep disturbances, and it appears to be a promising therapeutic approach especially in women, older and sicker patients and those with prolonged hospital stay. Further research has to clarify the efficacy of sleep-promoting drugs in surgical patients.

**The validity of an App-based risk scoring defining an aerobic endurance prehabilitation program (Patronus Prehab App) in patients undergoing elective major surgery: The prospective, interventional PROTEGO MAXIMA Trial**
ID: 168

**Kategorie: DGCH - Perioperative Medizin in der Chirurgie**

Svenja Sliwinski[1], Fatima Faqar-Uz-Zaman[1], Natalie Filmann[2], Lisa Mohr[3], Julia Dreilich[3], Dora Zmuc[4], Charlotte Detemble[1], Wolf Otto Bechstein[1], Johannes Fleckenstein[3], Andreas Schnitzbauer[1]

[1]*University Hospital Frankfurt, Goethe University Frankfurt/Main,, Department for General, Visceral, Transplant and Thoracic Surgery, Frankfurt/Main, Germany, Frankfurt am Main, Deutschland*, [2]*Goethe University Frankfurt/Main, Institute of Biostatistics and Mathematical Modeling, Frankfurt/Main, Germany, Frankfurt am Main, Deutschland*, [3]*University Hospital Frankfurt, Goethe University Frankfurt/Main, Institute for Sports Medicine, Frankfurt/Main, Germany, Frankfurt am Main, Deutschland*, [4]*Ljubljana Central Medical School, Ljubljana, Slovenia*

**Background:**
Prehabilitation aims at improving patients' abilities prior to interventions to positively impact perioperative outcomes. The multimodal regimes should consist of exercise, nutritional supplementation, and psychosocial interventions. To understand the improvement of a prehabilitation intervention, a set of well-measurable risk data is required. This study aims for risk score cut-offs correlated to postoperative outcomes. The hypothesis was that the (Calculated Risk analysis index) RAI-C score, the Easter Cooperative Oncology Group (ECOG) score, and the Timed-Up and Go (TUG)-test is predictive of postoperative outcomes by day 90 after surgery.

**Methods:**
Patients undergoing major surgery (visceral, urology, vascular, thoracic, gynecologic) were risk-scored with a digital software tool (Patronus Prehab App), and the scores correlated with 90-day outcomes (ICD 10 diagnosis, surgical OPS codes, Clavien-Dindo (CD) classification and survival). ROC analyses were performed to obtain cut-off values for the Rai-C score and the Timed Up and Go Test (TUG). $\chi^2$ tests were performed for ECOG scoring and univariate analysis.

**Results:**
A total of 104 patients were included (32.7% female and 67.3% male). The mean age was 60.79 ± 13.834 years. The mean RAI-C score was 21.68 ± 10.592 and the mean TUG was 7.8 ± 2.671. The ECOG score was 0 in 89.4% and 1 in 10.6% of patients. Cancer was the leading diagnosis in 71.2% of patients. 90-day mortality was 4.8%. Complication rates at 90-days were 52%. Major complication rates CD>II was 24%. The mortality predictive cut-off value for the Rai-C score was 24 (sensitivity 80%; specificity 65%; AUC: 0.791; p=0.001), for the TUG 8 seconds (sensitivity 70%; specificity 70%; 0.797; p=0.002). Predictive markers for postoperative mortality were the activity of daily living items mobility (p=0.000), toilet use (p=0.000) and ECOG (p=0.028).

**Conclusion:**
The risk assessment within the software is a viable method to identify risk factors before major surgeries. Risk scores (RAI-C, ECOG, TUG) correlate with postoperative mortality. Combining 3 simple scores increases the reliability of detecting valid preoperative risk factors without relevant extra workload for doctors and other medical staff in the preoperative setting.

**The safety and validity of an App-based prehabilitation program (Patronus Prehab App) in patients undergoing elective major surgery: The prospective, interventional PROTEGO MAXIMA Trial**
ID: 189

**Kategorie: DGCH - Perioperative Medizin in der Chirurgie**

Fatima Faqar-Uz-Zaman[1], Natalie Filmann[2], Svenja Sliwinski[1], Lisa Mohr[3], Julia Dreilich[3], Dora Zmuc[4], Charlotte Detemble[1], Wolf Bechstein[1], Johannes Fleckenstein[3], Andreas Schnitzbauer[1]

[1]*Department for General, Visceral, Transplant and Thoracic Surgery, University Hospital Frankfurt, Frankfurt am Main, Deutschland*, [2]*Institute of Biostatistics and Mathematical Modeling, Goethe University Frankfurt/Main, Frankfurt am Main, Deutschland*, [3]*Department of Sports Medicine and Exercise Physiology, Goethe University Frankfurt, Frankfurt am Main, Deutschland*, [4]*Ljubljana Central Medical School, Ljubljana, Slovenia*

**Background:**
Major surgery is associated with a high risk for postoperative complications, having a deleterious impact on mortality and morbidity, particularly in frail patients with a poor cardiopulmonary reserve[1,2]. Prehabilitation, including aerobic exercise training, aims to improve patients' physical fitness before major surgery and reduce postoperative complications[3-6]. The Patronus Prehab App provides a novel approach for individual, risk-based endurance exercise software for patients before major surgery. The purpose of this study is to assess the validity and safety of the software in accordance with the Medical Drug Regulation (MDR) using wrist-worn wearables devices to measure heart rate (HR) and distance.

**Materials and methods:**
Healthy students and patients (± cardiopulmonary disease) undergoing major elective surgery were enrolled. Participants performed a 6-min walking test (6MWT) and a 37-min interval training on a treadmill based on heart rate reserve, wearing standard electrocardiographic (ECG) limb leads and two smartwatches, which were driven by the test software. Four device combinations were assessed: iPhone 13 + Apple Watch 7, iPhone SE + Apple Watch 3, Samsung A52 or Google Pixel 6 + Samsung Watch 4. An ergometry-based analysis was performed by monitoring HR and distance measurement of the wearables and comparing them to standard ECG and distance data of the treadmill. The hypothesis was that smartwatch measurements will not exceed the mean difference of ± ≤ 10 bpm and an acceptable range of variation of ± ≤ 50bpM. Further, usability and lab testing were performed by analyzing blood samples before and after the exercise training and assessing the alarm settings of the devices.

**Results:**
We included 49 participants from different Surgical Departments (22.4% healthy students, 21.1% Visceral, 64.8% Urology, 7.9% Vascular, and 5.3% Thoracic Surgery). Bland-Altman analysis revealed a mean HR difference within ± ≤ 10 bpm compared to standard ECG with the lowest variation during the 6MWT for Apple 13 (3.7 [95% CI 0.3;7.2] vs Apple SE (4.0 [95% CI -0.2;8.2]), 4.8 Samsung (4.8 [95% CI 1.4;8.2]), Pixel 6 (4.5 [95% CI 0.5;8.5])), and during the interval training for Pixel 6 (-0.6 [95% CI -5.7;4.4]) vs Apple 13 (6.1 [95% CI 3.0;9.2]), Apple SE (1.4 [95% CI -0.9;3.7]) and Samsung (2.5 [95% CI -0.3;5.2])). More than 90% of all HR measurements fell into the predefined range of variation. Mean difference of distance compared to treadmill was lowest for Appel SE during both exercises with 25m and -4m, respectively (6MWT: 25 [95% CI -47;97]), vs Apple 13 (-45 [95% CI -109;20]; interval training: -4 [95% CI -502;495]), vs Apple 13 (392 [95% CI -121;906]). Laboratory values showed significant differences for sodium, chloride, creatine kinase and lactate dehydrogenase, but no significant increase for creatine-kinase-MB ($p$ = 0.06) and lactate ($p$ = 0.7). Usability data for alarm awareness displayed on the devices either by color or haptic vibration was realized within a mean of

12.2s (2-45s). To date, no serious adverse event occurred with regard to an emergency during exercising occurred

**Conclusion:**
All tested devices provided an acceptable HR accuracy. Regarding safety, there were no critical lab detections for clinically relevant parameters, and usability testing revealed prompt and adequate reactions. The findings provide preliminary support that the app-based prehabilitation program can be safely applied to patients before major surgery. Subsequent studies, including a randomized controlled trial, will be required to assess the effectiveness of the tested software and its impact on patient outcomes. Trial registration number CIV-21-07-037311.

**Literature:**
[1]World Health Organization. WHO guidelines for safe surgery 2009: Safe surgery saves lives. Geneva, Switzerland: World Health Organization Patient Safety; 2009.
[2]Hall DE, Arya S, Schmid KK, Blaser C, Carlson MA, Bailey TL, et al. Development and Initial Validation of the Risk Analysis Index for Measuring Frailty in Surgical Populations. JAMA Surg. 2017;152
[3]Snowden CP, Prentis J, Jacques B, Anderson H, Manas D, Jones D, Trenell M. Cardiorespiratory fitness predicts mortality and hospital length of stay after major elective surgery in older people. Ann Surg. 2013;257.
[4]Barberan-Garcia A, Ubré M, Roca J, Lacy AM, Burgos F, Risco R, et al. Personalised Prehabilitation in High-risk Patients Undergoing Elective Major Abdominal Surgery: A Randomized Blinded Controlled Trial. Ann Surg. 2018;267
[5]Moran J, Guinan E, McCormick P, Larkin J, Mockler D, Hussey J, et al. The ability of prehabilitation to influence postoperative outcome after intra-abdominal operation: A systematic review and meta-analysis. Surgery. 2016;160
[6]Waterland JL, McCourt O, Edbrooke L, Granger CL, Ismail H, Riedel B, Denehy L. Efficacy of Prehabilitation Including Exercise on Postoperative Outcomes Following Abdominal Cancer Surgery: A Systematic Review and Meta-Analysis. Front Surg. 2021;8

**Evaluation of the Use of Inflammatory Biomarkers in the Early Detection of Anastomotic Leakage after Oesophagectomy. A Case – Control Study**
ID: 526

**Kategorie: DGCH - Perioperative Medizin in der Chirurgie**

Mina Azer[1], Sorin Miftode[1], Maximilian Bockhorn[1], Nader EL-Sourani[1]

[1]*Carl von Ossietzky Universität Oldenburg, Oldenburg, Deutschland*

**Background:**
Postoperative anastomotic leakage (AL) remains a major complication of oesophagectomy. The development of a reliable method of early detection of AL can revolutionise the management of oesophageal carcinoma.

**Materials and methods:**
A retrospective data analysis of 147 patients who underwent Ivor-Lewis esophagectomy as a curative attempt to treat distal oesophageal **carcinoma** in our surgery department between 2010 and 2021. C reactive protein (CRP) and white blood cells (WBC) count in the postoperative days (POD) 1, 3, 5 and 8 were compared in patients with and without AL. The diagnostic accuracy of these tests was challenged against the clinical reference standard represented by CT or upper GIT endoscopy.

**Results:**
28 patients (19%) developed AL. CRP values in the POD 8 were the only parameter to qualify as a potential clinically useful test with an area under the receiver operating curve of 0.85 and a p-value of less than 0.01. We calculated the cut-off value for CRP during the POD 8 to be 10.85 mg/dl with specificity and sensitivity of 73.1% and 89.3%, respectively. CRP showed a positive predictive value of 43.9% and a negative predictive value of 96.7% at this cut-off value.

**Conclusion:**
Although an absolute diagnostic value of Postoperative estimation of serum inflammatory biomarkers could not be proved, Serum CRP could still play a role in excluding AL after oesophagectomy.Therefore, we suggest using the cut-off value of 10.85 mg/dl on POD 8 as a negative predictive value of AL and to be considered among the discharge criteria of the patients.

**FAST TRACK recovery after colorectal surgery – what is achievable at a German university hospital?**
ID: 547

**Kategorie: DGCH - Perioperative Medizin in der Chirurgie**

Sven Flemming[1], Friedrich Anger[1], Sophie Müller[1], Johanna Wagner[1], Christoph-Thomas Germer[1]

[1]*Klinik und Poliklinik für Allgemein-, Viszeral., Transplantations-, Gefäß- und Kinderchirurgie, Universitätsklinikum Würzburg, Würzburg, Deutschland*

**Background:**
There is broad international evidence that multi-professional perioperative pathways including enhanced recovery or fast track treatment are associated with a significant reduction of postoperative complications, faster postoperative recovery and mobilization resulting in a decrease of length of hospital stay. However, in Germany these fast-track programs are still not well established even if many surgeons think that their perioperative care strongly meets all fast-track criteria. The aim of this study was to implement and to validate a perioperative fast-track concept for colorectal surgery at a German university hospital.

**Materials and methods:**
The first fast-track patients from February 2022 were compared with those of a representative control group (pre-fast-track) who underwent interventions from November 2021 to January 2022. Patient care and data collection of all patients were ensured by 3 specialized fast-track nurses in daily visits. Primary endpoints were overall compliance regarding international guideline recommendations for enhance recovery, patient outcome, and length of hospital stay as well as re-admission rate.

**Results:**
A total of 60 pre-fast-track and 79 fast-track patients were included. There were no significant differences in baseline parameters such as age, sex, ASA classification and indication of operation. Interestingly, the overall compliance with guideline recommendations was only 49%. After implementation of the structured fast-track program and start of work of fast-track nurses the compliance significantly increased to 76%. Beside better overall compliance also the clinical outcome of patients after colorectal surgery improved as seen by faster tolerance of solid food (0d vs. 3d) and earlier mobilization out of patient´s bed. The rate of surgical and non-surgical postoperative complications was comparable in both patient cohorts. However, the median hospital stay could be significantly reduced from 8 days to 5 days after implementation of the fast-track pathways.

**Conclusion:**
The implementation and a successful application of a fast-track program for patients undergoing colorectal surgery is feasible and safe at a German university hospital as well. The multi-professional perioperative care driven by specialized fast-track nurses are the base for an enhanced recovery after surgical interventions.

**Risikofaktoren für die lymphatische Metastasierung von papillären Mikrokarzinomen der Schilddrüse.**
ID: 365

**Kategorie: DGCH - Sonstiges**

Eugenie Pristupa[1], Jochen Kussmann[1], Volker Fendrich[1], Kirsten Lindner[2], Thomas Burgstaller[1], Susanne Klutmann[3]

[1]*Schoen Klinik Hamburg Eilbek, Abteilung für Endokrine Chirurgie, Hamburg, Deutschland*, [2]*Krankenhaus Vilsbiburg, Endokrine Chirurgie , Vilsbiburg, Deutschland*, [3]*Universitätsklinik Hamburg Eppendorf, Abteilung für Nuklearmedizin, Hamburg, Deutschland*

**Background:**
Despite a favorable prognosis in papillary microcarcinoma (PTMC) of the thyroid gland, there are cases with metastasis. The metastasis rate is rare but relevant as it increases the risk of recurrence and early death. At the tertiary care center for endocrine surgery, we examined various factors to create a risk profile and thus improve screening methods for patients at risk.

**Materials and methods:**
For our retrospective study, we selected patients who were diagnosed with PTMC between 2008 and 2017 before or after treatment at the Endocrine Center of the Schoen Clinic Hamburg (n=389). From this collective, we selected a second group of patients who already had lymphogenic metastases at the time of diagnosis. Demographic data, (laboratory) thyroid parameters, surgical results and properties of the detected thyroid carcinomas were analyzed.

**Results:**
Of all patients, 50 (13.1%) showed lymphatic metastases. We were able to identify patient age (51 years vs. 43 years, p=<0.001), low thyroid weight (32g vs. 25g, p=0.013), elevated TSH value (0.84 vs. 1.32, p=0.003) as significant risk factors. There was also a higher risk of metastasis with an increasing number of tumor foci (p=0.019) detected. A trend without significance was shown regarding the tumor size (total and maximum). Factors such as gender (p=0.112), radiation exposure (p=0.91), smoking behavior (p=0.794), BMI (p=0.019) or localization of the metastasis (p=0.24) could not be assigned any significance.

**Conclusion:**
Our retrospective study identified young age, low thyroid weight, and high TSH levels as significant risk factors for metastasis. We also saw a significant influence on metastasis with an increasing number of tumor foci. In case of multifocality, no significant trend in tumor size (maximum and total) could be detected.

**Body mass index in pancreatic ductal adenocarcinoma (PDAC) is a critical factor for an altered bile duct microbiology after pancreaticoduodenectomy**
ID: 649

**Kategorie: DGCH - Umgang mit Problemkeimen in der Chirurgie**

Viktor H. V. Ehrlich-Treuenstätt[1,1], Jan D'haese[1], Max Weniger[1], Hanno Niess[1], Markus Guba[1], Joachim Andrassy[1], Martin Angele[1], Bernhard Renz[1], Jens Werner[1], Matthias Ilmer[1]

[1]*LMU Klinikum München, München, Deutschland*

**Background:**
While long-term survival rates among PDAC patients remain low, the perioperative morbidity rate can be influenced by various factors.
This study investigated the effects of the BMI and the bile duct (BD) microbiology on morbidity in PDAC after pancreaticoduodenectomy (PD).

**Materials and methods:**
This is a prospective observational study of 200 patients after PD due to PDAC of the pancreatic head. Patients were grouped depending on their individual BMI using the official cut-off of 25 kg/m$^{2,}$ defining adults as overweight (overweight [OW] >25, normal weight [NW] <25 kg/m$^2$). Multiple perioperative factors were analyzed, such as BD microbiology, ERCP, diabetes (DM), the wait time to surgery (TTS), and complications.

**Results:**
We found significant differences in the severity of complications between OW and NW patients classified by Clavien-Dindo (P<0.05). OW was associated with a lower rate of neoadjuvant chemotherapy (NCT) (7% in OW vs. 15% in NW; P<0.05) and a more unfavorable T status (53% T3/T4 in OW vs. 33% in NW; P<0.05). DM and TTS showed no effect on morbidity or BD microbiology, respectively (positive BD in 56% vs. 64% [DM] or 61% vs. 68% [TTS]); equal ERCP rates: 42% to 53%). Patients with NW presented with a higher number of positive BD swaps compared to OW (72% vs. 55%; P<0.05); however, we found fewer pathogens in the swap with an overall lower pathogenic potential (less Klebsiella pneumonia 0% vs. 8% / Candida albicans 0% vs. 14%; P<0.05).

**Conclusion:**
BMI is a critical factor for a higher perioperative morbidity rate in patients undergoing surgery for PDAC of the pancreatic head. Simultaneously, OW is associated with a lower rate of NCT but shorter TTS than in NW patients. In general, NW patients (BMI <25 kg/m$^2$) have a better postoperative outcome regarding the severity of complications due to a BD microbiology with lower pathogenic potential.

**The mammalian intestinal tract is not nitrogen limited**
ID: 653

**Kategorie: DGCH - Sonstiges**

Lisa Harling[1,2], Aaron Hecht[1], Junhee Lee[1], Mark Goulian[3], Gary Wu[1]

[1]*Division of Gastroenterology, Hospital of the University of Pennsylvania, Philadelphia, United States*, [2]*Department of General, Abdominal, Transplant and Visceral Surgery, University Clinic of the Otto-von-Guericke University, Magdeburg, Deutschland*, [3]*Department of Biology, University of Pennsylvania, Philadelphia, United States*

**Background**:
The human gastrointestinal tract is the home of a dense microbial ecosystem. Within the GI tract, the colon plays a key role due to its longer retention time, larger volume and neutral pH. Since the human host absorbs the majority of host-digestible nutrients in the small intestine, it is hypothesized that the colon is a nutritionally limited environment [1]. Nitrogen and carbon are at the core of anabolic processes and necessary for microbial growth. Urea, a nitrogenous waste product of mammalian hosts is secreted into the lumen of the colon and may serve as a nitrogen source for growing organisms in the gut through ammonia production by the hydrolysis of urea via bacterial urease activity. In addition to its role as a nitrogen source the urease-dependent production of ammonia can also promote bacterial growth through the neutralization of an acidic environment—A mechanism relevant to the growth of H. pylori in the stomach [2]. Herein, we explore the role urea and urease play in an either nitrogen-limited or -abundant environment using *Klebsiella pneumoniae* as a model organism in both in-vitro defined bacterial culture models and in-vivo mouse models.

**Methods and Results:**
We used either the wild type *K.pneumoniae* strain, which encodes the operon to produce urease, a strain in which the urease operon was deleted (∆urease) and is thus not able to produce urease, or a strain in which the NtrC gene was deleted (∆NtrC), which leads to the inability to utilize most alternative nitrogen sources other than ammonia or L-glutamine. In nitrogen limited media the addition of urea provides urease competent organisms a growth advantage whereas it has no effect in the strain lacking. Additionally, in nitrogen rich media the addition of urea has no impact on growth in either strain.

Overnight growth of the wild type strain in minimal media with 0mM or 18mM ammonia and 0mM, 7.5mM or 33.3mM urea added.

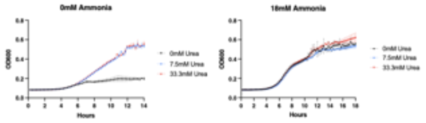

Overnight growth of the ∆urease strain in minimal media with 0mM or 18mM ammonia and 0mM, 7.5mM or 33.3mM urea added.

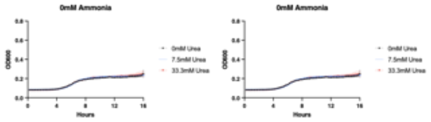

To determine the impact of urease utilization in the gut, we inoculated mice with either the WT strain, the ∆urease strain or the ∆NtrC strain after antibiotic depletion of the microbiome. We found that both the deletion mutants show no growth defect if compared with the WT strain.

Colony forming units of the wild type, ∆urease strain or the ∆NtrC strain per gram of feces after monocolonisation in mice after antibiotic depletion

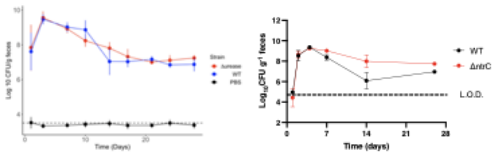

To test if *K.pneumoniae* is able to utilize urease to neutralize acidic environments in a similar manner as *H.pylori*, we inoculated an overnight culture of either the wild type or the ∆urease strain into media with an pH of 2.5 (pH7 as a control), with or without the addition of urea and incubating at 37°C for an hour before measuring the colony forming units per mL of culture. Neither the addition of urea nor the presence of urease enhance *K. pneumonia* survival and promote growth after acid shock.

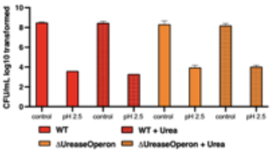

**Conclusions:**
Although our in vitro studies show that urease provides a growth advantage to *K. pneumoniae* under nitrogen limiting conditions, the lack of a colonization deficit of either the ∆urease strain or the ∆NtrC strain *in vivo* suggests that the intestinal tract is most likely not nitrogen limited due to the abundance of ammonia and/or glutamine. Additionally, unlike the protective role that urease in *H.pylori* plays as a buffer in the acidic gastric environment, urease does not protect *K. pneumoniae* against acid shock *in vitro*.

**References:**
[1] Reese, AT *et al. Nat Micro*, 2018

[2] Ansari, S. *et al. Helicobacter,* 2017
